# Evaluating the performance of large language models in sarcopenia-related patient queries: a foundational assessment for patient-centered validation

**DOI:** 10.3389/fragi.2026.1712785

**Published:** 2026-02-27

**Authors:** Tao Huang, Ben Kirk, Jacqueline Close, Jae-young Lim, Gustavo Duque, Peter Ebeling, Minghui Yang, Maoyi Tian, Chun Sing Chui, Chaoran Liu, Ning Zhang, Wing-Hoi Cheung, Ronald Man Yeung Wong

**Affiliations:** 1 Department of Orthopaedics and Traumatology, The Chinese University of Hong Kong, Hong Kong SAR, China; 2 Department of Medicine, Melbourne Medical School, The University of Melbourne, St Albans, VIC, Australia; 3 Falls, Balance and Injury Research Centre, Neuroscience Research Australia, Randwick, NSW, Australia; 4 Department of Rehabilitation Medicine, Seoul National University College of Medicine, Seoul National University Bundang Hospital, Seongnam, Republic of Korea; 5 Faculty of Medicine and Health Sciences, The McGill University Health Centre, Montreal, QC, Canada; 6 Department of Medicine, School of Clinical Sciences, Monash University, Clayton, VIC, Australia; 7 Department of Orthopaedics and Traumatology, Beijing Jishuitan Hospital, Beijing, China; 8 School of Public Health, Harbin Medical University, Harbin, China

**Keywords:** ChatGPT, deepseek, Gemini, large language models, sarcopenia

## Abstract

**Background:**

Large Language Models (LLMs) have shown promise in clinical applications but their performance in specialized areas such as sarcopenia remains understudied.

**Methods:**

A panel of sarcopenia clinician researchers developed 20 standardized patient-centered questions across six clinical domains. Each question was input into all three LLMs, and responses were anonymized, randomized, and independently assessed by three clinician researchers. Accuracy was graded on a four-point scale (“Poor” to “Excellent”), and comprehensiveness was evaluated for responses rated “Good” or higher using a five-point scale.

**Results:**

All LLMs achieved good performance, with no responses rated “Poor” across any domain. Deepseek had the longest and most detailed responses (mean word count: 583.75 ± 71.89) and showed superior performance in “risk factors” and “prognosis.” ChatGPT provided the most concise replies (359.5 ± 87.89 words, p = 0.0011) but achieved the highest proportion of “Good” ratings (90%). Gemini excelled in “pathogenesis” and “diagnosis” but received the most critical feedback in “prevention and treatment.” Although trends in performance differences were noted, they did not reach statistical significance. Mean comprehensiveness scores were also similar across models (Deepseek: 4.017 ± 0.77, Gemini: 3.97 ± 0.88, ChatGPT: 3.953 ± 0.83; p > 0.05).

**Conclusion:**

Despite minor differences in performance across domains, all three LLMs demonstrated acceptable accuracy and comprehensiveness when responding to sarcopenia-related queries. Their comparable results may reflect similarly recent training data and language capabilities. These findings suggest that LLMs could potentially serve as a valuable tool in patient education and care on sarcopenia. This study provides an initial, expert-based assessment of LLM information quality regarding sarcopenia. While the responses demonstrated good accuracy, this evaluation focuses on content correctness from a clinical perspective. Future research must complement these findings by directly engaging older adult cohorts before clinical implementation can be considered. However, human oversight remains essential to ensure safe and appropriate assessment and individually tailored advice and management.

## Introduction

Large Language Models (LLMs) represent a paradigm shift in natural language processing (NLP), offering unprecedented capabilities in medical text understanding and generation. These deep neural networks, built upon deep learning architectures such as the Transformer architectures (Ashish Vaswani et al., 2017), have demonstrated potential in processing medical terminology, extracting clinical insights, and generating coherent biomedical text. In clinical informatics, LLMs models such as OpenAI’s GPT-4.5 (Achiam et al., 2023), Google’s Gemini (Anil et al., 2023), and DeepSeek (Liu et al., 2023) are commonly used for tasks including automatic report generation, response to clinical questions, cohort identification, and real-time decision support (Singhal et al., 2023). Previous studies have reported achieving 94% accuracy in automated documentation (Kamran et al., 2024) and 71.4% diagnostic concordance with clinicians (Mittermaier et al., 2023). In this context, LLMs have emerged also as a focal point of medical AI research.

Based on its GPT-3.5/4 architecture, OpenAI’s ChatGPT demonstrates considerable promise in medical applications, owing to its proficiency in comprehending and generating contextually clinical language (Kung et al., 2023). Its utility extends across clinical decision support, medical education, documentation, and patient communication (Ayers et al., 2023). Deepseek, a China-developed open-source large language model, has exhibited robust capabilities in clinical reasoning tasks and diagnostic applications (Liu, 2025). Google DeepMind’s Gemini is an advanced multimodal LLM that can integrate text and image data for clinical use. It shows strong performance in diagnostic interpretation, automated radiology reporting, and evidence-based treatment recommendations—achieving accuracy comparable to expert radiologists.

Sarcopenia, a disease characterized by progressive and generalized loss of skeletal muscle mass, strength, and function, is a major contributor to frailty, falls, fractures and mortality in the older population (Cruz-Jentoft and Sayer, 2019; Kirk et al., 2024). Prevalence estimates of sarcopenia range from 9.9% to 40.4%, depending on the definition employed, and the adverse socioeconomic impact of this muscle disease is projected to increase with the population (Mayhew et al., 2019). AI-driven platforms have potential to personalize intervention strategies for sarcopenia which can include nutrition, resistance training, and telehealth-based follow-up, optimizing patient adherence and outcomes. However, current evidence suggests that LLMs may generate seemingly plausible yet medically inaccurate outputs, particularly when interpreting nuanced clinical data or providing therapeutic recommendations (van Dis et al., 2023). Their clinical applicability remains insufficiently validated, necessitating rigorous evaluation before implementation.

In this study, we assess three widely accessible and commonly utilized large language models (LLMs), ChatGPT-4.5, Deepseek-V3, and Gemini 2.0, by analyzing their responses to frequently asked patient questions related to sarcopenia. ChatGPT-4.5 (OpenAI) is based on the GPT-4 architecture, optimized for language generation and dialogue, relying primarily on pre-trained static knowledge without built-in search. Deepseek-V3 is an open-source model leveraging Retrieval-Augmented Generation (RAG), integrating external search results to improve factual accuracy. Yet Gemini 2.0 (Google DeepMind) combines large language modeling with native Google Search capabilities, allowing real-time access to updated information. These differences affect the performance in terms of accuracy, relevance, and timeliness when answering medical-related queries. This study aims to analyze the accuracy and comprehensiveness of LLMs in addressing sarcopenia-related inquiries, thereby identifying whether the information is potentially reliable for patient support.

## Methods

### Study design

This study was conducted between February 2025 and May 2025, at the Department of Orthopaedics and Traumatology, The Chinese University of Hong Kong, Hong Kong, China. Clinician researchers (JC, GD, PE, BK, RMYW and JY) collaborated to generate a set of sarcopenia related questions. The process started with collecting information and questions from reputable online health information sources, including the National Institute on Aging (NIA) from the United States National Institutes of Health (NIH) and Birmingham Biomedical Research Center (NIHR). 25 questions were generated in various aspects of sarcopenia, including its definition, causes, diagnosis, prevention, treatment, and impact on daily life. The panel then refined the questions and selected 20 most frequently asked in clinical practice based on their professional knowledge and clinical experience. To further analyze the capabilities of LLM tools under different subject matters, questions were categorized into 6 domains: Pathogenesis, Risk factors, Clinical presentation, Diagnosis, Treatment and Prevention, and Prognosis.

**FIGURE 1:**
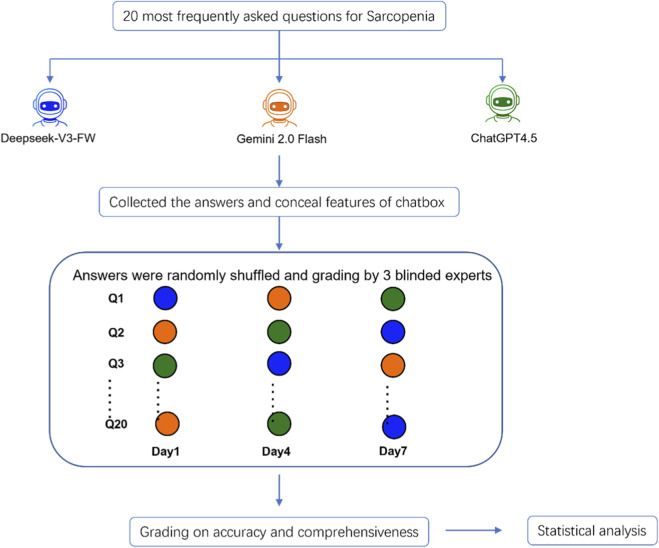
Overall study flow for the LLM-chatbot assessment.

From April 1 to 1 May 2025, responses to these queries were generated by the 3 different LLM tools with the latest versions including ChatGPT-4.5, Deepseek-V3, and Gemini 2.0. Both Deepseek-V3, and Gemini 2.0 are publicly accessible at no charge, whereas ChatGPT-4.5 required a paid subscription. The overall study process is shown in Figure 1. Twenty pre-selected questions were individually input into each of the LLM tools. To minimize memory retention bias, the conversation was reset after each question for all LLM tools. To blind the evaluators to the identity of the chatbots, all responses were formatted as plain text, removing any identifying features. These responses were then randomly shuffled before being presented to sarcopenia researchers for evaluation. Grading was conducted over 3 separate sessions, each spaced 72 h apart, to reduce fatigue and potential bias.

### Accuracy evaluation

The grading panel consisted of 3 clinician researchers (JC, BK and JYL), each with at least 30 publications related to the field of sarcopenia or geriatrics and gerontology. To ensure objectivity, the identities of the LLM tools were concealed from the evaluators (e.g., “Source of Website” from Gemini). The graders independently assessed the accuracy of each response generated by the LLM tools, using a four-point scale as follows: 1. Unsatisfactory (Poor) for responses containing significant factual inaccuracies or omissions that could lead to patient misunderstanding, inappropriate decision-making, or potential harm. 2. Limited (Moderate) for responses containing moderate inaccuracies or ambiguities that are unlikely to directly harm the patient but may lead to suboptimal clinical outcomes without clarification. 3. Proficient (Good) for responses mostly accurate and with only minor errors that are unlikely to mislead the patient or affect clinical decisions. Minimal clarification may be needed for optimal understanding. 4. Exemplary (Excellent) for responses entirely accurate, evidence-based, and clearly communicated, requiring no further clarification. They align with best clinical practices and support informed patient decision-making. The sum of the scores from different graders decided the total score for each LLM tools.

A majority consensus approach was employed to determine the final scores for each LLM’s response, based on the most frequently assigned grade amongst the three graders. In cases where no consensus was reached (i.e., each grader assigned a different score), the LLM’s response was assigned the lowest score. For scores lower than 2, graders would provide comments on its limitations.

### Comprehensiveness evaluation

For chatbot responses that received a ‘good’ rating by majority consensus, the graders conducted an additional evaluation to assess their comprehensiveness. This was done using a five-point scale: (Ashish Vaswani et al., 2017): ‘Deficient’ for responses lacking critical details, omitting key concepts, or fails to address the core inquiry and requires substantial revision.; (Achiam et al., 2023).; ‘Basic’ for those providing essential information but lacks depth, nuance, or supporting details; (Anil et al., 2023); ‘Developing’ for responses covering major aspects with some elaboration but has gaps in secondary details or context; (Liu et al.); ‘Proficient’ for those addressing the inquiry comprehensively with minor omissions in less critical areas; and (Singhal et al., 2023) ‘Exemplary’ for responses thoroughly exploring the topic with robust detail, contextualization, and no significant omissions. The overall comprehensiveness score was calculated by averaging the individual scores assigned by each grader across all responses rated as ‘good’.

### Statistical analysis

Statistical analyses were performed using Prism (Version 9.0, GraphPad Software, Boston, MA, USA). Differences of responses from the three LLM tools were assessed using one-way ANOVA, followed by Tukey’s *post hoc* test. For comparisons involving word count, total accuracy scores, and comprehensiveness scores, the Kruskal-Wallis Rank Sum test was applied, with Dunn’s multiple comparisons test used for *post hoc* analysis. A p-value <0.05 was considered statistically significant.

## Results

**FIGURE 2:**
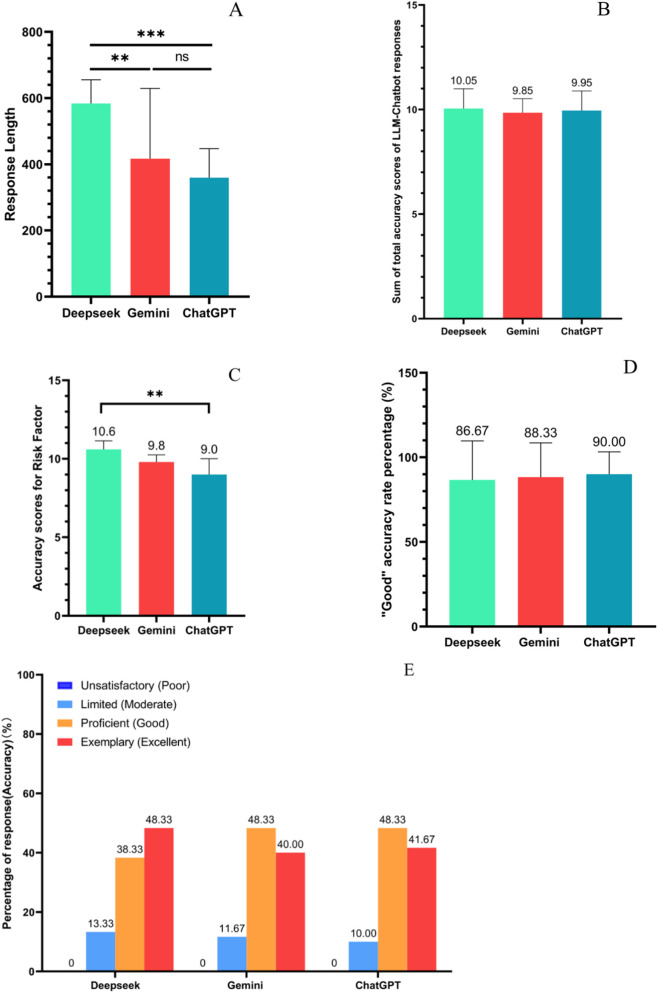
**(A)** Response length (words) for three LLM tools. **(B)** Summary of total accuracy scores of LLM-Chatbot responses to sarcopenia-related questions, as assessed by three clinician researchers. **(C)** Accuracy scores for Risk Factor response. **(D)** “Good” accuracy rate percentage. **(E)** Percentage of accuracy among all response.

The responses of 3 LLMs to 20 standardized sarcopenia-related questions and the sum of the length of their responses for single question and for different characters were recorded (Table 1). The mean ± standard deviation (SD) of the word count was 583.75 ± 71.89 for Deepseek-V3 (longer than Gemini, p = 0.0011 and ChatGPT, p < 0.0001), 417.3 ± 212.06 for Gemini 2.0, and 359.5 ± 87.89 for ChatGPT4.5. The mean character count was 1958.17 ± 927.32 for Deepseek-V3, 1406.07 ± 818.74 for Gemini 2.0, and 1199.5 ± 541.22 for ChatGPT4.5. Figure 2A shows that Deepseek tended to use more words and had longer explanations in response to questions than Gemini and ChatGPT whilst the ChatGPT had the shortest response length.

**TABLE 1:** Response length from LLM tools’ response to sarcopenia-related questions.

Chatbots	Answer length (words)			Answer length (characters)		
	Mean (SD)	Minimum	Maximum	Mean (SD)	Minimum	Maximum
Deepseek	583.75 ± 71.89	436	742	1958.17 ± 927.32	1115.00	3140.00
Gemini	417.3 ± 212.06	173	904	1406.67 ± 818.74	715.00	2910.00
ChatGPT	359.5 ± 87.89	165	452	1199.5 ± 541.22	576.00	1773.00

Abbreviation: LLM, large language model; SD, standard deviation.

Figure 2B illustrates the summary of total accuracy scores of LLM’ responses to sarcopenia-related question. Deepseek had a superior average total accuracy score of 10.05 compared to Gemini at 9.85 and ChatGPT at 9.95 but the results showed no significant difference. For risk factors (Figure 2C), Deepseek showed a higher accuracy score compared to ChatGPT (p = 0.0098). Figure 2D showed all 3 LLM tools had 0 ‘Poor score’ from the different graders. Deepseek got the highest Excellent score (48.33%) and Moderate score (13.33%) and ChatGPT got the highest “Good” rate (90%). However, no significant difference was found amongst the 3 LLM tools. Figure 2E demonstrates that the 3 LLM tools got similar results for the “Good” accuracy rate percentage which was 86.67% for Deepseek, 88.33% for Gemini and 90% for ChatGPT.

**FIGURE 3:**
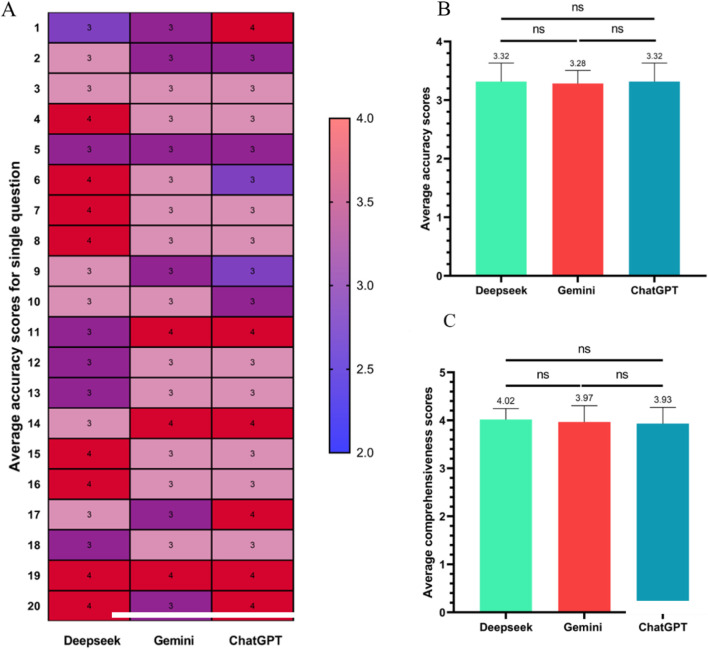
The performance of the 3 LLMs in terms of average accuracy score as assessed by 3 orthopedics. Heatmap **(A)** of individual accuracy score; **(B)** presents the mean accuracy scores; **(C)** illustrates the average comprehensiveness scores. ns = not significant.

Figure 3 depicts the distribution of accuracy assessments derived from expert consensus. For some questions including questions 4, 14, 19, 20, ChatGPT got higher red areas, indicating high accuracy scores, whilst for some questions including questions 3, 9, 13, most groups show purple areas, suggesting moderate accuracy scores. The bar charts (Figure 3B) indicate that the average accuracy scores for Deepseek, Gemini, and ChatGPT were similar, with no statistically significant differences. Figure 3C indicates that in terms of comprehensiveness, Deepseek, Gemini, and ChatGPT have similar scores, with no significant differences.

Table 2 summarizes an analysis of consensus-based accuracy scores across the six sarcopenia–related categories. None of the LLM tools had a “poor” rating amongst any domain. Deepseek achieved the highest performance in the domains of “risk factor” and “prognosis” with 100% ratings of ‘good’ or above while Gemini got 100% ratings of ‘good’ or above in “pathogenesis” and “diagnosis”. Although ChatGPT demonstrated the poorest performance in “risk factors”, it still achieved the best “Good” rating across most evaluated domains including “pathogenesis”, “clinical presentation”, “diagnosis” and “prognosis”. Deepseek received the most “Moderate” reviews for its response in “pathogenesis”, “clinical “presentation” and “diagnosis” and Gemini got the most negative reviews in “prevention and treatment”.

**TABLE 2:** Consensus accuracy score of LLM’s response to sarcopenia-related questions.

Chatbots	Response comprehensiveness		
	n	Mean ± SD	Median
Deepseek	20	4.017 ± 0.77	4
Gemini	20	3.97 ± 0.88	4
ChatGPT	19	3.953 ± 0.83	4

Comprehensiveness ratings for all evaluated responses—excluding those rated as “poor”—are summarized in Table 3. Deepseek had a score of 4.017 ± 0.77 and Gemini got a score of 3.97 ± 0.88. With one response rated as “poor”, ChatGPT received a score of 3.953 ± 0.83. However, no statistically significant difference was found amongst the evaluations.

**TABLE 3:** Comprehensiveness assessment of LLM’s response to sarcopenia-related questions.

Domain	No. of questions	Deepseek				Gemini				ChatGPT			
		Poor	Moderate	Good	Excellent	Poor	Moderate	Good	Excellent	Poor	Moderate	Good	Excellent
Pathogenesis	5	0 (0)	2 (13)	8 (53)	5 (33)	0 (0)	0 (0)	13 (87)	2 (13)	0 (0)	0 (0)	11 (73)	4 (27)
Risk factors	5	0 (0)	0 (0)	7 (47)	8 (53)	0 (0)	2 (13)	7 (47)	6 (40)	0 (0)	5 (33)	5 (33)	5 (33)
Clinical presentation	2	0 (0)	2 (33)	2 (33)	2 (33)	0 (0)	1 (17)	1 (17)	4 (67)	0 (0)	0 (0)	3 (50)	3 (50)
Diagnosis	2	0 (0)	2 (33)	1 (17)	3 (50)	0 (0)	0 (0)	3 (50)	3 (50)	0 (0)	0 (0)	3 (50)	3 (50)
Prevention and treatment	4	0 (0)	2 (17)	3 (25)	7 (58)	0 (0)	3 (25)	3 (25)	6 (50)	0 (0)	1 (8)	5 (42)	6 (50)
Prognosis	2	0 (0)	0 (0)	2 (33)	4 (67)	0 (0)	1 (17)	2 (33)	3 (50)	0 (0)	0 (0)	2 (33)	4 (67)

Numbers represent quantities corresponding to domains/categories. Numbers within brackets are percentages (%). Percentages may not add up to 100% due to rounding.

## Discussion

Given that the prevalence of sarcopenia is highest among older people (Sayer and Cruz-Jentoft, 2022), there may be difficulties to assess the reliability of online health information. Our study focuses on a real-world condition for which concerned patients may seek support from online AI tools. These questions may often fall outside the scope of precise answers in routine primary care. This highlights the importance of assessing the accuracy and reliability of LLM chatbot responses in practical, patient-centered contexts. Through a rigorous study design including appropriate masking, randomization, and comprehensive evaluation, an objective analysis was executed. Our main findings indicated that the 3 LLM tools can respond to commonly asked questions in everyday practice.

Due to the capacity for information collecting and generating human-like language, LLMs have been investigated as potential tools for delivering health-related information to patients (Lee et al., 2023). Amongst the LLM tools tested, Deepseek consistently generated the longest and most comprehensive responses (Table 1), with a mean word count significantly higher than both Gemini and ChatGPT. This suggests that DeepSeek tends to offer more comprehensive explanations, which could be beneficial for users seeking in-depth information. In Ayers’s study (Ayers et al., 2023), AI tools tended to generate more verbose responses compared to human experts, which could either enhance understanding or risk information overload. In contrast, ChatGPT produced the shortest responses, indicating a briefer style which aligns with findings from another study (Singhal et al., 2023), that shorter responses were often rated as more clinically useful for quick decision-making and more user-friendly for patients for quick reference. Additionally, in a randomized study evaluating responses to real patient questions, ChatGPT’s answers were rated higher than clinicians’ in both quality and empathy (Ayers et al., 2023), highlighting its potential to enhance clinician and patient communication.

For the accuracy and completeness of responses, all of 3 LLM tools (Figure 2) demonstrated high accuracy without response rated as “poor” which consisted previous studies that LLM tools have the potential to respond to questions about general or specific patient populations. These include myopia care (Lim et al., 2023), cataract care (Su et al., 2025) and osteoarthritis (Cao et al., 2024), and all indicating a high level of accuracy and completeness in addressing common patient questions. However, Deepseek achieved the highest average accuracy score compared to ChatGPT and Gemini. Interestingly, while ChatGPT received the highest “Good” ratings, DeepSeek had the most “Excellent” responses. This finding matched the result from Rajpurkar et al.‘s study (Rajpurkar and Lungren, 2023), where some LLMs excelled in breadth (covering many points comprehensively) whilst others stood out in depth (providing fewer but more authoritative answers). This suggests that model selection should depend on whether the user priority is reliability (ChatGPT) or depth (DeepSeek). While our study establishes a necessary foundation by confirming information accuracy, the question of whether these responses are truly understandable and useful to patients remains unanswered and requires dedicated investigation using qualitative and patient-centered methodologies.

Across the six domains, Deepseek performed well in the “risk factors” and “prognosis” categories, with 100% of its responses rated as “good” or higher. This suggests that Deepseek may possess strengths in content areas related to disease progression and contributing factors. Gemini, on the other hand, excelled in “pathogenesis” and “diagnosis,” achieving similarly high ratings, which may indicate its robustness in explaining biological mechanisms and identifying clinical features. Yet in Rajpurkar’s study, DeepSeek-R1 achieving 92% accuracy in oral pathology diagnosis versus GPT-4o′s 87.2% (Wu et al., 2025). A surgical case series (100 complex cases) also showed equivalence to GPT-4 in the final diagnosis and a broader differential list, though inclusion rates were lower (Chan et al., 2025). The heterogeneity among study findings can be attributed to variations in chatbot versions and the diversity of clinical domains examined. Our findings also align with recent studies exploring the use of LLMs in other clinical contexts. For instance, Antaki et al. (2023) evaluated ChatGPT’s ability to respond to ophthalmology questions and found that whilst it performed reasonably well overall, it struggled with more specialized content, such as retinal diseases or surgical decision-making.

Although a trend was observed in overall accuracy score for all domains in other study, however, they used the same tool of different generations (like ChatGPT3.5 and 4.0) and conducted a longitudinal comparison. In this study, we conducted a horizontal comparison, and the difference was not statistically significant. We believe this may be attributed to the use of recently developed LLMs in this study, which have similarly updated training data (Achiam et al., 2023; Anil et al., 2023; Liu et al., 2023) and, consequently, demonstrate comparable accuracy in their responses. Prior studies (Chen et al., 2025; Clusmann et al., 2023) have also noted that newer LLMs tend to converge in performance metrics, particularly on well-established topics. Moreover, the diagnosis and definition of sarcopenia is not consistent in different regions around the world. In Oceania and Europe, the European Working Group on Sarcopenia in Older People (EWGSOP) and EWGSOP2 (Cruz-Jentoft et al., 2019) are often used and in Asia, the Asian Working Group on Sarcopenia (2019, AWGS2) (Chen et al., 2020) was proposed. The researched involved in the study were from different countries, which may have introduced variability in their evaluation criteria.

This study has several limitations. First, a standardized definition and diagnostic criteria for sarcopenia were lacking. Variations in assessment protocols may lead to difference in expert grading of the same clinical question. Second, the researcher-designed questionnaire may not comprehensively address all patient concerns. Lastly, while specialists evaluated the responses for accuracy and comprehensiveness, patient perspectives were not measured. This study therefore focused on the “expert accuracy” dimension of information quality. The critical questions of whether these responses are comprehensible, actionable, and appropriate for older adult remain unanswered. Although expert-rated accuracy does not guarantee patient-centered usefulness, establishing information accuracy is a necessary prerequisite before patient-centered utility can be meaningfully assessed.

Building on our findings, the practical use of LLMs in multidisciplinary sarcopenia care requires a clear understanding of their role. In clinical workflows, LLMs can act as supportive tools to help healthcare teams work more efficiently. For clinical management, they could potentially assist by quickly reviewing electronic health records to flag those at high risk of sarcopenia for further assessment, or by drafting summary notes to assist the diagnosis and treatment. For patient education, LLMs could generate easy-to-understand explanations of the condition, create personalized exercise or nutrition guides in simple language, or prepare educational materials in different languages.

Building directly on this expert-based evaluation, subsequent research should prioritize the patient perspective. Ideally, future studies would recruit representative cohorts of older adults, including those with varying levels of health literacy, to directly assess how they interact with and perceive LLM-generated information. This would involve recruiting cohorts of older adults at risk for or concerned about sarcopenia, in order to assess the usability, comprehensibility, and perceived value of LLM-generated information. Further evaluation should also monitor the consistency of responses over time and test multimodal capabilities, such as integrating text with imaging or laboratory data, to better approximate clinical decision-making contexts. Such comprehensive validation remains essential before these tools can be safely integrated into routine sarcopenia management.

In conclusion, our findings provide promising evidence for the potential role of LLMs in supplementing sarcopenia education and management in primary care. However, real-world application must be approached with caution, emphasizing quality control, and ensuring that final medical decisions are made under human supervision. Given that sarcopenia management requires multidisciplinary knowledge from pathophysiology to rehabilitation, our results suggest that no single LLM is universally superior. For patient education or academic use, DeepSeek’s detailed explanations could enhance understanding. For point-of-care decision support, ChatGPT’s concise answers may save time and easy to learn. For diagnostic uncertainty, Gemini’s strength in pathogenesis could help with differential diagnosis. Further refinements in model training, particularly in weaker domains, could enhance their utility in medical practice and research. Future research should focus on real-world applications, user-centered evaluations, and the incorporation of clinical oversight to ensure these LLM tools can be safely and effectively deployed in the field of sarcopenia.

## Data Availability

The original contributions presented in the study are included in the article/Supplementary Material, further inquiries can be directed to the corresponding author.
