# Supplementary material for: Evaluating the performance of large language models in sarcopenia-related patient queries: a foundational assessment for patient-centered validation
Source: Front Aging. 2026 Feb 27;7:1712785. doi: 10.3389/fragi.2026.1712785 (PMC12983229; doi:10.3389/fragi.2026.1712785)
Supplement: Supplementary file 1 [file Table1.docx]

Supplementary Table 1

**Twenty commonly asked sarcopenia-related questions**

| **Topic: Pathogenesis** | |
| --- | --- |
| 1 | What is sarcopenia? |
| 2 | What are the causes of sarcopenia? |
| 3 | Can sarcopenia be caused by other diseases or drugs? |
| 4 | Who is prone to sarcopenia? |
| 5 | At what age does sarcopenia typically begin? |
| **Topic: Risk factors** | |
| 6 | What are the risk factors for developing sarcopenia? |
| 7 | Is sarcopenia related to malnutrition or a sedentary lifestyle? |
| 8 | Can medication use for weight loss in obesity cause sarcopenia? |
| 9 | Can sarcopenia be associated with osteoporosis? |
| 10 | Can sarcopenia be associated with obesity? |
| **Topic: Clinical presentation** | |
| 11 | What are the early signs and symptoms of sarcopenia? |
| 12 | What are late signs and symptoms of sarcopenia? |
| **Topic: Diagnosis** | |
| 13 | How do we diagnose sarcopenia? |
| 14 | What should I do if I suspect I have sarcopenia? |
| **Topic: Prevention and treatment** | |
| 15 | Can sarcopenia be prevented? |
| 16 | What is the best treatment for sarcopenia? |
| 17 | Are there any medications to treat sarcopenia? |
| 18 | What types of exercise or nutrition are best for me to prevent or treat sarcopenia? |
| **Topic: Prognosis** | |
| 19 | Can sarcopenia lead to fall, disability, or fractures? |
| 20 | How does sarcopenia impact my daily life? |

Supplementary Table 2 Responses to sarcopenia-related Questions.

| **No** | **Questions** | **Deepseek-V3-FW** | **Gemini 2.0 Flash** | **ChatGPT4.5** |
| --- | --- | --- | --- | --- |
| **Topic: Pathogenesis** | | | | |
| 1 | What is sarcopenia? | **Sarcopenia** is a condition characterized by the progressive loss of **muscle mass**, **strength**, and **function** that occurs with aging. It is a natural part of aging but can lead to significant health issues if not managed properly. Here’s a detailed overview: **Key Features of Sarcopenia**  1. **Muscle Mass Loss**: Reduction in the size and number of muscle fibers. 2. **Muscle Strength Decline**: Decreased ability to generate force, making it harder to perform daily activities. 3. **Functional Impairment**: Reduced physical performance, including slower walking speed, difficulty climbing stairs, or getting up from a chair.  **Causes of Sarcopenia**  1. **Aging**:    - **Hormonal Changes**: Declines in hormones like testosterone, estrogen, and growth hormone.    - **Reduced Protein Synthesis**: The body becomes less efficient at building and repairing muscle tissue.    - **Mitochondrial Dysfunction**: Impaired energy production in muscle cells. 2. **Physical Inactivity**:    - Lack of exercise accelerates muscle loss. 3. **Poor Nutrition**:    - Inadequate protein intake or malnutrition contributes to muscle wasting.    - Deficiencies in nutrients like vitamin D and calcium. 4. **Chronic Diseases**:    - Conditions like diabetes, heart disease, cancer, or kidney disease can worsen sarcopenia. 5. **Inflammation**:    - Chronic low-grade inflammation associated with aging can break down muscle tissue. 6. **Lifestyle Factors**:    - Smoking, excessive alcohol use, and poor sleep can exacerbate muscle loss.  **Risk Factors for Sarcopenia**  1. **Age**: Most common in adults over **50**, with risk increasing significantly after **60**. 2. **Gender**: Women may be at higher risk due to hormonal changes during menopause. 3. **Sedentary Lifestyle**: Lack of physical activity accelerates muscle loss. 4. **Chronic Illnesses**: Conditions like diabetes, COPD, or rheumatoid arthritis. 5. **Poor Nutrition**: Low protein intake or malnutrition. 6. **Genetics**: Family history of muscle weakness or low muscle mass.  **Symptoms of Sarcopenia**  1. **Weakness**: Difficulty lifting objects, climbing stairs, or standing up from a chair. 2. **Fatigue**: Feeling tired more easily during physical activities. 3. **Reduced Mobility**: Slower walking speed or difficulty balancing. 4. **Weight Loss**: Unintentional loss of muscle mass may lead to weight loss. 5. **Increased Fall Risk**: Weaker muscles and poor balance increase the likelihood of falls.  **Diagnosis** Sarcopenia is diagnosed through a combination of:   1. **Muscle Mass Measurement**:    - Dual-energy X-ray absorptiometry (DEXA) or bioelectrical impedance analysis (BIA). 2. **Strength Assessment**:    - Handgrip strength or leg press tests. 3. **Functional Tests**:    - Gait speed or timed chair stands.  **Prevention and Management**  1. **Exercise**:    - **Resistance Training**: Weightlifting or resistance band exercises to build muscle.    - **Aerobic Exercise**: Walking, swimming, or cycling to improve overall fitness.    - **Balance Training**: Yoga or tai chi to reduce fall risk. 2. **Nutrition**:    - **Protein Intake**: Aim for **1.0-1.2 grams of protein per kilogram of body weight per day** (higher for older adults).    - **Essential Nutrients**: Ensure adequate intake of vitamin D, calcium, and omega-3 fatty acids. 3. **Lifestyle Changes**:    - **Quit Smoking**: Smoking accelerates muscle loss.    - **Limit Alcohol**: Excessive alcohol consumption can impair muscle repair.    - **Sleep Well**: Aim for 7-9 hours of quality sleep per night to support muscle recovery. 4. **Medical Interventions**:    - **Hormone Therapy**: In some cases, hormone replacement therapy may be considered.    - **Medications**: Research is ongoing, but no specific medications are currently approved for sarcopenia.  **Importance of Early Intervention** Early detection and management of sarcopenia can:   - Improve muscle strength and function. - Reduce the risk of falls and fractures. - Enhance quality of life and independence.  **Summary** Sarcopenia is a common age-related condition characterized by the loss of muscle mass, strength, and function. It can be managed and even prevented through regular exercise, proper nutrition, and a healthy lifestyle. If you’re concerned about muscle loss, consult a healthcare provider for personalized advice and interventions. | Sarcopenia is a condition characterized by the gradual loss of muscle mass, strength, and function, primarily affecting older adults. It is a progressive and generalized skeletal muscle disorder associated with an increased likelihood of adverse outcomes, including falls, fractures, physical disability, and mortality.  Here's a breakdown of key aspects:   - **Definition:** Sarcopenia is defined as a progressive and generalized skeletal muscle disorder involving the loss of muscle mass, strength, and function. - **Symptoms:** Common symptoms include muscle weakness, loss of stamina, difficulty performing daily activities, slow walking, trouble climbing stairs, poor balance, falls, and a decrease in muscle size. - **Impact:** Sarcopenia can significantly impact the quality of life, leading to reduced ability to perform daily tasks, loss of independence, increased frailty, falls, fractures, and a higher risk of hospitalization, surgeries, and complications. - **Causes and Risk Factors:** While primarily associated with aging, other factors like physical inactivity, poor nutrition, hormonal changes (testosterone and insulin-like growth factor), and certain underlying health conditions can contribute to sarcopenia. - **Diagnosis:** Diagnosis involves assessing muscle strength, muscle mass, and physical performance.   Related searches:  [sarcopenia definition](https://www.google.com/search?q=sarcopenia+definition&client=app-vertex-grounding-quora-poe) | Your description is accurate. Here's a refined definition:  **Sarcopenia** is a progressive skeletal muscle disorder characterized by the gradual loss of muscle mass, muscle strength, and physical performance, primarily associated with aging. It increases the risk of physical disability, falls, fractures, and decreased quality of life, particularly in older adults.  **Key features of sarcopenia include:**   - **Loss of muscle mass (atrophy)** - **Reduced muscle strength** - **Impaired muscle function and mobility**   **Risk factors:**   - Aging (primary factor) - Sedentary lifestyle - Poor nutrition, particularly insufficient protein intake - Chronic inflammation and chronic diseases - Hormonal changes (e.g., decline in testosterone, estrogen, growth hormone)   **Diagnosis:** Typically involves:   - Assessment of muscle strength (e.g., handgrip strength) - Muscle quantity measurement (e.g., DEXA, bioelectrical impedance, CT, MRI) - Evaluation of physical performance (e.g., gait speed, timed up-and-go test)   **Management strategies:**   - Resistance training and physical activity - Adequate nutrition, especially protein and vitamin D supplementation - Pharmacological interventions (currently limited but emerging treatments are under study)   Early identification and intervention can significantly mitigate sarcopenia's negative impacts on health and quality of life. |
| 2 | What are the causes of sarcopenia? | Sarcopenia, the age-related loss of muscle mass, strength, and function, is caused by a combination of biological, lifestyle, and environmental factors. Here are the primary causes and contributing factors: 1. **Aging**  - **Hormonal Changes**: Decline in hormones like testosterone, estrogen, and growth hormone, which are essential for muscle maintenance. - **Reduced Protein Synthesis**: Aging slows the body’s ability to build and repair muscle tissue. - **Mitochondrial Dysfunction**: Impaired energy production in muscle cells contributes to muscle weakness.  2. **Physical Inactivity**  - **Sedentary Lifestyle**: Lack of exercise accelerates muscle loss. - **Reduced Muscle Use**: Immobility due to illness, injury, or lifestyle choices leads to muscle atrophy.  3. **Poor Nutrition**  - **Inadequate Protein Intake**: Protein is essential for muscle repair and growth; insufficient intake accelerates muscle loss. - **Caloric Deficiency**: Undereating can lead to muscle wasting, especially in older adults. - **Vitamin D Deficiency**: Low vitamin D levels are linked to muscle weakness and sarcopenia.  4. **Chronic Diseases**  - **Diabetes**: Insulin resistance and inflammation can impair muscle health. - **Heart Failure**: Reduced blood flow and oxygen delivery to muscles. - **Chronic Kidney Disease**: Leads to muscle wasting due to metabolic imbalances. - **Cancer**: Cachexia (wasting syndrome) associated with cancer accelerates muscle loss. - **Chronic Inflammation**: Conditions like rheumatoid arthritis or inflammatory bowel disease contribute to muscle breakdown.  5. **Hormonal Imbalances**  - **Low Testosterone**: Reduced levels of this hormone, which promotes muscle growth. - **Low Estrogen**: Postmenopausal women are at higher risk due to declining estrogen levels. - **Growth Hormone Deficiency**: Impairs muscle repair and growth.  6. **Neurological Factors**  - **Motor Neuron Loss**: Aging leads to a decline in the number of nerve cells that control muscle movement. - **Reduced Nerve-Muscle Communication**: Impairs muscle activation and strength.  7. **Inflammation**  - **Chronic Low-Grade Inflammation**: Aging is associated with increased levels of inflammatory markers like cytokines, which can break down muscle tissue.  8. **Medications**  - **Corticosteroids**: Long-term use can lead to muscle wasting. - **Other Drugs**: Some medications for chronic conditions may have side effects that contribute to muscle loss.  9. **Lifestyle Factors**  - **Smoking**: Reduces blood flow to muscles and impairs muscle repair. - **Alcohol Consumption**: Excessive drinking can damage muscle tissue and impair nutrient absorption.  10. **Genetic Factors**  - **Family History**: Genetic predisposition may play a role in how quickly muscle mass is lost with age.  11. **Immune System Changes**  - **Immune Senescence**: Aging weakens the immune system, leading to chronic inflammation and muscle breakdown.  12. **Environmental Factors**  - **Social Isolation**: Lack of social engagement can lead to reduced physical activity and poor nutrition. - **Limited Access to Healthcare**: Delayed diagnosis or treatment of contributing conditions.  Prevention and Management:  1. **Exercise**:    - Resistance training (e.g., weightlifting) to build muscle.    - Aerobic exercise (e.g., walking, swimming) to maintain overall health. 2. **Nutrition**:    - Adequate protein intake (1.0-1.2 g/kg of body weight per day for older adults).    - Balanced diet rich in vitamins, minerals, and antioxidants. 3. **Hormone Therapy**:    - Testosterone or estrogen replacement therapy if levels are low (under medical supervision). 4. **Manage Chronic Conditions**:    - Proper treatment of diseases like diabetes, heart failure, or kidney disease. 5. **Avoid Smoking and Excessive Alcohol**:    - Promote overall health and muscle maintenance. 6. **Physical Activity**:    - Stay active to prevent muscle atrophy and maintain strength. 7. **Supplements**:    - Vitamin D and omega-3 fatty acids may help reduce inflammation and support muscle health.  Summary: Sarcopenia is caused by a combination of aging, inactivity, poor nutrition, chronic diseases, and other factors. While it’s a natural part of aging, proactive measures like regular exercise, a balanced diet, and managing health conditions can slow its progression and maintain muscle strength and function. If you’re concerned about sarcopenia, consult a healthcare provider for personalized advice and interventions. | Sarcopenia is primarily associated with aging, but several factors can contribute to its development. Here's a breakdown of the causes and risk factors:   - **Aging:** As people age, there's a natural decline in muscle mass and strength. This age-related muscle loss is a primary driver of sarcopenia. - **Physical Inactivity:** Lack of exercise, especially resistance training, is a major contributor. Muscles need to be used to maintain their mass and strength. - **Poor Nutrition:** Inadequate protein and calorie intake can lead to muscle breakdown. Insufficient intake of essential nutrients like vitamin D can also play a role. - **Hormonal Changes:** Age-related decline in hormones like testosterone, growth hormone, and insulin-like growth factor (IGF-1) can affect muscle mass. - **Chronic Diseases:** Certain chronic illnesses, such as cancer, heart failure, chronic obstructive pulmonary disease (COPD), kidney disease, and diabetes, can accelerate muscle loss. - **Inflammation:** Chronic inflammation can contribute to muscle breakdown. - **Other Factors:** Other potential factors include genetics, certain medications (like corticosteroids), and neurological conditions.   To summarize, while aging is the primary risk factor, lifestyle factors (inactivity and poor nutrition) and underlying health conditions significantly influence the development and progression of sarcopenia.  Related searches:   - [causes of sarcopenia](https://www.google.com/search?q=causes+of+sarcopenia&client=app-vertex-grounding-quora-poe)   [risk factors for sarcopenia](https://www.google.com/search?q=risk+factors+for+sarcopenia&client=app-vertex-grounding-quora-poe) | The causes of **sarcopenia** are multifactorial, with the primary driver being aging. However, various interconnected factors contribute to its onset and progression:  **1. Aging**   - Natural decline in muscle mass begins around age 30–40 and accelerates after age 60.   **2. Physical Inactivity**   - Sedentary lifestyle reduces muscle stimulation and promotes muscle atrophy. - Lack of regular resistance training accelerates muscle mass and strength loss.   **3. Poor Nutrition**   - **Protein deficiency:** Low dietary protein intake impairs muscle protein synthesis. - **Vitamin D deficiency:** Crucial for muscle function and strength. - **Caloric insufficiency or malnutrition:** Leads to muscle wasting.   **4. Hormonal Changes**   - Reduction in anabolic hormones with aging, such as:   - **Testosterone** (men and women)   - **Growth hormone**   - **Insulin-like growth factor-1 (IGF-1)**   - **Estrogen** (especially post-menopause in women)   **5. Chronic Inflammation**   - Elevated inflammatory markers (e.g., cytokines like TNF-alpha, IL-6) accelerate muscle breakdown and inhibit muscle synthesis. - Conditions like obesity and chronic inflammatory diseases worsen muscle health.   **6. Neuromuscular Changes**   - Decline in motor neurons leads to decreased muscle stimulation, atrophy, and loss of coordination.   **7. Chronic Diseases and Conditions**   - Diabetes, heart failure, kidney disease, chronic obstructive pulmonary disease (COPD), and arthritis are associated with increased risk of sarcopenia. - Cancer and cancer-related treatments can cause rapid muscle loss (cachexia).   **8. Oxidative Stress**   - Accumulation of oxidative damage in aging muscles disrupts muscle cell function, promoting muscle degradation.   **9. Mitochondrial Dysfunction**   - Aging muscles experience reduced mitochondrial number and efficiency, impairing energy production and contributing to muscle fatigue and atrophy.   **Summary of Key Causes:**   \| **Cause** \| **Example** \| \| --- \| --- \| \| Aging \| Age-related muscle fiber atrophy \| \| Physical inactivity \| Sedentary lifestyle \| \| Nutrition deficiencies \| Protein, vitamin D deficiency \| \| Hormonal changes \| Testosterone, growth hormone decline \| \| Chronic inflammation \| Elevated cytokines (TNF-α, IL-6) \| \| Neuromuscular decline \| Motor neuron loss \| \| Chronic diseases \| Diabetes, heart failure, cancer \| \| Oxidative stress \| Accumulation of reactive oxygen species \| \| Mitochondrial issues \| Reduced energy metabolism \|   Effective management of sarcopenia involves addressing these underlying causes through appropriate exercise, nutritional interventions, medical treatment, and lifestyle modifications. |
| 3 | Can sarcopenia be caused by other diseases or drugs? | Yes, **sarcopenia** (the age-related loss of muscle mass, strength, and function) can be caused or exacerbated by **other diseases** and **drugs**. While aging is the primary factor, certain medical conditions and medications can accelerate muscle loss or impair muscle function. Here’s a detailed look at how diseases and drugs can contribute to sarcopenia: **Diseases That Can Cause or Worsen Sarcopenia**  1. **Chronic Diseases**:    - **Diabetes**: Insulin resistance and high blood sugar levels can impair muscle protein synthesis and lead to muscle wasting.    - **Chronic Kidney Disease (CKD)**: Uremic toxins and inflammation in CKD can accelerate muscle breakdown.    - **Chronic Obstructive Pulmonary Disease (COPD)**: Reduced physical activity and systemic inflammation contribute to muscle loss.    - **Heart Failure**: Poor circulation and reduced physical activity can lead to muscle atrophy.    - **Cancer**: Cancer cachexia (a wasting syndrome) causes severe muscle loss due to inflammation and metabolic changes. 2. **Inflammatory and Autoimmune Diseases**:    - **Rheumatoid Arthritis (RA)**: Chronic inflammation and reduced mobility can lead to muscle wasting.    - **Inflammatory Bowel Disease (IBD)**: Malabsorption of nutrients and chronic inflammation can impair muscle health. 3. **Neurological Disorders**:    - **Parkinson’s Disease**: Reduced mobility and muscle rigidity contribute to muscle loss.    - **Multiple Sclerosis (MS)**: Muscle weakness and inactivity due to neurological damage can lead to sarcopenia.    - **Stroke**: Muscle atrophy often occurs in affected limbs due to reduced use. 4. **Infections**:    - **HIV/AIDS**: Chronic inflammation and metabolic changes can lead to muscle wasting.    - **Chronic Infections**: Persistent infections can cause systemic inflammation and muscle loss. 5. **Endocrine Disorders**:    - **Hypothyroidism**: Low thyroid hormone levels can lead to muscle weakness and atrophy.    - **Cushing’s Syndrome**: Excess cortisol (a stress hormone) can cause muscle breakdown.  **Drugs That Can Cause or Worsen Sarcopenia**  1. **Corticosteroids**:    - **Effect**: Long-term use of steroids (e.g., prednisone) can lead to muscle wasting by increasing protein breakdown and reducing protein synthesis.    - **Conditions Treated**: Asthma, rheumatoid arthritis, lupus, and other inflammatory conditions. 2. **Statins**:    - **Effect**: Statins (cholesterol-lowering drugs) can cause muscle pain, weakness, and, in rare cases, severe muscle damage (rhabdomyolysis).    - **Conditions Treated**: High cholesterol and cardiovascular disease. 3. **Proton Pump Inhibitors (PPIs)**:    - **Effect**: Long-term use of PPIs (e.g., omeprazole) can impair nutrient absorption (e.g., magnesium, vitamin B12), affecting muscle health.    - **Conditions Treated**: Acid reflux and stomach ulcers. 4. **Antidepressants**:    - **Effect**: Some antidepressants (e.g., SSRIs) can cause fatigue, weakness, and reduced physical activity.    - **Conditions Treated**: Depression and anxiety. 5. **Chemotherapy Drugs**:    - **Effect**: Chemotherapy can cause muscle wasting and fatigue due to systemic toxicity and inflammation.    - **Conditions Treated**: Cancer. 6. **Antihistamines**:    - **Effect**: Long-term use of antihistamines (e.g., diphenhydramine) can cause drowsiness and reduced physical activity.    - **Conditions Treated**: Allergies and insomnia. 7. **Diuretics**:    - **Effect**: Diuretics (e.g., furosemide) can cause electrolyte imbalances (e.g., low potassium), leading to muscle weakness.    - **Conditions Treated**: High blood pressure and heart failure.  **How to Mitigate Drug- and Disease-Related Sarcopenia**  1. **Optimize Treatment**:    - Work with your healthcare provider to manage underlying diseases effectively.    - Adjust medications to minimize side effects that contribute to muscle loss. 2. **Nutrition**:    - Ensure adequate protein intake (1.0–1.2 g/kg of body weight per day for older adults).    - Include nutrients like vitamin D, calcium, and omega-3 fatty acids to support muscle health. 3. **Exercise**:    - Engage in resistance training and aerobic exercise to build and maintain muscle mass and strength.    - Physical therapy can help if mobility is limited. 4. **Monitor Muscle Health**:    - Regular assessments of muscle mass, strength, and function can help detect sarcopenia early. 5. **Lifestyle Changes**:    - Avoid smoking and excessive alcohol consumption, which can worsen muscle loss.    - Stay active to counteract the effects of sedentary behavior.  **Summary** Sarcopenia can be caused or worsened by chronic diseases, inflammation, and certain medications. Managing underlying conditions, optimizing drug regimens, and adopting a healthy lifestyle (including proper nutrition and exercise) are key to preventing or mitigating muscle loss. If you’re at risk for sarcopenia, consult your healthcare provider for personalized advice and interventions. | Yes, sarcopenia can be caused or worsened by other diseases and certain drugs.  **Diseases That Can Contribute to Sarcopenia:**   - **Chronic Diseases:** Several chronic conditions are linked to an increased risk of sarcopenia, including:   - Cancer   - Chronic Obstructive Pulmonary Disease (COPD)   - Chronic Kidney Disease   - Heart Failure   - Diabetes   - Rheumatoid Arthritis and other inflammatory conditions   - HIV/AIDS - **Malabsorption Syndromes:** Conditions that interfere with nutrient absorption can lead to muscle loss. - **Neurological Disorders:** Some neurological conditions can affect muscle function and contribute to sarcopenia.   **Drugs That Can Contribute to Sarcopenia:**   - **Corticosteroids (e.g., Prednisone):** These anti-inflammatory drugs can cause muscle breakdown (atrophy) as a side effect, especially with long-term use. - **Statins:** While primarily used to lower cholesterol, some studies have suggested a possible link between statin use and muscle weakness or myopathy in some individuals. - **Chemotherapy Drugs:** Many chemotherapy drugs used to treat cancer can have toxic effects on muscles. - **GLP-1 Agonists:** Drugs like Wegovy and Ozempic, while effective for weight loss, can lead to sarcopenia and frailty due to the loss of lean body mass. - **Other Medications:** Some other medications, such as certain anticonvulsants and immunosuppressants, may also contribute to muscle loss in some cases.   It's important to note that the relationship between specific drugs and sarcopenia can be complex and may vary depending on the individual, dosage, duration of use, and other factors. If you're concerned about potential muscle loss related to a medication you're taking, it's best to consult with your doctor.  Related searches:   - [sarcopenia caused by diseases](https://www.google.com/search?q=sarcopenia+caused+by+diseases&client=app-vertex-grounding-quora-poe)   [sarcopenia caused by drugs](https://www.google.com/search?q=sarcopenia+caused+by+drugs&client=app-vertex-grounding-quora-poe) | Yes, sarcopenia can indeed be caused or accelerated by various diseases and certain medications. These conditions typically exacerbate muscle wasting, inflammation, or hormonal imbalances, leading to secondary sarcopenia.  **Diseases Associated with Sarcopenia:**   1. **Chronic diseases:**    - **Chronic Obstructive Pulmonary Disease (COPD)** Leads to chronic inflammation and reduced physical activity.    - **Heart Failure** Promotes muscle wasting due to reduced exercise tolerance, inflammation, and nutritional deficiencies.    - **Chronic Kidney Disease (CKD)** Associated with metabolic and hormonal disturbances, chronic inflammation, and poor nutrition.    - **Diabetes Mellitus** Impairs muscle metabolism, causing insulin resistance and inflammation-induced muscle wasting. 2. **Cancer (Cancer Cachexia):**    - Cancer and its treatments commonly cause significant muscle loss due to inflammation, anorexia, metabolic alterations, and reduced physical activity. 3. **Neurological disorders:**    - **Stroke**    - **Parkinson's disease**    - **Multiple sclerosis** These conditions reduce mobility and physical activity, contributing to accelerated muscle atrophy. 4. **Inflammatory and autoimmune diseases:**    - **Rheumatoid arthritis**    - **Systemic lupus erythematosus (SLE)** Chronic inflammation and corticosteroid treatment lead to muscle loss. 5. **Endocrine disorders:**    - **Hyperthyroidism:** Accelerates muscle breakdown.    - **Hypogonadism (low testosterone):** Results in decreased muscle mass.    - **Cushing’s syndrome:** Excess cortisol leads to significant muscle wasting. 6. **Liver diseases:**    - **Liver cirrhosis** Promotes protein-energy malnutrition and hormonal disruptions.   **Drugs and Medications Associated with Sarcopenia:**  Certain medications accelerate muscle loss or impair muscle protein synthesis:   1. **Glucocorticoids (Steroids):**    - **Prednisone, dexamethasone, hydrocortisone**    - Cause significant muscle wasting (steroid-induced myopathy). 2. **Chemotherapy agents:**    - **Doxorubicin, cisplatin, cyclophosphamide**    - Induce muscle loss through direct muscle toxicity and anorexia. 3. **Statins:**    - **Atorvastatin, simvastatin, rosuvastatin**    - Rarely associated with muscle pain, weakness, and myopathy. 4. **Androgen deprivation therapy (ADT):**    - **Used for prostate cancer**    - Causes muscle loss due to reduced testosterone levels. 5. **Thyroid medications (in excess doses):**    - Excessive **levothyroxine** may induce hyperthyroid state and accelerate muscle breakdown. 6. **Proton pump inhibitors (PPIs):**    - Long-term use associated with impaired nutrient absorption (especially protein, calcium, vitamin B12), indirectly leading to muscle wasting.   **Summary Table:**   \| **Category** \| **Examples** \| **Mechanism** \| \| --- \| --- \| --- \| \| **Chronic diseases** \| COPD, heart failure, CKD, diabetes \| Inflammation, decreased mobility, hormonal imbalance \| \| **Cancer cachexia** \| Lung, colorectal, pancreatic cancers \| Inflammation, metabolic changes, reduced intake \| \| **Neurological disorders** \| Stroke, Parkinson’s, multiple sclerosis \| Reduced activity, impaired neuromuscular function \| \| **Autoimmune disorders** \| Rheumatoid arthritis, SLE \| Chronic inflammation, steroid use \| \| **Endocrine disorders** \| Hyperthyroidism, hypogonadism, Cushing’s \| Hormonal imbalance, accelerated catabolism \| \| **Liver diseases** \| Cirrhosis \| Nutritional deficit, impaired metabolism \| \| **Medications** \| Steroids, chemotherapy, statins, ADT, PPIs \| Direct muscle toxicity, hormonal suppression, impaired nutrient absorption \|   **Clinical implications:**   - Identifying underlying diseases or medications that cause or exacerbate sarcopenia is essential for effective treatment. - Treatment often requires managing the primary illness or adjusting medications alongside exercise and nutritional strategies.   Addressing secondary causes of sarcopenia improves outcomes and quality of life, emphasizing the need for comprehensive patient evaluation. |
| 4 | Who is prone to sarcopenia? | Sarcopenia, the progressive loss of muscle mass, strength, and function, is most commonly associated with aging but can also affect other populations. Here are the key groups prone to sarcopenia: **1. Older Adults**  - **Primary Group**: Sarcopenia is most prevalent in individuals aged **60 and older**. - **Accelerated Loss**: Muscle mass and strength decline by **3-8% per decade after age 30**, accelerating after age 60. - **Increased Risk**: By age 80, up to **50% of older adults** may experience sarcopenia.  **2. Sedentary Individuals**  - **Lack of Physical Activity**: Inactivity leads to muscle atrophy and accelerates sarcopenia. - **Bed Rest or Immobility**: Prolonged bed rest due to illness or hospitalization can cause rapid muscle loss.  **3. People with Chronic Diseases**  - **Diabetes**: Poor blood sugar control can damage muscles and nerves. - **Heart Disease**: Reduced physical activity and poor circulation contribute to muscle loss. - **Chronic Kidney Disease (CKD)**: Impaired kidney function affects muscle protein synthesis. - **Chronic Obstructive Pulmonary Disease (COPD)**: Reduced physical activity and inflammation contribute to muscle wasting. - **Cancer**: Cancer and its treatments (e.g., chemotherapy) can lead to muscle loss.  **4. Individuals with Poor Nutrition**  - **Protein Deficiency**: Inadequate protein intake impairs muscle maintenance and repair. - **Vitamin D Deficiency**: Low vitamin D levels are linked to muscle weakness. - **Malnutrition**: Poor overall nutrition accelerates muscle loss.  **5. Hormonal Changes**  - **Menopause**: Declining estrogen levels in women can contribute to muscle loss. - **Low Testosterone**: In men, reduced testosterone levels with aging can lead to sarcopenia.  **6. Genetic Factors**  - **Family History**: A genetic predisposition to lower muscle mass or strength may increase risk. - **Ethnicity**: Some studies suggest that certain ethnic groups may be more prone to sarcopenia.  **7. Smokers and Heavy Alcohol Users**  - **Smoking**: Reduces muscle mass and impairs muscle function. - **Alcohol**: Excessive consumption can damage muscles and nerves.  **8. Post-Surgery or Hospitalized Patients**  - **Immobilization**: Prolonged bed rest during recovery leads to muscle atrophy. - **Stress and Inflammation**: Surgery or illness can trigger muscle breakdown.  **9. Individuals with Neurological Disorders**  - **Parkinson’s Disease**: Affects muscle control and leads to weakness. - **Stroke**: Can cause muscle atrophy on the affected side.  **10. People with Low Physical Activity Levels**  - **Sedentary Lifestyle**: Lack of exercise accelerates muscle loss. - **Frailty**: Older adults with frailty are at higher risk of sarcopenia.  **Prevention and Management**  1. **Exercise**:    - **Resistance Training**: Weightlifting or resistance exercises to build muscle.    - **Aerobic Exercise**: Activities like walking or swimming to maintain overall fitness. 2. **Nutrition**:    - **High-Protein Diet**: Include lean meats, fish, eggs, dairy, legumes, and nuts.    - **Vitamin D and Calcium**: Ensure adequate intake for muscle and bone health. 3. **Lifestyle Changes**:    - **Quit Smoking**: Reduces muscle damage.    - **Limit Alcohol**: Prevents muscle and nerve damage. 4. **Medical Management**:    - **Address Chronic Conditions**: Properly manage diseases like diabetes or heart disease.    - **Hormone Therapy**: For individuals with hormonal imbalances (e.g., low testosterone or estrogen).  **Summary** Sarcopenia primarily affects older adults but can also occur in individuals with chronic diseases, poor nutrition, or sedentary lifestyles. Early intervention through exercise, proper nutrition, and lifestyle changes can help prevent or slow the progression of sarcopenia. If you’re at risk, consult a healthcare provider for personalized advice and management strategies. | Here's a breakdown of who is most prone to sarcopenia:   - **Older Adults:** The risk of sarcopenia increases significantly with age. After the age of 60, muscle mass and strength naturally decline, making older individuals the most vulnerable population. - **Individuals with Sedentary Lifestyles:** People who are physically inactive are at a higher risk. Lack of exercise, especially resistance training, accelerates muscle loss. - **People with Poor Nutrition:** Inadequate protein and calorie intake, as well as deficiencies in essential nutrients like vitamin D, increase the risk. - **Individuals with Chronic Diseases:** Certain chronic conditions, such as cancer, COPD, heart failure, kidney disease, diabetes, and inflammatory diseases, can accelerate muscle loss. - **People with Hormonal Imbalances:** Age-related decline in hormones like testosterone, growth hormone, and insulin-like growth factor (IGF-1) can contribute to muscle loss. - **Individuals Taking Certain Medications:** Long-term use of corticosteroids and other medications can increase the risk. - **People with Genetic Predisposition:** Genetics may play a role in determining an individual's susceptibility to sarcopenia. - **Individuals with Obesity:** While it might seem counterintuitive, obesity can sometimes mask sarcopenia (known as sarcopenic obesity), where muscle mass is low but masked by excess fat. - **Individuals with Malabsorption Issues:** Conditions that affect nutrient absorption can increase the risk. - **People with Limited Access to Healthcare:** Lack of access to proper medical care and preventative measures can increase the risk of developing sarcopenia. - **Certain Ethnic Groups:** Some studies suggest that certain ethnic groups, such as Asian populations, may be at higher risk for sarcopenia.   Related searches:  [who is at risk for sarcopenia](https://www.google.com/search?q=who+is+at+risk+for+sarcopenia&client=app-vertex-grounding-quora-poe) | Individuals prone to **sarcopenia** typically share certain characteristics or risk factors. These factors can broadly be categorized into demographic, lifestyle, medical, and nutritional groups.  **🔹 1. Demographic Factors:**   - **Older Adults:**   - Age is the strongest predictor; muscle mass and strength naturally decline after age 40 and accelerate after age 60. - **Gender:**   - Women, particularly post-menopausal, experience accelerated muscle loss due to hormonal changes.   - Older men also experience significant muscle loss linked to declining testosterone levels.   **🔹 2. Lifestyle Factors:**   - **Sedentary Lifestyle:**   - Lack of regular physical activity, especially resistance training or weight-bearing exercises. - **Prolonged Bed Rest or Immobilization:**   - After hospitalization, illness, surgery, or injury, prolonged inactivity rapidly accelerates muscle loss.   **🔹 3. Nutritional Factors:**   - **Protein Insufficiency:**   - Diets low in protein significantly impair muscle protein synthesis. - **Malnutrition or Poor Diet:**   - Inadequate calorie intake, poor diet quality, or chronic undernutrition increase risk. - **Vitamin D Deficiency:**   - Essential for muscle function; deficiency accelerates muscle weakness and wasting.   **🔹 4. Medical Conditions:**   - **Chronic Diseases:**   - Diabetes, COPD, heart failure, chronic kidney disease, arthritis, cancer (especially cachexia). - **Neurological Disorders:**   - Stroke, Parkinson’s disease, multiple sclerosis, and dementia reduce mobility and muscle stimulation. - **Hormonal Disorders:**   - Low testosterone (hypogonadism), estrogen deficiency, growth hormone deficiency, hyperthyroidism, Cushing's syndrome. - **Inflammatory and Autoimmune Diseases:**   - Rheumatoid arthritis, lupus (SLE), inflammatory bowel disease (IBD) increase inflammatory cytokines that degrade muscle.   **🔹 5. Medication Exposure:**   - Chronic or prolonged use of:   - **Glucocorticoids (Steroids)**: prednisone, dexamethasone.   - **Chemotherapy drugs**: for cancer treatment.   - **Hormonal therapy**: androgen deprivation therapy (ADT) for prostate cancer.   - **Statins** (rarely but possible): atorvastatin, simvastatin.   - **Proton pump inhibitors (PPIs)**: reduce nutrient absorption affecting muscle maintenance.   **🔹 6. Social and Psychological Factors:**   - **Social Isolation or Depression:**   - Often results in reduced physical activity and poor dietary habits. - **Low Socioeconomic Status:**   - Limited access to nutritious foods or facilities promoting physical activity.   **✅ Groups Most at Risk (Summary):**   - Elderly (especially age ≥65) - Post-menopausal women - Sedentary individuals - Individuals with chronic illnesses (COPD, diabetes, heart/kidney diseases, cancer) - Patients experiencing long-term immobilization - Malnourished or protein-deficient individuals - Those taking specific medications chronically (steroids, chemotherapy, ADT)   **Practical Implications:**   - Early screening and preventive interventions (nutrition, physical exercise) can significantly reduce risk and severity. - Tailored treatment plans should target modifiable risk factors such as diet, activity, and medication management. |
| 5 | At what age does sarcopenia typically begin? | Sarcopenia, the age-related loss of muscle mass, strength, and function, typically begins around the **age of 30** but becomes more noticeable and clinically significant after **age 50**. The rate of muscle loss accelerates with advancing age, and the condition is most pronounced in older adults. Here’s a detailed breakdown: **Age-Related Progression of Sarcopenia**  1. **30s**:    - Muscle mass begins to decline at a rate of **3-5% per decade**.    - This early stage is often asymptomatic and not yet considered clinically significant. 2. **50s**:    - Muscle loss accelerates, and sarcopenia becomes more noticeable.    - Strength and function may start to decline, impacting daily activities. 3. **60s and Beyond**:    - Muscle mass decreases by **1-2% per year**, and strength declines by **1.5-5% per year**.    - Sarcopenia becomes a significant risk factor for frailty, falls, and loss of independence.  **Factors Influencing Sarcopenia**  1. **Physical Inactivity**: Lack of exercise accelerates muscle loss. 2. **Poor Nutrition**: Inadequate protein intake or malnutrition contributes to muscle wasting. 3. **Chronic Diseases**: Conditions like diabetes, heart disease, or cancer can worsen sarcopenia. 4. **Hormonal Changes**: Declines in hormones like testosterone, estrogen, and growth hormone play a role. 5. **Lifestyle Factors**: Smoking, excessive alcohol use, and poor sleep can exacerbate muscle loss.  **Preventing and Managing Sarcopenia**  1. **Exercise**:    - **Resistance Training**: Lifting weights or using resistance bands helps build and maintain muscle mass.    - **Aerobic Exercise**: Activities like walking, swimming, or cycling improve overall fitness and endurance.    - **Balance and Flexibility Training**: Yoga or tai chi can improve stability and reduce fall risk. 2. **Nutrition**:    - **Protein Intake**: Aim for **1.0-1.2 grams of protein per kilogram of body weight per day** (higher for older adults).    - **Essential Nutrients**: Ensure adequate intake of vitamin D, calcium, and omega-3 fatty acids. 3. **Lifestyle Changes**:    - **Quit Smoking**: Smoking accelerates muscle loss.    - **Limit Alcohol**: Excessive alcohol consumption can impair muscle repair.    - **Sleep Well**: Aim for 7-9 hours of quality sleep per night to support muscle recovery. 4. **Medical Interventions**:    - **Hormone Therapy**: In some cases, hormone replacement therapy may be considered.    - **Medications**: Research is ongoing, but no specific medications are currently approved for sarcopenia.  **When to Seek Help** If you notice significant muscle weakness, difficulty performing daily tasks, or unexplained weight loss, consult a healthcare provider. Early intervention can help slow the progression of sarcopenia and improve quality of life. **Summary** Sarcopenia typically begins around age 30 but becomes more pronounced after age 50. Regular exercise, proper nutrition, and a healthy lifestyle can help prevent or manage this condition. If you’re concerned about muscle loss, talk to your doctor or a physical therapist for personalized recommendations. | Sarcopenia typically begins gradually around the age of 30 or 40, but the rate of muscle loss accelerates significantly after the age of 60 or 65.  Here's a more detailed breakdown:   - **Early Stages (30s-40s):** Muscle mass starts to decline slowly, often unnoticed. The rate of decline is usually around 1-2% per year. - **Middle Age (50s-60s):** The rate of muscle loss may increase slightly. - **Later Stages (60s and beyond):** The rate of muscle loss accelerates, with some studies suggesting a loss of up to 0.5-1% of muscle mass per year. This is when the effects of sarcopenia become more noticeable, leading to weakness, decreased mobility, and increased risk of falls.   It's important to note that the onset and progression of sarcopenia can be influenced by various factors, including genetics, lifestyle, nutrition, and underlying health conditions. Individuals who are physically inactive, have poor nutrition, or have chronic diseases may experience an earlier onset and more rapid progression of sarcopenia. Also, it is important to remember that the rate of muscle loss can vary significantly from person to person.  Related searches:  [at what age does sarcopenia start](https://www.google.com/search?q=at+what+age+does+sarcopenia+start&client=app-vertex-grounding-quora-poe) | Sarcopenia typically begins in adulthood, with gradual muscle loss starting around the age of **30 to 40 years**. However, the rate of muscle decline remains relatively slow initially and significantly accelerates after approximately **60 years of age**.  **📌 Typical progression timeline:**   - **Ages 30–40:**   - Gradual decline begins, with muscle mass decreasing by about **0.5–1% per year**. - **Ages 50–60:**   - Noticeable changes may begin, especially in sedentary individuals. Muscle strength declines faster than muscle mass. - **Ages 60 and older:**   - Muscle mass and strength loss accelerate markedly (approximately **1–2% per year**, with strength loss even faster at **2–3% per year**).   - Functional impairments, reduced mobility, increased fall risk, and reduced quality of life become increasingly apparent.   **📌 Factors influencing earlier or faster onset:**   - Sedentary lifestyle or physical inactivity - Poor nutrition (especially insufficient protein intake) - Chronic illness (diabetes, COPD, heart disease, cancer) - Hormonal changes (especially menopause in women, low testosterone in men) - Long-term use of medications (e.g., corticosteroids)   **📌 Clinical significance:**  Early intervention (ideally before the age of 50–60) through:   - **Regular resistance training** - **Adequate protein and nutritional intake** - **Addressing underlying medical issues**   can significantly delay or reduce the progression and impact of sarcopenia.  **Summary:**   - **Early adulthood (30–40 years):** Onset of gradual muscle loss begins. - **60+ years:** Rapid acceleration and clinical relevance become significant. |
| **Topic: Risk factors** | |  |  |  |
| 6 | What are the risk factors for developing sarcopenia? | **Sarcopenia**, the age-related loss of muscle mass, strength, and function, is influenced by a combination of biological, lifestyle, and environmental factors. Here are the primary **risk factors** for developing sarcopenia: **1. Age**  - **Primary Risk Factor**: Sarcopenia is most common in individuals aged **50 and older**, with muscle mass declining by **3-8% per decade** after age 30. - **Accelerated Loss**: Muscle loss speeds up after age 60 and becomes more pronounced after age 80.  **2. Physical Inactivity**  - **Sedentary Lifestyle**: Lack of exercise accelerates muscle loss. - **Bed Rest or Immobility**: Prolonged inactivity due to illness or hospitalization can lead to rapid muscle wasting.  **3. Poor Nutrition**  - **Inadequate Protein Intake**: Protein is essential for muscle repair and growth; insufficient intake accelerates muscle loss. - **Caloric Deficiency**: Undereating, especially in older adults, can lead to muscle wasting. - **Vitamin D Deficiency**: Low vitamin D levels are linked to muscle weakness and increased fall risk.  **4. Chronic Diseases**  - **Diabetes**: Poor blood sugar control can damage muscles and nerves. - **Heart Disease**: Reduced physical activity and poor circulation contribute to muscle loss. - **Chronic Kidney Disease (CKD)**: Impaired kidney function affects muscle protein synthesis. - **Chronic Obstructive Pulmonary Disease (COPD)**: Reduced physical activity and inflammation contribute to muscle wasting. - **Cancer**: Cancer and its treatments (e.g., chemotherapy) can lead to muscle loss.  **5. Hormonal Changes**  - **Menopause**: Declining estrogen levels in women can contribute to muscle loss. - **Low Testosterone**: In men, reduced testosterone levels with aging can lead to sarcopenia. - **Growth Hormone Deficiency**: Impairs muscle repair and growth.  **6. Neurological Disorders**  - **Parkinson’s Disease**: Affects muscle control and leads to weakness. - **Stroke**: Can cause muscle atrophy on the affected side. - **Multiple Sclerosis (MS)**: Muscle weakness and inactivity due to neurological damage can lead to sarcopenia.  **7. Inflammation**  - **Chronic Low-Grade Inflammation**: Aging is associated with increased levels of inflammatory markers like cytokines, which can break down muscle tissue.  **8. Medications**  - **Corticosteroids**: Long-term use can lead to muscle wasting. - **Statins**: Can cause muscle pain and weakness in some individuals. - **Other Drugs**: Some medications for chronic conditions may have side effects that contribute to muscle loss.  **9. Lifestyle Factors**  - **Smoking**: Reduces muscle mass and impairs muscle function. - **Excessive Alcohol Consumption**: Can damage muscle tissue and impair nutrient absorption. - **Poor Sleep**: Inadequate sleep can impair muscle recovery and growth.  **10. Genetic Factors**  - **Family History**: A genetic predisposition to lower muscle mass or strength may increase risk. - **Ethnicity**: Some studies suggest that certain ethnic groups may be more prone to sarcopenia.  **11. Social and Environmental Factors**  - **Social Isolation**: Lack of social engagement can lead to reduced physical activity and poor nutrition. - **Limited Access to Healthcare**: Delayed diagnosis or treatment of contributing conditions.  **Prevention and Management**  1. **Exercise**:    - **Resistance Training**: Weightlifting or resistance band exercises to build muscle.    - **Aerobic Exercise**: Walking, swimming, or cycling to improve overall fitness.    - **Balance Training**: Yoga or tai chi to reduce fall risk. 2. **Nutrition**:    - **High-Protein Diet**: Include lean meats, fish, eggs, dairy, legumes, and nuts.    - **Essential Nutrients**: Ensure adequate intake of vitamin D, calcium, and omega-3 fatty acids. 3. **Lifestyle Changes**:    - **Quit Smoking**: Reduces muscle damage.    - **Limit Alcohol**: Prevents muscle and nerve damage.    - **Sleep Well**: Aim for 7-9 hours of quality sleep per night to support muscle recovery. 4. **Medical Management**:    - **Address Chronic Conditions**: Properly manage diseases like diabetes or heart disease.    - **Hormone Therapy**: For individuals with hormonal imbalances (e.g., low testosterone or estrogen).  **Summary** The risk factors for sarcopenia include aging, physical inactivity, poor nutrition, chronic diseases, hormonal changes, and lifestyle habits. By addressing these factors through exercise, proper nutrition, and medical management, you can reduce your risk of developing sarcopenia and maintain muscle strength and function as you age. If you’re at risk, consult a healthcare provider for personalized advice and interventions. | Here's a breakdown of the risk factors for developing sarcopenia:   - **Age:** Advanced age is the most significant risk factor. Muscle mass and strength naturally decline with age, especially after 60. - **Physical Inactivity:** A sedentary lifestyle or lack of exercise, particularly resistance training, greatly increases the risk. Muscles need to be used to maintain their mass and strength. - **Poor Nutrition:** Inadequate intake of protein, calories, and essential nutrients like vitamin D can lead to muscle breakdown and hinder muscle protein synthesis. - **Chronic Diseases:** Certain chronic conditions are strongly associated with sarcopenia, including:   - Cancer   - Chronic Obstructive Pulmonary Disease (COPD)   - Chronic Kidney Disease   - Heart Failure   - Diabetes   - Inflammatory conditions like rheumatoid arthritis - **Hormonal Changes:** Age-related declines in hormones like testosterone, growth hormone, and insulin-like growth factor (IGF-1) can contribute to muscle loss. - **Inflammation:** Chronic low-grade inflammation can promote muscle breakdown. - **Medications:** Long-term use of certain medications, such as corticosteroids, can increase the risk. - **Genetics:** Genetic factors may influence an individual's predisposition to sarcopenia. - **Obesity:** While seemingly counterintuitive, obesity can mask sarcopenia (sarcopenic obesity), where muscle mass is low but hidden by excess fat. Obesity can also contribute to inflammation and insulin resistance, further exacerbating muscle loss. - **Smoking:** Smoking can impair muscle protein synthesis and increase the risk of sarcopenia. - **Alcohol Abuse:** Excessive alcohol consumption can interfere with nutrient absorption and muscle metabolism. - **Malabsorption:** Conditions that impair nutrient absorption can contribute to muscle loss. - **Neurological Conditions:** Some neurological disorders can affect muscle function and increase the risk of sarcopenia. - **Socioeconomic Factors:** Limited access to healthcare, nutritious food, and safe environments for physical activity can increase the risk. - **Ethnicity:** Some studies suggest that certain ethnic groups may have a higher risk of sarcopenia. - **Vitamin D Deficiency:** Low levels of vitamin D are associated with muscle weakness and an increased risk of falls, contributing to sarcopenia. - **Depression:** Depression can lead to reduced physical activity and poor appetite, increasing the risk of muscle loss.   Related searches:  [risk factors for sarcopenia](https://www.google.com/search?q=risk+factors+for+sarcopenia&client=app-vertex-grounding-quora-poe) | The risk factors for developing **sarcopenia** include a combination of demographic, lifestyle, medical, nutritional, and medication-related factors.  **📝 Summary of Risk Factors:**   \| **Category** \| **Risk Factors** \| \| --- \| --- \| \| **Demographic** \| - **Advanced age (≥60 years)** - Female gender (post-menopausal women) \| \| **Lifestyle** \| - Sedentary lifestyle - Physical inactivity or low physical activity level - Prolonged bed rest or immobility \| \| **Nutritional** \| - Protein deficiency or low protein intake - Vitamin D deficiency - Malnutrition or calorie insufficiency \| \| **Medical conditions** \| - Chronic diseases (COPD, heart failure, diabetes, CKD, cancer) - Neurological disorders (stroke, Parkinson’s disease, multiple sclerosis) - Inflammatory and autoimmune conditions (rheumatoid arthritis, lupus, inflammatory bowel disease) - Hormonal disorders (low testosterone, menopause, hyperthyroidism, growth hormone deficiency) \| \| **Medications** \| - Glucocorticoids (steroids) - Chemotherapy - Androgen deprivation therapy (ADT) - Long-term proton pump inhibitors (PPIs) - Statins (rarely associated) \| \| **Psychosocial factors** \| - Depression or anxiety - Social isolation - Lower socioeconomic status \|   **✅ Most Commonly Recognized Risk Factors:**   1. **Advanced Age**    - **Primary factor**; risk significantly rises after age **60**. 2. **Physical Inactivity**    - Reduced physical activity dramatically increases the risk. 3. **Poor Nutrition**    - Inadequate dietary intake of protein, vitamin D, and calories accelerates muscle loss. 4. **Chronic Diseases**    - Conditions causing inflammation, reduced mobility, or hormonal imbalance are strong contributors. 5. **Medication Exposure**    - Long-term use of steroids, chemotherapy, ADT, or PPIs can accelerate muscle wasting.   **🔍 Clinical Importance:**   - Recognizing these risk factors early can lead to effective preventative strategies such as improved nutrition, increased physical activity (especially resistance training), and careful management of chronic diseases and medications. - Early interventions significantly improve outcomes, reducing disability, frailty, and enhancing overall quality of life. |
| 7 | Is sarcopenia related to malnutrition or a sedentary lifestyle? | Yes, **sarcopenia** is closely related to both **malnutrition** and a **sedentary lifestyle**. These factors can significantly accelerate the loss of muscle mass, strength, and function, especially in older adults. Here’s how malnutrition and inactivity contribute to sarcopenia: **1. Malnutrition and Sarcopenia** Malnutrition, particularly **protein-energy malnutrition**, plays a major role in the development of sarcopenia. Key aspects include: **a. Inadequate Protein Intake**  - **Role of Protein**: Protein is essential for muscle repair, maintenance, and growth. - **Impact**: Insufficient protein intake reduces the body’s ability to synthesize muscle proteins, leading to muscle wasting. - **Recommendation**: Older adults should aim for **1.0–1.2 grams of protein per kilogram of body weight per day** (higher for those with acute illness or injury).  **b. Caloric Deficiency**  - **Energy Needs**: The body requires sufficient calories to maintain muscle mass and function. - **Impact**: Undereating, especially in older adults, can lead to muscle breakdown as the body uses muscle tissue for energy. - **Solution**: A balanced diet with adequate calories and nutrients is essential.  **c. Micronutrient Deficiencies**  - **Vitamin D**: Low vitamin D levels are linked to muscle weakness and increased fall risk. - **Calcium**: Important for muscle contraction and bone health. - **Omega-3 Fatty Acids**: Reduce inflammation and support muscle protein synthesis. - **Impact**: Deficiencies in these nutrients can worsen sarcopenia.  **d. Malabsorption**  - **Conditions**: Diseases like celiac disease, inflammatory bowel disease (IBD), or chronic pancreatitis can impair nutrient absorption. - **Impact**: Reduced absorption of protein and other nutrients accelerates muscle loss.  **2. Sedentary Lifestyle and Sarcopenia** Physical inactivity is one of the **most significant risk factors** for sarcopenia. Here’s how it contributes: **a. Lack of Muscle Use**  - **Muscle Atrophy**: Muscles that are not used regularly begin to shrink and weaken. - **Impact**: Prolonged inactivity, such as bed rest or a sedentary lifestyle, leads to rapid muscle loss.  **b. Reduced Muscle Protein Synthesis**  - **Exercise Stimulus**: Physical activity, especially resistance training, stimulates muscle protein synthesis. - **Impact**: Without exercise, the body’s ability to build and maintain muscle is impaired.  **c. Loss of Strength and Function**  - **Functional Decline**: Inactivity leads to reduced muscle strength, balance, and coordination, increasing the risk of falls and frailty.  **d. Metabolic Changes**  - **Insulin Resistance**: Sedentary behavior can lead to insulin resistance, which impairs muscle protein synthesis. - **Inflammation**: Lack of exercise is associated with chronic low-grade inflammation, which can break down muscle tissue.  **How to Address Malnutrition and Inactivity**  1. **Nutrition**:    - **Increase Protein Intake**: Include high-quality protein sources like lean meats, fish, eggs, dairy, legumes, and nuts.    - **Balanced Diet**: Ensure adequate intake of calories, vitamins, and minerals.    - **Supplements**: Consider protein supplements or vitamin D if dietary intake is insufficient. 2. **Exercise**:    - **Resistance Training**: Weightlifting or resistance band exercises 2–3 times per week to build muscle.    - **Aerobic Exercise**: Walking, swimming, or cycling to improve overall fitness.    - **Balance Training**: Yoga or tai chi to reduce fall risk. 3. **Lifestyle Changes**:    - **Stay Active**: Avoid prolonged sitting; incorporate movement into daily routines.    - **Monitor Health**: Address chronic conditions that may contribute to malnutrition or inactivity. 4. **Medical Support**:    - **Dietitian Consultation**: For personalized nutrition advice.    - **Physical Therapy**: For tailored exercise programs, especially if mobility is limited.  **Summary** Sarcopenia is strongly linked to **malnutrition** (especially protein deficiency) and a **sedentary lifestyle**. Addressing these factors through proper nutrition, regular exercise, and lifestyle changes can help prevent or slow the progression of sarcopenia. If you’re at risk, consult a healthcare provider for personalized recommendations. | Yes, sarcopenia is strongly related to both malnutrition and a sedentary lifestyle. They are both significant contributing factors to its development and progression.  **Malnutrition:**   - **Protein Deficiency:** Inadequate protein intake is a major driver of sarcopenia. Protein is essential for muscle protein synthesis, the process by which the body builds and repairs muscle tissue. When protein intake is insufficient, the body may break down muscle to obtain the amino acids it needs for other vital functions. - **Calorie Deficiency:** Insufficient calorie intake can also lead to muscle loss. When the body doesn't get enough calories, it may break down muscle tissue for energy. - **Vitamin and Mineral Deficiencies:** Deficiencies in certain vitamins and minerals, such as vitamin D, vitamin B12, and creatine, can also contribute to muscle weakness and loss. - **Reduced Appetite:** Conditions that reduce appetite or make it difficult to eat enough can lead to malnutrition and sarcopenia. - **Poor Nutrient Absorption:** Conditions that affect nutrient absorption, such as celiac disease or Crohn's disease, can lead to malnutrition and increase the risk of sarcopenia.   **Sedentary Lifestyle:**   - **Lack of Muscle Use:** Muscles need to be used regularly to maintain their mass and strength. A sedentary lifestyle leads to muscle disuse, which triggers muscle breakdown and reduces muscle protein synthesis. - **Reduced Physical Activity:** Physical activity, especially resistance training, is crucial for stimulating muscle growth and preventing muscle loss. A sedentary lifestyle deprives muscles of this stimulus. - **Increased Risk of Other Health Problems:** A sedentary lifestyle increases the risk of obesity, insulin resistance, and other health problems that can further contribute to sarcopenia. - **Impaired Muscle Metabolism:** Lack of physical activity can impair muscle metabolism and reduce the efficiency of muscle function.   In summary, both malnutrition and a sedentary lifestyle are significant risk factors for sarcopenia. Malnutrition deprives the body of the building blocks and energy it needs to maintain muscle mass, while a sedentary lifestyle deprives muscles of the stimulus they need to stay strong. Addressing both of these factors is crucial for preventing and managing sarcopenia.  Related searches:   - [sarcopenia malnutrition](https://www.google.com/search?q=sarcopenia+malnutrition&client=app-vertex-grounding-quora-poe)   [sarcopenia sedentary lifestyle](https://www.google.com/search?q=sarcopenia+sedentary+lifestyle&client=app-vertex-grounding-quora-poe) | Yes, **sarcopenia** is strongly related to both **malnutrition** and a **sedentary lifestyle**. These two factors significantly increase the risk, accelerate progression, and worsen the severity of sarcopenia.  Here's how these relationships work:  **📌 1. Sarcopenia and Malnutrition:**  Malnutrition contributes directly to sarcopenia through several mechanisms:   - **Protein deficiency:**   - Adequate protein is essential for maintaining muscle mass and strength.   - Insufficient protein intake impairs muscle protein synthesis, accelerating muscle loss. - **Energy (caloric) deficiency:**   - Insufficient caloric intake forces the body to break down muscle tissue for energy.   - Chronic calorie restriction or inadequate nutrition significantly accelerates muscle wasting. - **Micronutrient deficiencies:**   - **Vitamin D deficiency** impairs muscle function and strength.   - **Calcium, vitamin B12, antioxidants, omega-3 fatty acids, and essential amino acids deficiencies** all negatively impact muscle maintenance and strength. - **Chronic disease-related malnutrition:**   - Conditions like cancer, kidney disease, or gastrointestinal diseases cause malnutrition and further accelerate sarcopenia.   **📌 2. Sarcopenia and a Sedentary Lifestyle:**  Physical inactivity is a major driver of sarcopenia:   - **Lack of muscle stimulation:**   - Muscles require regular exercise—especially resistance or weight-bearing exercise—to maintain mass and strength.   - Inactivity rapidly accelerates muscle atrophy, especially in older adults. - **Reduced neuromuscular function:**   - Sedentary behavior weakens the neuromuscular connection, impairing muscle coordination and strength. - **Increased inflammation and metabolic dysfunction:**   - Sedentary behavior increases chronic inflammation and insulin resistance, further exacerbating muscle breakdown. - **Prolonged bed rest or immobility:**   - Even short periods (days or weeks) of inactivity due to hospitalization or illness can significantly accelerate muscle loss.   **✅ Relationship Summary:**   \| **Factor** \| **Mechanism of Influence** \| **Impact on Sarcopenia** \| \| --- \| --- \| --- \| \| **Malnutrition** \| Protein/calorie deficiency; micronutrient deficits \| Direct acceleration of muscle loss \| \| **Sedentary lifestyle** \| Reduced muscle stimulation; inflammation; muscle atrophy \| Rapid muscle deterioration \|   **⚠️ Clinical Implications:**   - Improving **nutrition** (protein-rich diets, vitamin D supplementation, balanced caloric intake) and increasing **physical activity** (regular resistance training, aerobic exercises, mobility exercises) are key strategies to prevent and manage sarcopenia effectively. - Addressing these two factors simultaneously offers substantial potential for preventing and slowing the progression of sarcopenia, significantly enhancing older adults' health, functionality, and quality of life. |
| 8 | Can medication use for weight loss in obesity cause sarcopenia? | Yes, **medication use for weight loss in obesity** can potentially contribute to **sarcopenia** (the loss of muscle mass, strength, and function) if not managed carefully. While weight loss is often beneficial for overall health, rapid or excessive weight loss can lead to muscle wasting, especially if the weight loss is not accompanied by proper nutrition and exercise. Here’s how weight-loss medications may increase the risk of sarcopenia and what you can do to mitigate it: **How Weight-Loss Medications Can Contribute to Sarcopenia**  1. **Rapid Weight Loss**:    - **Muscle Loss**: Rapid weight loss often results in the loss of both fat and muscle mass.    - **Impact**: Medications that cause significant calorie restriction or reduced appetite can lead to muscle breakdown if protein intake is insufficient. 2. **Reduced Protein Intake**:    - **Appetite Suppressants**: Some weight-loss medications reduce appetite, which may lead to inadequate protein consumption.    - **Impact**: Insufficient protein intake impairs muscle repair and growth, accelerating sarcopenia. 3. **Nutrient Malabsorption**:    - **Medications Like Orlistat**: Orlistat reduces fat absorption, which can also impair the absorption of fat-soluble vitamins (e.g., vitamin D) essential for muscle health.    - **Impact**: Nutrient deficiencies can worsen muscle loss and weakness. 4. **Metabolic Changes**:    - **GLP-1 Receptor Agonists (e.g., Semaglutide, Liraglutide)**: These medications can lead to weight loss by reducing appetite and slowing gastric emptying, but they may also cause muscle loss if not combined with adequate protein intake and resistance training.    - **Impact**: Without proper nutrition and exercise, weight loss may disproportionately affect muscle mass.  **How to Prevent Sarcopenia While Using Weight-Loss Medications**  1. **Ensure Adequate Protein Intake**:    - **Daily Protein Goal**: Aim for **1.0–1.2 grams of protein per kilogram of body weight per day** (higher for older adults or those with significant muscle loss).    - **Protein Sources**: Include lean meats, fish, eggs, dairy, legumes, and plant-based proteins like tofu or tempeh. 2. **Engage in Resistance Training**:    - **Exercise**: Perform resistance training (e.g., weightlifting, resistance bands) at least **2–3 times per week** to preserve and build muscle mass.    - **Physical Therapy**: If needed, work with a physical therapist to develop a safe and effective exercise program. 3. **Monitor Nutrient Intake**:    - **Vitamin D and Calcium**: Ensure adequate intake to support muscle and bone health.    - **Balanced Diet**: Eat a variety of nutrient-dense foods to prevent deficiencies. 4. **Avoid Rapid Weight Loss**:    - **Gradual Weight Loss**: Aim for a slow, steady weight loss of **0.5–1 kg (1–2 lbs) per week** to minimize muscle loss.    - **Medical Supervision**: Work with a healthcare provider to monitor your weight loss and adjust medications as needed. 5. **Supplementation**:    - **Protein Supplements**: If dietary protein intake is insufficient, consider protein shakes or bars.    - **Multivitamins**: Use supplements to address potential nutrient deficiencies, especially if taking medications that affect nutrient absorption. 6. **Regular Monitoring**:    - **Muscle Mass Assessment**: Use tools like bioelectrical impedance analysis (BIA) or dual-energy X-ray absorptiometry (DEXA) to monitor muscle mass.    - **Strength and Function Tests**: Assess grip strength, walking speed, or chair stand tests to track muscle function.  **Common Weight-Loss Medications and Their Risks**  1. **Orlistat (Xenical, Alli)**:    - Reduces fat absorption but may impair absorption of fat-soluble vitamins (e.g., vitamin D). 2. **GLP-1 Receptor Agonists (e.g., Semaglutide, Liraglutide)**:    - Effective for weight loss but may cause muscle loss if not combined with proper nutrition and exercise. 3. **Phentermine-Topiramate (Qsymia)**:    - Suppresses appetite but may lead to inadequate protein intake if not monitored. 4. **Bupropion-Naltrexone (Contrave)**:    - Reduces appetite but may require dietary adjustments to ensure adequate protein.  **Summary** Weight-loss medications can contribute to sarcopenia if they lead to rapid weight loss, inadequate protein intake, or nutrient deficiencies. To minimize this risk, focus on **adequate protein intake**, **regular resistance training**, and **gradual weight loss** under medical supervision. If you’re using weight-loss medications, consult your healthcare provider or a dietitian to develop a plan that preserves muscle mass while achieving your weight-loss goals. | Yes, medication use for weight loss in obesity can potentially cause sarcopenia.  Here's a more detailed explanation:   - **GLP-1 Receptor Agonists:** Medications like semaglutide (Ozempic, Wegovy) and liraglutide (Saxenda) are GLP-1 receptor agonists that promote weight loss by reducing appetite and increasing satiety. While effective for weight loss, studies have shown that a significant portion of the weight lost can be lean muscle mass. This is concerning because the loss of muscle mass can contribute to sarcopenia and frailty. - **Other Weight Loss Medications:** Other weight loss medications that suppress appetite or reduce nutrient absorption can also potentially lead to muscle loss if not combined with adequate protein intake and resistance exercise. - **Rapid Weight Loss:** Regardless of the method, rapid weight loss can increase the risk of muscle loss. When the body is in a calorie deficit, it may break down both fat and muscle for energy.   To mitigate the risk of sarcopenia during weight loss with medications, it's crucial to:   - **Maintain Adequate Protein Intake:** Consuming enough protein is essential to preserve muscle mass during weight loss. - **Engage in Resistance Exercise:** Resistance training helps stimulate muscle protein synthesis and prevent muscle loss. - **Monitor Body Composition:** Regularly monitoring body composition (muscle mass and fat mass) can help track changes and adjust the weight loss strategy accordingly. - **Work with a Healthcare Professional:** A healthcare professional can help develop a safe and effective weight loss plan that minimizes muscle loss and promotes overall health.   In summary, while weight loss medications can be effective for treating obesity, they can also potentially contribute to sarcopenia if not used carefully and in conjunction with strategies to preserve muscle mass.  Related searches:   - [how to prevent muscle loss during weight loss](https://www.google.com/search?q=how+to+prevent+muscle+loss+during+weight+loss&client=app-vertex-grounding-quora-poe) - [weight loss medications cause sarcopenia](https://www.google.com/search?q=weight+loss+medications+cause+sarcopenia&client=app-vertex-grounding-quora-poe)   [GLP-1 agonists muscle loss](https://www.google.com/search?q=GLP-1+agonists+muscle+loss&client=app-vertex-grounding-quora-poe) | Yes, certain medications used for **weight loss in obesity** can potentially contribute to the development or worsening of **sarcopenia**. Although the primary aim of weight-loss medications is fat reduction, unintended loss of muscle mass can occur, particularly if weight loss is rapid or nutrition and exercise are not properly managed.  Here's how this relationship can occur:  **📝 Mechanisms linking weight-loss medications and sarcopenia:**  **1. Rapid Weight Loss:**   - Rapid weight loss typically leads to loss of **both fat and muscle mass**, especially if adequate dietary protein and physical activity are not maintained. - Muscle loss can be disproportionate if weight loss is accelerated by medication.   **2. Reduced Nutrient Absorption (Malabsorptive Drugs):**   - Certain medications or surgical treatments (e.g., Orlistat, bariatric surgery) reduce absorption of fat and sometimes essential nutrients (protein, vitamins D, B12, calcium). - Long-term use or misuse may lead to nutritional deficiencies, contributing to muscle loss.   **3. Appetite Suppression:**   - Medications that significantly suppress appetite (e.g., **GLP-1 receptor agonists** like semaglutide, liraglutide; **phentermine/topiramate**) may lead to reduced protein and calorie intake. - Insufficient dietary intake results in muscle catabolism and loss of lean muscle tissue.   **4. Reduced Physical Activity:**   - Some weight-loss drugs can cause fatigue or weakness, indirectly decreasing physical activity, thereby accelerating muscle loss.   **💊 Common Weight-Loss Medications and Sarcopenia Risk:**   \| **Medication class or type** \| **Examples** \| **Risk of Muscle Loss** \| \| --- \| --- \| --- \| \| **Lipase inhibitors** (fat malabsorption) \| Orlistat \| Moderate (indirect via nutrient deficiencies) \| \| **GLP-1 receptor agonists** \| Semaglutide, liraglutide \| Moderate (due to appetite suppression, caloric restriction) \| \| **Sympathomimetic agents** (appetite suppressants) \| Phentermine \| Moderate (due to appetite suppression, possible reduced intake) \| \| **Combination agents** \| Phentermine/topiramate \| Moderate–High (due to rapid weight loss, appetite suppression) \|   **Note:** The risk of muscle loss increases substantially if dietary protein intake is inadequate and if weight loss is rapid without resistance exercise.  **⚠️ Clinical Recommendations to Minimize Risk:**   - **Balanced protein intake:** Ensure adequate protein to preserve muscle mass. - **Regular resistance training:** Include strength exercises to maintain muscle mass. - **Monitor nutritional status:** Regularly assess for nutritional deficiencies and supplement vitamins/minerals as necessary. - **Gradual, controlled weight loss:** Aim for slower, sustained weight loss rather than rapid reductions.   **✅ Summary:**  Yes, weight-loss medications used in obesity can contribute to sarcopenia if muscle mass and nutrition are not carefully managed. The risk is greatest with rapid weight loss, inadequate protein/nutrient intake, and lack of exercise. Proper medical supervision and lifestyle interventions can effectively reduce this risk. |
| 9 | Can sarcopenia be associated with osteoporosis? | Yes, **sarcopenia** (the loss of muscle mass, strength, and function) is often associated with **osteoporosis** (the loss of bone density and increased fracture risk). These two conditions frequently coexist, particularly in older adults, and share several common risk factors and underlying mechanisms. This combination is sometimes referred to as **"osteosarcopenia,"** a condition that significantly increases the risk of frailty, falls, and fractures. **How Sarcopenia and Osteoporosis Are Linked**  1. **Shared Risk Factors**:    - **Aging**: Both conditions are more common in older adults.    - **Physical Inactivity**: Lack of exercise contributes to muscle and bone loss.    - **Poor Nutrition**: Inadequate intake of protein, calcium, and vitamin D affects both muscle and bone health.    - **Hormonal Changes**: Declines in estrogen (in postmenopausal women) and testosterone (in men) can lead to both muscle and bone loss.    - **Chronic Inflammation**: Low-grade inflammation associated with aging can accelerate the breakdown of muscle and bone tissue. 2. **Muscle-Bone Interaction**:    - **Mechanical Loading**: Muscles exert force on bones during physical activity, which stimulates bone formation. Reduced muscle mass and strength decrease this stimulus, leading to bone loss.    - **Frailty and Falls**: Weak muscles increase the risk of falls, which can lead to fractures, especially in individuals with osteoporosis. 3. **Common Pathways**:    - **Vitamin D Deficiency**: Low vitamin D levels impair both muscle function and bone health.    - **Insulin Resistance**: Associated with reduced muscle mass and bone density.    - **Chronic Diseases**: Conditions like diabetes, heart disease, and kidney disease can contribute to both sarcopenia and osteoporosis.  **Consequences of Osteosarcopenia**  1. **Increased Fracture Risk**:    - Weak bones (osteoporosis) are more prone to fractures, and weak muscles (sarcopenia) increase the likelihood of falls. 2. **Reduced Mobility and Independence**:    - Combined muscle and bone loss can lead to difficulty performing daily activities and a loss of independence. 3. **Higher Mortality Risk**:    - The combination of sarcopenia and osteoporosis is associated with increased morbidity and mortality.  **Prevention and Management**  1. **Exercise**:    - **Resistance Training**: Weightlifting or resistance band exercises to build muscle and stimulate bone formation.    - **Weight-Bearing Exercise**: Activities like walking, jogging, or dancing to strengthen bones.    - **Balance Training**: Yoga or tai chi to reduce fall risk. 2. **Nutrition**:    - **Protein Intake**: Aim for **1.0–1.2 grams of protein per kilogram of body weight per day** (higher for older adults).    - **Calcium and Vitamin D**: Ensure adequate intake to support bone health (e.g., dairy, leafy greens, fortified foods, supplements if needed).    - **Balanced Diet**: Include a variety of nutrient-dense foods to support overall health. 3. **Lifestyle Changes**:    - **Quit Smoking**: Smoking accelerates bone and muscle loss.    - **Limit Alcohol**: Excessive alcohol consumption can impair bone and muscle health.    - **Stay Active**: Avoid prolonged sitting and incorporate movement into daily routines. 4. **Medical Management**:    - **Bone-Strengthening Medications**: Bisphosphonates, denosumab, or other osteoporosis treatments if prescribed.    - **Hormone Therapy**: For individuals with hormonal deficiencies (e.g., testosterone or estrogen replacement).    - **Fall Prevention**: Address vision, hearing, and balance issues, and make home modifications to reduce fall risk. 5. **Monitoring**:    - **Bone Density Tests**: Regular DEXA scans to monitor bone health.    - **Muscle Mass Assessment**: Use tools like bioelectrical impedance analysis (BIA) or dual-energy X-ray absorptiometry (DEXA) to track muscle mass.  **Summary** Sarcopenia and osteoporosis are closely linked, often coexisting in older adults and increasing the risk of fractures, frailty, and loss of independence. Addressing both conditions through **exercise**, **proper nutrition**, **lifestyle changes**, and **medical management** can help reduce these risks and improve overall health. If you’re at risk for osteosarcopenia, consult a healthcare provider for personalized advice and interventions. | Yes, sarcopenia and osteoporosis are often associated with each other. They are both age-related conditions that can occur independently but frequently coexist, leading to a greater risk of adverse health outcomes. The combination of sarcopenia and osteoporosis is sometimes referred to as "osteosarcopenia."  Here's why they are related:   - **Shared Risk Factors:** Both conditions share several risk factors, including:   - Advanced age   - Physical inactivity   - Poor nutrition (inadequate protein, calcium, and vitamin D intake)   - Hormonal changes (e.g., decreased estrogen in women, decreased testosterone in men)   - Chronic inflammation - **Interdependence of Muscle and Bone:** Muscle and bone are metabolically interconnected. Muscle contractions exert mechanical stress on bones, which stimulates bone formation and remodeling. Conversely, bone provides structural support for muscles. When muscle mass and strength decline (sarcopenia), the mechanical loading on bones decreases, leading to bone loss (osteoporosis). - **Increased Risk of Falls:** Sarcopenia leads to decreased muscle strength and impaired balance, increasing the risk of falls. Osteoporosis weakens bones, making them more susceptible to fractures during falls. Therefore, the combination of sarcopenia and osteoporosis significantly elevates the risk of fractures. - **Common Pathophysiological Mechanisms:** Both conditions share some common underlying mechanisms, such as:   - Inflammation   - Oxidative stress   - Hormonal imbalances - **Vitamin D Deficiency:** Vitamin D plays a crucial role in both muscle and bone health. Deficiency in vitamin D is a common factor contributing to both sarcopenia and osteoporosis.   Because of the close relationship between sarcopenia and osteoporosis, it's important to assess and manage both conditions in older adults to reduce the risk of falls, fractures, and other adverse health outcomes. Management strategies often include:   - **Exercise:** Weight-bearing and resistance exercises are beneficial for both muscle and bone health. - **Nutrition:** Adequate protein, calcium, and vitamin D intake are essential. - **Medications:** Medications may be used to treat osteoporosis and, in some cases, to address muscle loss.   Related searches:   - [sarcopenia and osteoporosis](https://www.google.com/search?q=sarcopenia+and+osteoporosis&client=app-vertex-grounding-quora-poe)   [osteosarcopenia](https://www.google.com/search?q=osteosarcopenia&client=app-vertex-grounding-quora-poe) | Yes, **sarcopenia** is strongly associated with **osteoporosis**. Both conditions commonly occur together, particularly in older adults, and share several overlapping risk factors and underlying mechanisms.  **🔗 Relationship between Sarcopenia and Osteoporosis:**  **1. Common Pathophysiology (Shared Mechanisms):**   - **Aging:** Both muscle and bone mass naturally decline with age. - **Hormonal Changes:** Decreased hormones (testosterone, estrogen, growth hormone) affect both muscle and bone health. - **Chronic Inflammation:** Elevated inflammatory markers (e.g., cytokines such as IL-6, TNF-α) accelerate loss of both muscle and bone tissue. - **Vitamin D and Calcium Deficiency:** Crucial nutrients needed for both muscle function and bone mineralization. - **Reduced Physical Activity:** Leads to muscle weakness (sarcopenia) and bone loss (osteoporosis).   **2. Muscle-Bone Interaction:**   - Muscles exert mechanical forces on bones, stimulating bone formation and maintaining bone density. - Loss of muscle mass and strength reduces mechanical loading on bones, leading to increased bone resorption and decreased bone density.   **3. Increased Risk of Falls and Fractures:**   - Sarcopenia causes muscle weakness and impaired balance, significantly increasing the risk of falls. - Osteoporosis weakens bones, making them prone to fracture after even minor falls. - Individuals with both conditions have significantly elevated fracture risk and compromised quality of life.   **📌 Clinical Terms for Combined Conditions:**   - **Osteosarcopenia:** A term frequently used in clinical practice and research to describe the coexistence of osteoporosis and sarcopenia. It highlights the shared risks and combined impact on health outcomes.   **✅ Clinical Implications:**   - **Early detection and intervention** are critical, including combined assessment for muscle (sarcopenia) and bone (osteoporosis) health. - **Interventions beneficial for both conditions** include:   - **Resistance training and weight-bearing exercises**   - **Adequate dietary protein, calcium, and vitamin D intake**   - **Managing underlying chronic diseases and inflammation**   **📊 Summary Table:**   \| **Feature** \| **Sarcopenia** \| **Osteoporosis** \| **Shared Risk Factors** \| \| --- \| --- \| --- \| --- \| \| Primary Affected Tissue \| Skeletal muscle \| Bone \| Aging, inactivity, malnutrition \| \| Primary Deficit \| Muscle mass and strength \| Bone mineral density (BMD) \| Hormonal decline, inflammation \| \| Consequences \| Weakness, falls, disability \| Fractures, falls, reduced mobility \| Falls, reduced physical function \|   **📝 Conclusion:**  Sarcopenia and osteoporosis often coexist, share common causes, and synergistically increase risks of falls, fractures, disability, and decreased quality of life. Addressing both conditions simultaneously through appropriate lifestyle and therapeutic interventions is vital for optimal patient care. |
| 10 | Can sarcopenia be associated with obesity? | Yes, **sarcopenia** (the loss of muscle mass, strength, and function) can be associated with **obesity**, a condition referred to as **sarcopenic obesity**. This combination is particularly concerning because it involves both excess body fat and reduced muscle mass, leading to a higher risk of metabolic, functional, and health complications. Here’s how sarcopenia and obesity are linked and why this combination is dangerous: **What is Sarcopenic Obesity?** Sarcopenic obesity is characterized by:   - **Low Muscle Mass**: Reduced skeletal muscle mass and strength. - **High Body Fat**: Excess adipose tissue, typically defined by a high body mass index (BMI) or waist circumference.  **How Sarcopenia and Obesity Are Linked**  1. **Shared Risk Factors**:    - **Aging**: Both sarcopenia and obesity are more common in older adults.    - **Sedentary Lifestyle**: Physical inactivity contributes to muscle loss and fat accumulation.    - **Poor Nutrition**: Diets high in calories but low in protein can lead to both fat gain and muscle loss.    - **Chronic Inflammation**: Obesity is associated with low-grade inflammation, which can accelerate muscle breakdown. 2. **Metabolic Dysfunction**:    - **Insulin Resistance**: Obesity often leads to insulin resistance, which impairs muscle protein synthesis and promotes muscle loss.    - **Hormonal Imbalances**: Excess fat tissue can disrupt hormones like leptin and adiponectin, affecting muscle metabolism. 3. **Reduced Physical Function**:    - **Limited Mobility**: Excess body weight combined with weak muscles makes it harder to move and perform daily activities.    - **Increased Fatigue**: Obesity and sarcopenia can lead to fatigue, further reducing physical activity levels.  **Health Risks of Sarcopenic Obesity**  1. **Increased Mortality Risk**:    - The combination of obesity and sarcopenia is associated with a higher risk of death compared to either condition alone. 2. **Metabolic Syndrome**:    - Higher risk of developing conditions like type 2 diabetes, high blood pressure, and dyslipidemia. 3. **Cardiovascular Disease**:    - Increased risk of heart disease and stroke due to poor metabolic health. 4. **Functional Decline**:    - Reduced ability to perform daily activities, leading to loss of independence. 5. **Higher Fall Risk**:    - Weak muscles and excess body weight increase the likelihood of falls and fractures.  **Causes of Sarcopenic Obesity**  1. **Aging**:    - Natural decline in muscle mass and strength, combined with a tendency to gain fat with age. 2. **Physical Inactivity**:    - Lack of exercise accelerates muscle loss and fat gain. 3. **Poor Diet**:    - High-calorie, low-protein diets contribute to fat accumulation and muscle wasting. 4. **Chronic Diseases**:    - Conditions like diabetes, heart disease, or kidney disease can worsen both sarcopenia and obesity. 5. **Hormonal Changes**:    - Declines in hormones like testosterone, estrogen, and growth hormone can exacerbate muscle loss and fat gain.  **Prevention and Management**  1. **Exercise**:    - **Resistance Training**: Weightlifting or resistance band exercises to build muscle.    - **Aerobic Exercise**: Activities like walking, swimming, or cycling to burn fat and improve cardiovascular health.    - **Combination Training**: A mix of resistance and aerobic exercise is most effective for addressing both sarcopenia and obesity. 2. **Nutrition**:    - **High-Protein Diet**: Aim for **1.0–1.2 grams of protein per kilogram of body weight per day** (higher for older adults).    - **Balanced Diet**: Focus on nutrient-dense, low-calorie foods to reduce fat while preserving muscle.    - **Caloric Deficit**: Gradual weight loss through a modest caloric deficit to minimize muscle loss. 3. **Lifestyle Changes**:    - **Stay Active**: Avoid prolonged sitting and incorporate movement into daily routines.    - **Sleep Well**: Aim for 7–9 hours of quality sleep per night to support muscle repair and metabolism.    - **Stress Management**: Reduce stress, which can contribute to fat gain and muscle loss. 4. **Medical Management**:    - **Address Chronic Conditions**: Properly manage diseases like diabetes or heart disease.    - **Hormone Therapy**: For individuals with hormonal imbalances (e.g., low testosterone or estrogen). 5. **Monitoring**:    - **Body Composition Analysis**: Use tools like bioelectrical impedance analysis (BIA) or dual-energy X-ray absorptiometry (DEXA) to track muscle mass and fat percentage.    - **Functional Tests**: Assess strength, balance, and mobility to guide interventions.  **Summary** Sarcopenia and obesity often coexist, particularly in older adults, leading to **sarcopenic obesity**. This combination increases the risk of metabolic, functional, and health complications. Addressing both conditions through **exercise**, **proper nutrition**, **lifestyle changes**, and **medical management** is essential to reduce risks and improve overall health. If you’re at risk for sarcopenic obesity, consult a healthcare provider for personalized advice and interventions. | Yes, sarcopenia can indeed be associated with obesity. This combination is often referred to as "sarcopenic obesity."  Here's how the two conditions can coexist and interact:   - **Definition of Sarcopenic Obesity:** Sarcopenic obesity is characterized by the presence of both low muscle mass and strength (sarcopenia) along with excess body fat (obesity). It's a condition where an individual may have a normal or even high body weight due to the excess fat, but their muscle mass is significantly reduced. - **How They Interact:**   - **Inflammation:** Obesity is associated with chronic low-grade inflammation, which can promote muscle breakdown and inhibit muscle protein synthesis, contributing to sarcopenia.   - **Insulin Resistance:** Obesity often leads to insulin resistance, which can impair muscle protein synthesis and contribute to muscle loss.   - **Physical Inactivity:** Both obesity and sarcopenia can contribute to a cycle of physical inactivity. Obesity can make it more difficult to exercise, while sarcopenia reduces muscle strength and endurance, making physical activity more challenging.   - **Dietary Factors:** Poor dietary habits, such as high intake of processed foods and low intake of protein, can contribute to both obesity and sarcopenia. - **Health Risks:** Sarcopenic obesity is associated with a greater risk of several adverse health outcomes compared to either condition alone, including:   - Increased risk of metabolic syndrome   - Increased risk of cardiovascular disease   - Increased risk of type 2 diabetes   - Increased risk of functional decline and disability   - Increased risk of mortality - **Diagnosis:** Diagnosing sarcopenic obesity can be challenging because individuals may have a normal or high BMI due to the excess fat mass. Therefore, it's important to assess both muscle mass and body composition using techniques such as:   - Dual-energy X-ray absorptiometry (DXA)   - Bioelectrical impedance analysis (BIA)   - Computed tomography (CT) or magnetic resonance imaging (MRI) - **Management:** Management of sarcopenic obesity typically involves a combination of strategies:   - **Lifestyle Modifications:**     - **Diet:** A balanced diet with adequate protein intake is crucial.     - **Exercise:** Resistance training is essential to build muscle mass, and aerobic exercise can help reduce body fat.   - **Medical Management:** In some cases, medical interventions may be necessary to address underlying metabolic issues or hormonal imbalances. - **Hormonal Imbalances:** Obesity can disrupt hormonal balance, including lower testosterone levels, which can contribute to muscle loss. - **Reduced Mobility:** Obesity can lead to reduced mobility and physical activity, which in turn can exacerbate muscle loss and sarcopenia.   In summary, sarcopenia and obesity can coexist, leading to a condition called sarcopenic obesity. This combination is associated with increased health risks and requires a comprehensive management approach that includes lifestyle modifications and, in some cases, medical interventions.  Related searches:   - [sarcopenic obesity](https://www.google.com/search?q=sarcopenic+obesity&client=app-vertex-grounding-quora-poe)   [sarcopenia and obesity](https://www.google.com/search?q=sarcopenia+and+obesity&client=app-vertex-grounding-quora-poe) | Yes, **sarcopenia** can indeed be associated with **obesity**, a condition referred to as **sarcopenic obesity**. This combination is particularly concerning due to its compounded negative effects on health and functional capacity.  **📌 What is Sarcopenic Obesity?**   - Sarcopenic obesity describes the coexistence of **reduced muscle mass and strength (sarcopenia)** with **excessive fat accumulation (obesity)**. - Individuals with sarcopenic obesity have increased fat mass, especially visceral fat, coupled with significant reductions in muscle mass and strength.   **🔄 Why Sarcopenia and Obesity Occur Together:**  **1. Physical Inactivity:**   - Obesity often leads to sedentary lifestyles, promoting muscle atrophy and increased fat accumulation simultaneously.   **2. Inflammation (Chronic Low-grade Inflammation):**   - Excessive adipose (fat) tissue secretes inflammatory cytokines (e.g., TNF-α, IL-6), accelerating muscle loss and impairing muscle function.   **3. Insulin Resistance:**   - Obesity-associated insulin resistance impairs muscle protein synthesis, promoting muscle loss and reducing muscle quality.   **4. Hormonal Imbalances:**   - Increased fat mass disrupts hormone regulation, leading to decreased anabolic hormones (testosterone, growth hormone), further reducing muscle mass and strength.   **5. Poor Nutritional Quality:**   - Diets high in calories but low in essential nutrients (especially protein) contribute to muscle loss even amidst obesity.   **🚩 Health Implications of Sarcopenic Obesity:**   - **Increased Risk of Disability:** Reduced muscle mass and increased fat lead to impaired mobility and physical function. - **Higher Risk of Metabolic Diseases:** Greater incidence of diabetes, hypertension, and cardiovascular disease. - **Elevated Fall and Fracture Risk:** Muscle weakness combined with higher body weight significantly increases the risk of falls and fractures. - **Reduced Quality of Life and Independence:** Difficulty performing daily tasks and reduced functional independence.   **✅ Clinical Recommendations for Managing Sarcopenic Obesity:**   - **Exercise:** Combination of **resistance training** (to improve muscle mass and strength) and **aerobic exercise** (for fat reduction). - **Balanced Nutrition:** Adequate protein intake to maintain muscle mass, alongside controlled calorie restriction for gradual weight loss. - **Address Underlying Conditions:** Control inflammation, insulin resistance, and hormonal imbalances. - **Medical Supervision:** Tailored interventions to manage both muscle and fat loss carefully, avoiding rapid weight loss that could accelerate muscle loss.   **📊 Summary of Sarcopenia and Obesity Interaction:**   \| **Feature** \| **Sarcopenia** \| **Obesity** \| **Sarcopenic Obesity Impact** \| \| --- \| --- \| --- \| --- \| \| Primary Problem \| Muscle loss, weakness \| Excessive fat accumulation \| Reduced muscle strength/function combined with excess fat \| \| Underlying Mechanisms \| Aging, inactivity, inflammation \| Sedentary lifestyle, inflammation, insulin resistance \| Increased inflammation, accelerated muscle loss, metabolic dysfunction \| \| Consequences \| Falls, fractures, reduced function \| Cardiovascular disease, diabetes \| Higher risk of disability, chronic diseases, and poor health outcomes \|   **📝 Conclusion:**  Sarcopenia is frequently associated with obesity, forming a distinct clinical entity (**sarcopenic obesity**) characterized by compounded health risks. Proper diagnosis, management strategies combining physical activity and nutrition, and individualized clinical care can significantly improve outcomes in individuals affected by this challenging condition. |
| **Topic: Clinical presentation** | | | | |
| 11 | What are the early signs and symptoms of sarcopenia? | (First search) Sarcopenia is the progressive loss of muscle mass, strength, and function that occurs with aging or due to other factors such as inactivity, chronic illness, or poor nutrition. Recognizing the early signs and symptoms is important for timely intervention. Here are the key early indicators:  **1. Reduced Muscle Strength**   - Difficulty lifting objects that were previously manageable. - Struggling with daily tasks like opening jars, carrying groceries, or standing up from a chair.   **2. Decreased Physical Performance**   - Slower walking speed or reduced mobility. - Feeling fatigued more easily during routine activities. - Trouble climbing stairs or getting out of a chair without assistance.   **3. Loss of Muscle Mass**   - Noticeable thinning or shrinking of muscles, particularly in the arms, legs, or thighs. - Clothes fitting more loosely due to muscle loss.   **4. Balance and Coordination Issues**   - Increased unsteadiness or difficulty maintaining balance. - Higher risk of falls or near-falls.   **5. Weight Loss Without Trying**   - Unintentional weight loss, particularly if it involves muscle loss rather than fat.   **6. General Weakness**   - Feeling weaker overall, even without physical exertion. - Reduced endurance during physical activities.   **7. Reduced Physical Activity**   - Decreased interest or ability to engage in exercise or physical activities. - Spending more time sedentary.   **8. Poor Grip Strength**   - Difficulty holding onto objects or a noticeable decline in handshake strength.   **Risk Factors for Sarcopenia**   - Aging (common in individuals over 50). - Sedentary lifestyle or prolonged inactivity. - Poor nutrition, especially inadequate protein intake. - Chronic diseases (e.g., diabetes, heart disease, cancer). - Hormonal changes (e.g., reduced testosterone or growth hormone levels).   **Importance of Early Detection**  Early identification of sarcopenia allows for interventions such as resistance training, dietary adjustments (increased protein intake), and lifestyle changes to slow or reverse muscle loss. If you notice these signs, consult a healthcare professional for evaluation and management.  (second search)  **Sarcopenia**, the age-related loss of muscle mass, strength, and function, often develops gradually, and its early signs and symptoms can be subtle. Recognizing these early indicators is crucial for timely intervention to slow progression and maintain muscle health. Here are the **early signs and symptoms** of sarcopenia: **1. Muscle Weakness**  - **Difficulty Lifting Objects**: Struggling to lift or carry items that were once manageable. - **Reduced Grip Strength**: Difficulty opening jars, holding objects, or performing tasks that require hand strength.  **2. Fatigue**  - **Increased Tiredness**: Feeling more tired than usual during physical activities. - **Reduced Stamina**: Needing more frequent rest breaks during tasks.  **3. Reduced Physical Performance**  - **Slower Walking Speed**: Walking at a slower pace than usual. - **Difficulty Climbing Stairs**: Feeling unsteady or needing to hold onto railings when climbing stairs. - **Trouble Standing Up**: Difficulty rising from a chair or getting out of bed without assistance.  **4. Loss of Muscle Mass**  - **Thinner Arms or Legs**: Noticing a decrease in muscle size, particularly in the arms, legs, or thighs. - **Loose Clothing**: Clothes may feel looser around the arms or legs due to muscle loss.  **5. Balance Issues**  - **Unsteadiness**: Feeling less stable when standing or walking. - **Increased Fall Risk**: Tripping or stumbling more frequently.  **6. Weight Loss**  - **Unintentional Weight Loss**: Losing weight without trying, which may include muscle loss.  **7. Reduced Endurance**  - **Difficulty with Daily Activities**: Finding it harder to perform routine tasks like carrying groceries, cleaning, or gardening. - **Shortness of Breath**: Feeling winded more easily during physical activities.  **8. Changes in Posture**  - **Hunched Posture**: Developing a more stooped or hunched posture due to weakened muscles.  **9. Joint Pain**  - **Increased Joint Discomfort**: Weak muscles can place more strain on joints, leading to pain or discomfort.  **10. Emotional and Psychological Changes**  - **Reduced Confidence**: Feeling less confident about physical abilities. - **Social Withdrawal**: Avoiding activities or social events due to physical limitations.  **When to Seek Help** If you notice any of these early signs of sarcopenia, especially if they interfere with daily life, consult a healthcare provider. Early diagnosis and intervention can help slow the progression of muscle loss and improve quality of life. **How to Address Early Sarcopenia**  1. **Exercise**:    - **Resistance Training**: Weightlifting or resistance band exercises to build muscle.    - **Aerobic Exercise**: Walking, swimming, or cycling to improve overall fitness.    - **Balance Training**: Yoga or tai chi to reduce fall risk. 2. **Nutrition**:    - **High-Protein Diet**: Aim for **1.0–1.2 grams of protein per kilogram of body weight per day** (higher for older adults).    - **Essential Nutrients**: Ensure adequate intake of vitamin D, calcium, and omega-3 fatty acids. 3. **Lifestyle Changes**:    - **Stay Active**: Avoid prolonged sitting and incorporate movement into daily routines.    - **Quit Smoking**: Smoking accelerates muscle loss.    - **Limit Alcohol**: Excessive alcohol consumption can impair muscle repair. 4. **Medical Support**:    - **Bone Density Testing**: To assess for osteoporosis, which often coexists with sarcopenia.    - **Hormone Therapy**: For individuals with hormonal imbalances (e.g., low testosterone or estrogen).   Summary  The early signs of sarcopenia include muscle weakness, fatigue, reduced physical performance, balance issues, and unintentional weight loss. Recognizing these symptoms early allows for timely intervention through **exercise**, **proper nutrition**, and **lifestyle changes**. If you’re concerned about sarcopenia, consult a healthcare provider for personalized advice and management strategies. | The early signs and symptoms of sarcopenia can be subtle and often go unnoticed, as muscle loss typically occurs gradually. Here are some of the key indicators to watch out for:   - **Decreased Strength:**   - Difficulty lifting or carrying objects that were previously manageable.   - Weakened grip strength (e.g., trouble opening jars or turning doorknobs).   - Feeling weaker overall. - **Reduced Stamina and Increased Fatigue:**   - Feeling more tired than usual after normal activities.   - Decreased endurance during physical activities like walking or climbing stairs. - **Slowed Walking Speed:**   - Noticeably slower pace when walking.   - Taking longer to cross the street or complete errands. - **Difficulty with Physical Tasks:**   - Trouble getting up from a chair or bed.   - Difficulty climbing stairs.   - Problems with balance and coordination. - **Unintentional Weight Loss:**   - Losing weight without trying, particularly if it's accompanied by a decrease in muscle mass. - **Decreased Activity Levels:**   - A general decline in overall physical activity and a tendency to be more sedentary. - **Loss of Balance:**   - Increased risk of falls due to impaired balance and coordination. - **Changes in Body Composition:**   - Noticeable loss of muscle mass, particularly in the limbs.   - Clothes fitting differently, especially looser around the arms and legs. - **Decreased Bone Density:**   - Although not a direct symptom of sarcopenia, decreased bone density (osteopenia or osteoporosis) often coexists and can contribute to frailty and increased risk of fractures. - **Reduced Overall Function:**   - A general decline in the ability to perform daily activities independently. - **Increased Frailty:**   - Feeling more vulnerable and less resilient to stressors. - **Mood Changes:**   - Depression or anxiety can sometimes be associated with sarcopenia, as reduced physical function can impact mental well-being.   It's important to note that these signs and symptoms can also be associated with other medical conditions, so it's essential to consult with a healthcare professional for proper evaluation and diagnosis. Early detection and intervention are crucial for managing sarcopenia and preventing further muscle loss and functional decline.  Related searches:   - [early signs of sarcopenia](https://www.google.com/search?q=early+signs+of+sarcopenia&client=app-vertex-grounding-quora-poe)   [symptoms of sarcopenia](https://www.google.com/search?q=symptoms+of+sarcopenia&client=app-vertex-grounding-quora-poe) | The early signs and symptoms of **sarcopenia** may initially be subtle and easily overlooked. Recognizing them promptly can significantly aid in early intervention and prevention of more severe consequences.  **📌 Early Signs and Symptoms:**  **1. Reduced Muscle Strength**   - Difficulty opening jars or lifting previously manageable objects. - Decreased grip strength (often one of the first measurable indicators).   **2. Loss of Muscle Mass (Atrophy)**   - Noticeable reduction in muscle size, especially in arms, legs, thighs, or calves. - Loose or sagging skin around muscle areas due to underlying muscle loss.   **3. Increased Fatigue**   - Feeling tired or experiencing muscle fatigue quickly after routine activities or mild physical tasks.   **4. Reduced Physical Performance**   - Difficulty climbing stairs, getting up from chairs, or maintaining balance. - Slower walking speed, shorter strides, or reduced stamina.   **5. Impaired Balance and Coordination**   - Increased clumsiness, stumbling, or difficulty maintaining stability, particularly during physical activities or daily tasks.   **6. Reduced Functional Independence**   - Needing assistance or modifications to perform daily tasks previously managed independently (e.g., bathing, dressing, household chores).   **7. Unintentional Weight Loss**   - Losing weight without intentionally dieting, particularly muscle-related weight.   **8. Increased Risk or Frequency of Falls**   - Experiencing near-falls or actual falls, especially in older individuals.   **🚩 Summary Table of Early Signs and Symptoms:**   \| **Signs and Symptoms** \| **Examples or Observations** \| \| --- \| --- \| \| **Weakness/Reduced Strength** \| Difficulty lifting objects, reduced grip strength. \| \| **Muscle Loss** \| Thinning arms/legs, loss of muscle tone. \| \| **Fatigue** \| Easily tired during simple activities. \| \| **Functional Decline** \| Difficulty standing up, climbing stairs, slower gait. \| \| **Balance Problems** \| Increased clumsiness, unsteadiness. \| \| **Weight Loss** \| Unexpected, especially muscle mass loss. \| \| **Falls or Near-Falls** \| Frequent stumbling or balance issues. \|   **⚠️ When to Seek Medical Attention:**  If these early symptoms are recognized, consulting a healthcare professional is crucial to confirm diagnosis and implement preventive measures, such as nutritional interventions, resistance training, and lifestyle modifications.  Early identification and intervention significantly slow progression and reduce the severity of sarcopenia. |
| 12 | What are late signs and symptoms of sarcopenia? | (First search) Late-stage sarcopenia is characterized by severe muscle loss, significant functional decline, and increased vulnerability to complications. At this stage, the condition can profoundly impact quality of life and independence. Here are the late signs and symptoms:  **1. Severe Muscle Atrophy**   - Noticeable and significant loss of muscle mass, particularly in the arms, legs, and core. - Extremely thin or frail appearance.   **2. Profound Weakness**   - Extreme difficulty performing basic activities of daily living (e.g., dressing, bathing, eating). - Inability to lift even light objects or stand without assistance.   **3. Severe Mobility Issues**   - Inability to walk or move independently. - Dependence on assistive devices (e.g., walker, wheelchair) or caregivers for mobility.   **4. Frequent Falls and Fractures**   - Increased susceptibility to falls due to poor balance and muscle weakness. - Higher risk of fractures, especially hip fractures, due to osteoporosis and reduced muscle support.   **5. Loss of Independence**   - Inability to live independently and requiring assistance for most daily activities. - Need for long-term care or institutionalization.   **6. Chronic Fatigue**   - Persistent exhaustion, even with minimal physical activity. - Reduced ability to engage in any form of exercise or movement.   **7. Malnutrition and Weight Loss**   - Severe unintentional weight loss due to muscle wasting. - Increased risk of malnutrition, as reduced muscle mass can lead to decreased appetite and difficulty eating.   **8. Poor Posture and Balance**   - Hunched or stooped posture due to weakened core muscles. - Severe balance issues, making standing or walking without assistance nearly impossible.   **9. Increased Risk of Complications**   - Higher susceptibility to infections, pressure sores, and other health issues due to immobility and frailty. - Worsening of chronic conditions (e.g., heart disease, diabetes) due to reduced physical resilience.   **10. Psychological Impact**   - Depression, anxiety, or feelings of hopelessness due to loss of independence and physical function. - Social isolation and reduced quality of life.   **Importance of Managing Late-Stage Sarcopenia**  While late-stage sarcopenia is challenging to reverse, interventions can still improve quality of life and prevent complications. These may include:   - Physical therapy to maintain mobility and strength. - Nutritional support to address malnutrition and promote muscle health. - Assistive devices to enhance independence and safety. - Pain management and psychological support to address emotional well-being.   If you or a loved one is experiencing these symptoms, consult a healthcare professional for a comprehensive care plan tailored to individual needs.  (Second search)  **Late-stage sarcopenia** is characterized by significant muscle loss, severe weakness, and functional decline, which can greatly impact independence and quality of life. Recognizing these **late signs and symptoms** is crucial for managing the condition and preventing further complications. Here’s what to look for:  1. Severe Muscle Weakness   - **Difficulty with Basic Tasks**: Struggling to perform simple activities like lifting a cup, brushing hair, or opening a door. - **Loss of Grip Strength**: Inability to hold objects firmly or perform tasks requiring hand strength.   2. Profound Functional Decline   - **Inability to Walk**: Difficulty walking even short distances without assistance. - **Dependence on Mobility Aids**: Needing a walker, cane, or wheelchair to move around. - **Trouble Standing**: Unable to stand up from a chair or bed without help.   3. Extreme Fatigue   - **Constant Tiredness**: Feeling exhausted even after minimal physical activity. - **Reduced Stamina**: Unable to complete daily tasks without frequent rest breaks.   4. Severe Loss of Muscle Mass   - **Visible Muscle Wasting**: Noticeable thinning of muscles in the arms, legs, or thighs. - **Sunken Appearance**: A hollow or gaunt appearance in the face, arms, or legs.   5. Frequent Falls   - **High Fall Risk**: Increased likelihood of falls due to muscle weakness and poor balance. - **Fractures**: Falls may lead to fractures, particularly in individuals with coexisting osteoporosis.   6. Weight Loss and Malnutrition   - **Severe Weight Loss**: Significant unintentional weight loss, often due to muscle wasting. - **Malnutrition**: Poor appetite or difficulty eating, leading to nutrient deficiencies.   7. Reduced Independence   - **Dependence on Others**: Needing help with daily activities like dressing, bathing, or eating. - **Inability to Live Alone**: Requiring assistance or moving to a care facility.   8. Poor Posture and Mobility   - **Severe Hunched Posture**: A pronounced stooped or hunched posture due to weakened muscles. - **Limited Range of Motion**: Difficulty moving joints fully due to muscle stiffness or weakness.   9. Emotional and Psychological Impact   - **Depression and Anxiety**: Feelings of sadness, hopelessness, or anxiety due to loss of independence. - **Social Isolation**: Withdrawing from social activities due to physical limitations.   10. Increased Risk of Complications   - **Infections**: Weakened immune function due to poor nutrition and muscle loss. - **Chronic Pain**: Joint or muscle pain from reduced mobility and muscle atrophy. - **Higher Mortality Risk**: Severe sarcopenia is associated with increased morbidity and mortality.   How to Manage Late-Stage Sarcopenia   1. **Medical Support**:    - **Physical Therapy**: A tailored program to improve strength, balance, and mobility.    - **Occupational Therapy**: Training to adapt to daily activities and maintain independence.    - **Nutritional Support**: A dietitian can recommend a high-protein, nutrient-dense diet or supplements. 2. **Exercise**:    - **Gentle Resistance Training**: Light weights or resistance bands to maintain muscle mass.    - **Assisted Exercises**: Exercises with the help of a therapist or caregiver. 3. **Nutrition**:    - **Protein-Rich Diet**: Focus on lean meats, fish, eggs, dairy, and plant-based proteins.    - **Caloric Intake**: Ensure adequate calories to prevent further weight loss.    - **Supplements**: Protein shakes or vitamin D supplements if dietary intake is insufficient. 4. **Fall Prevention**:    - **Home Modifications**: Install grab bars, remove tripping hazards, and improve lighting.    - **Assistive Devices**: Use canes, walkers, or wheelchairs as needed. 5. **Psychological Support**:    - **Counseling**: Therapy to address depression, anxiety, or social isolation.    - **Support Groups**: Connecting with others experiencing similar challenges.   Summary  Late-stage sarcopenia is marked by severe muscle weakness, functional decline, frequent falls, and loss of independence. While the condition is challenging, **exercise**, **proper nutrition**, **medical support**, and **fall prevention strategies** can help manage symptoms and improve quality of life. If you or a loved one is experiencing late-stage sarcopenia, consult a healthcare provider for personalized care and interventions. | Late-stage sarcopenia presents with more pronounced and debilitating signs and symptoms, indicating significant muscle loss and functional decline. Here are some of the key indicators:   - **Severe Muscle Weakness:**   - Inability to lift even light objects.   - Difficulty performing basic activities like standing, walking, or dressing.   - Significant loss of strength in all muscle groups. - **Significant Functional Impairment:**   - Dependence on others for assistance with daily tasks (e.g., bathing, dressing, eating).   - Inability to live independently.   - Loss of ability to perform instrumental activities of daily living (IADLs) such as cooking, cleaning, and managing finances. - **Extreme Fatigue:**   - Persistent and overwhelming fatigue that is not relieved by rest.   - Inability to participate in even minimal physical activity.   - Constant feeling of exhaustion. - **Mobility Issues:**   - Severe difficulty walking or maintaining balance.   - Frequent falls and injuries.   - Reliance on assistive devices such as walkers or wheelchairs.   - Bedridden state in severe cases. - **Marked Weight Loss and Muscle Atrophy:**   - Visible and significant loss of muscle mass, particularly in the limbs.   - Emaciated appearance.   - Hollowed facial features. - **Increased Risk of Complications:**   - Higher susceptibility to infections, pressure ulcers, and other complications due to immobility and weakened immune function.   - Increased risk of hospitalization. - **Cognitive Decline:**   - In some cases, severe sarcopenia can be associated with cognitive impairment and reduced mental function.   - Increased risk of dementia. - **Increased Mortality Risk:**   - Late-stage sarcopenia is associated with a significantly increased risk of death.   - Reduced life expectancy. - **Osteoporosis and Fractures:**   - Increased risk of fractures due to weakened bones and increased risk of falls.   - Hip fractures, vertebral fractures, and other serious injuries. - **Respiratory Problems:**   - Weakness of respiratory muscles can lead to difficulty breathing and increased risk of respiratory infections.   - Pneumonia and other lung complications. - **Dysphagia (Difficulty Swallowing):**   - Weakness of the muscles involved in swallowing can lead to difficulty eating and drinking, increasing the risk of malnutrition and aspiration pneumonia.   - Choking episodes. - **Malnutrition and Dehydration:**   - Reduced appetite and difficulty eating can lead to malnutrition and dehydration, further exacerbating muscle loss and weakness.   - Vitamin and mineral deficiencies.   At this stage, the focus of care often shifts to managing symptoms, preventing complications, and improving quality of life. While some interventions may still be helpful, the potential for significant muscle recovery is often limited. Palliative care and end-of-life planning may become necessary.  Related searches:   - [late stage sarcopenia](https://www.google.com/search?q=late+stage+sarcopenia&client=app-vertex-grounding-quora-poe)   [symptoms of advanced sarcopenia](https://www.google.com/search?q=symptoms+of+advanced+sarcopenia&client=app-vertex-grounding-quora-poe) | Late-stage **sarcopenia** symptoms become more pronounced and significantly impact daily life, independence, and overall health. Recognizing these late-stage symptoms is important for effective management and maintaining quality of life.  **📌 Late Signs and Symptoms of Sarcopenia:**  **1. Severe Muscle Weakness**   - Significant difficulty lifting everyday objects (even lightweight items). - Extreme difficulty performing tasks like opening doors, jars, or carrying groceries.   **2. Marked Muscle Atrophy**   - Noticeably reduced muscle size and mass. - Prominent bony appearance (arms, legs) due to severe muscle loss.   **3. Severe Functional Impairment**   - Unable or extremely slow to stand from a chair without assistance. - Difficulty walking, climbing stairs, or rising from a seated or lying position without help.   **4. Frequent Falls**   - Increased occurrence of falls, often leading to injuries, bruises, or fractures. - Reduced ability to regain balance once unsteady.   **5. Loss of Independence**   - Requiring assistance for basic activities of daily living (ADLs), including dressing, bathing, toileting, and feeding. - Dependence on caregivers, aids (walkers, wheelchairs), or home adaptations.   **6. Chronic Fatigue and Low Endurance**   - Persistent exhaustion, even from minimal physical exertion. - Significant decline in energy levels affecting daily routines.   **7. Mobility Limitations**   - Severe restrictions in mobility, with reduced walking speed and distance. - Increased reliance on assistive devices (canes, walkers, wheelchairs).   **8. Increased Risk of Serious Complications**   - Higher risk of osteoporosis and fractures. - Increased susceptibility to chronic illnesses (cardiovascular disease, diabetes). - Frequent hospitalizations related to falls, fractures, or complications from immobility (e.g., pneumonia, pressure ulcers).   **9. Psychological Consequences**   - Depression, anxiety, social isolation, or loss of confidence due to decreased independence and physical limitations.   **🚩 Summary Table of Late Signs and Symptoms:**   \| **Signs and Symptoms** \| **Specific Observations** \| \| --- \| --- \| \| **Severe Weakness** \| Cannot lift objects or complete simple physical tasks. \| \| **Marked Muscle Atrophy** \| Prominent bone structure, reduced muscle definition. \| \| **Functional Impairment** \| Difficulty standing, walking, performing daily tasks. \| \| **Frequent Falls** \| Regular falls or balance issues causing injury. \| \| **Loss of Independence** \| Dependence on caregivers or assistive devices. \| \| **Chronic Fatigue** \| Persistent exhaustion affecting all activities. \| \| **Mobility Issues** \| Severe walking limitations; reliance on mobility aids. \| \| **Serious Health Risks** \| Increased fractures, chronic illness, hospitalizations. \| \| **Psychological Impact** \| Depression, anxiety, isolation, diminished quality of life. \|   **⚠️ Clinical Implications:**   - Late-stage sarcopenia significantly reduces quality of life and independence. - Intensive intervention (physical therapy, nutritional support, medical management) is necessary, even if restoration of full function becomes difficult. - Emphasis shifts towards preventing further decline, managing complications, and improving quality of life.   Identifying and intervening earlier can substantially prevent reaching these severe stages. |
| **Topic: Diagnosis** | |  |  |  |
| 13 | How do we diagnose sarcopenia? | (First search) Diagnosing sarcopenia involves assessing muscle mass, muscle strength, and physical performance. It requires a combination of clinical evaluation, imaging techniques, and functional tests. The diagnostic process may vary slightly depending on the guidelines used (e.g., **EWGSOP2** - European Working Group on Sarcopenia in Older People, **AWGS** - Asian Working Group for Sarcopenia, or **FNIH** - Foundation for the National Institutes of Health). Here’s a step-by-step overview:  **1. Initial Screening**   - **Risk Assessment:** Identify individuals at risk based on factors like age (typically ≥65 years), frailty, chronic illness, or unintentional weight loss. - **Questionnaires:** Tools like the **SARC-F** questionnaire (Strength, Assistance with walking, Rising from a chair, Climbing stairs, and Falls) can help screen for sarcopenia.   **2. Assessment of Muscle Strength**   - **Handgrip Strength:** Measured using a handheld dynamometer. Low grip strength is a key indicator of sarcopenia.   - **EWGSOP2 Cutoffs:** <27 kg for men, <16 kg for women.   - **AWGS Cutoffs:** <28 kg for men, <18 kg for women. - **Chair Stand Test:** Measures the time it takes to rise from a chair five times without using arms. Slower times suggest reduced leg strength.   **3. Measurement of Muscle Mass**   - **Dual-Energy X-ray Absorptiometry (DXA):** Gold standard for measuring lean muscle mass. Provides accurate data on appendicular skeletal muscle mass (ASM).   - **EWGSOP2 Cutoffs:** Appendicular skeletal muscle index (ASMI) <7.0 kg/m² for men, <5.5 kg/m² for women. - **Bioelectrical Impedance Analysis (BIA):** A simpler, non-invasive method to estimate muscle mass. - **Computed Tomography (CT) or Magnetic Resonance Imaging (MRI):** Highly accurate but less commonly used due to cost and accessibility.   **4. Evaluation of Physical Performance**   - **Gait Speed Test:** Measures walking speed over a short distance (e.g., 4 meters). Slow gait speed (<0.8 m/s) is a marker of sarcopenia. - **Short Physical Performance Battery (SPPB):** Assesses balance, gait, and chair stand performance. - **Timed Up and Go Test (TUG):** Measures the time it takes to rise from a chair, walk 3 meters, turn, and sit back down.   **5. Diagnostic Criteria**  Based on the findings, sarcopenia is classified into stages:   - **Probable Sarcopenia:** Low muscle strength. - **Confirmed Sarcopenia:** Low muscle strength + low muscle mass. - **Severe Sarcopenia:** Low muscle strength + low muscle mass + poor physical performance.   **6. Additional Investigations**   - **Blood Tests:** To rule out contributing factors like vitamin D deficiency, hormonal imbalances (e.g., low testosterone), or chronic inflammation. - **Nutritional Assessment:** Evaluate dietary protein intake and overall nutritional status.   **Importance of Early Diagnosis**  Early diagnosis allows for timely interventions, such as resistance training, nutritional optimization, and lifestyle changes, to slow or reverse muscle loss and improve outcomes. If sarcopenia is suspected, consult a healthcare professional for a comprehensive evaluation.  (Second search) Diagnosing **sarcopenia** involves assessing **muscle mass**, **muscle strength**, and **physical performance**. The process typically includes a combination of clinical evaluation, imaging tests, and functional assessments. Here’s a step-by-step guide to how sarcopenia is diagnosed:  1. Clinical Evaluation   - **Medical History**:   - Assess risk factors such as age, chronic diseases, physical inactivity, and malnutrition.   - Review symptoms like muscle weakness, fatigue, or difficulty performing daily activities. - **Physical Examination**:   - Check for visible signs of muscle wasting (e.g., thin arms or legs).   - Evaluate mobility, balance, and posture.   2. Muscle Mass Measurement   - **Dual-Energy X-ray Absorptiometry (DEXA)**:   - Measures muscle mass, fat mass, and bone density.   - Provides accurate and detailed results. - **Bioelectrical Impedance Analysis (BIA)**:   - Estimates muscle mass by measuring electrical resistance in the body.   - Quick, non-invasive, and widely available. - **Magnetic Resonance Imaging (MRI)** or **Computed Tomography (CT)**:   - Provides detailed images of muscle mass.   - Less commonly used due to cost and availability. - **Anthropometric Measurements**:   - Measures muscle mass indirectly using skinfold thickness or limb circumference.   - Less precise than imaging techniques.   3. Muscle Strength Assessment   - **Handgrip Strength**:   - Measured using a handheld dynamometer.   - Low grip strength is a key indicator of sarcopenia. - **Leg Strength**:   - Assessed using a leg press or chair stand test (e.g., time taken to stand up from a chair five times). - **Knee Extension Strength**:   - Measured using a dynamometer to assess quadriceps strength.   4. Physical Performance Tests   - **Gait Speed**:   - Measures walking speed over a short distance (e.g., 4 meters).   - Slow gait speed (<0.8 meters/second) is a marker of sarcopenia. - **Short Physical Performance Battery (SPPB)**:   - Combines tests of balance, gait speed, and chair stands to assess overall physical function. - **Timed Up and Go (TUG) Test**:   - Measures the time taken to stand up from a chair, walk 3 meters, turn around, and sit back down. - **6-Minute Walk Test**:   - Assesses endurance by measuring the distance walked in 6 minutes.   5. Diagnostic Criteria   - **European Working Group on Sarcopenia in Older People (EWGSOP2)**:   - **Probable Sarcopenia**: Low muscle strength (e.g., grip strength or chair stand test).   - **Confirmed Sarcopenia**: Low muscle strength + low muscle mass (measured by DEXA, BIA, or MRI).   - **Severe Sarcopenia**: Low muscle strength + low muscle mass + poor physical performance (e.g., slow gait speed). - **Asian Working Group for Sarcopenia (AWGS)**:   - Similar criteria but with adjusted cut-off values for Asian populations.   6. Additional Tests   - **Blood Tests**:   - Check for inflammation, hormonal imbalances, or nutrient deficiencies (e.g., vitamin D, testosterone, or insulin). - **Bone Density Test**:   - Assess for osteoporosis, which often coexists with sarcopenia.   Summary of Diagnostic Steps   1. **Clinical Evaluation**: Assess symptoms, risk factors, and physical signs. 2. **Muscle Mass Measurement**: Use DEXA, BIA, or other imaging techniques. 3. **Muscle Strength Assessment**: Test grip strength, leg strength, or knee extension strength. 4. **Physical Performance Tests**: Evaluate gait speed, SPPB, TUG, or 6-minute walk test. 5. **Diagnostic Criteria**: Apply EWGSOP2 or AWGS criteria to confirm sarcopenia. 6. **Additional Tests**: Blood tests or bone density tests if needed.   When to Seek Help  If you or a loved one is experiencing muscle weakness, fatigue, or difficulty performing daily activities, consult a healthcare provider. Early diagnosis and intervention can help slow the progression of sarcopenia and improve quality of life.  Management and Prevention   - **Exercise**: Resistance training, aerobic exercise, and balance training. - **Nutrition**: High-protein diet, adequate calories, and essential nutrients (e.g., vitamin D, calcium). - **Lifestyle Changes**: Stay active, quit smoking, and limit alcohol. - **Medical Support**: Address underlying conditions, hormone therapy, or supplements as needed.   By following these steps, healthcare providers can accurately diagnose sarcopenia and develop a personalized treatment plan to manage the condition effectively. | Diagnosing sarcopenia involves a comprehensive assessment of muscle mass, muscle strength, and physical performance. Here's a breakdown of the methods and criteria used:  **1. Assessment of Muscle Mass:**   - **Dual-Energy X-ray Absorptiometry (DXA or DEXA):** This is considered the gold standard for measuring body composition, including muscle mass. It provides precise measurements of lean mass (muscle), fat mass, and bone density. The Appendicular Lean Mass Index (ALMI), calculated from DXA, is often used to define low muscle mass. - **Bioelectrical Impedance Analysis (BIA):** A less expensive and more accessible method that estimates body composition by measuring the resistance of body tissues to a small electrical current. BIA is less accurate than DXA but can be useful for monitoring changes in muscle mass over time. - **Computed Tomography (CT) and Magnetic Resonance Imaging (MRI):** These imaging techniques provide detailed cross-sectional images of the body and can accurately measure muscle size and quality. However, they are more expensive and involve radiation exposure (CT), so they are typically used for research purposes or when more detailed information is needed. - **Anthropometry:** Simple measurements like mid-arm circumference and calf circumference can be used to estimate muscle mass, but they are less accurate than imaging techniques.   **2. Assessment of Muscle Strength:**   - **Grip Strength:** Measured using a hand dynamometer. This is a simple, reliable, and widely used test to assess overall muscle strength. Low grip strength is a strong predictor of adverse health outcomes. - **Chair Stand Test:** Measures the number of times a person can stand up from a seated position in a specified time period.   **3. Assessment of Physical Performance:**   - **Gait Speed (Walking Speed):** Measures the time it takes to walk a specified distance (e.g., 4 meters). Slow gait speed is a key indicator of functional decline and is strongly associated with adverse outcomes. - **Short Physical Performance Battery (SPPB):** A composite measure that assesses lower extremity function through a series of tests, including gait speed, chair stand test, and balance tests. - **Timed Up and Go (TUG) Test:** Measures the time it takes to stand up from a chair, walk a short distance (e.g., 3 meters), turn around, and sit back down. This test assesses mobility, balance, and coordination. - **Stair Climb Power Test:** Measures the time it takes to climb a flight of stairs, providing an indication of lower extremity power.   **Diagnostic Criteria:**  Several organizations have proposed diagnostic criteria for sarcopenia. One of the most widely used is the European Working Group on Sarcopenia in Older People (EWGSOP) criteria, which has been updated over time. The updated EWGSOP2 criteria (2018) recommends the following approach:   1. **Suspect Sarcopenia:** Identify individuals with low muscle strength (e.g., low grip strength). 2. **Confirm Sarcopenia:** In individuals with low muscle strength, confirm the diagnosis by assessing muscle mass. 3. **Assess Severity:** Assess physical performance to determine the severity of sarcopenia.   **EWGSOP2 Diagnostic Algorithm:**   - **Possible Sarcopenia:** Low muscle strength (Grip strength <27 kg for men and <16 kg for women) - **Confirmed Sarcopenia:** Low muscle strength + low muscle mass (ALMI based on DXA, adjusted for height squared) - **Severe Sarcopenia:** Low muscle strength + low muscle mass + low physical performance (e.g., gait speed <0.8 m/s or low SPPB score)   Other diagnostic criteria exist, such as those from the International Working Group on Sarcopenia (IWGS) and the Foundation for the National Institutes of Health (FNIH) Sarcopenia Project. These criteria may use slightly different cut-off values for muscle mass, strength, and performance.  **Diagnostic Tools**   - **SARC-F Questionnaire:** A simple 5-item questionnaire that can be used to screen for sarcopenia. It assesses strength, assistance with walking, rising from a chair, climbing stairs, and falls. A score of ≥4 indicates possible sarcopenia and warrants further evaluation.   In summary, diagnosing sarcopenia requires a comprehensive assessment of muscle mass, strength, and physical performance, using standardized and validated methods. The EWGSOP2 criteria provide a practical framework for diagnosing and staging the severity of sarcopenia.  Related searches:   - [diagnosing sarcopenia](https://www.google.com/search?q=diagnosing+sarcopenia&client=app-vertex-grounding-quora-poe)   [EWGSOP2 criteria for sarcopenia](https://www.google.com/search?q=EWGSOP2+criteria+for+sarcopenia&client=app-vertex-grounding-quora-poe) | Diagnosing **sarcopenia** typically involves a combination of clinical assessment, muscle strength testing, physical performance measures, and imaging or other methods to quantify muscle mass.  The European Working Group on Sarcopenia in Older People (EWGSOP2, 2019) criteria is widely used for diagnosing sarcopenia:  **📌 EWGSOP2 Diagnostic Criteria for Sarcopenia:**  Diagnosis involves assessing these **three key components**:  **1. Muscle Strength → 2. Muscle Quantity → 3. Physical Performance**   - **Probable sarcopenia:** Low **muscle strength** only. - **Confirmed sarcopenia:** Low **muscle strength** + low **muscle mass**. - **Severe sarcopenia:** Low **muscle strength** + low **muscle mass** + low **physical performance**.   **✅ Step-by-Step Diagnostic Approach:**  **🔹 Step 1: Screening and Clinical Suspicion**   - History of weakness, falls, or reduced mobility. - Clinical examination indicating reduced muscle bulk, strength, and function.   **🔹 Step 2: Muscle Strength Assessment**   - **Handgrip Strength:**   - Assessed using a handheld dynamometer.   - EWGSOP2 defines low grip strength as:     - Men: <27 kg     - Women: <16 kg - **Chair Stand Test (sit-to-stand):**   - Evaluates leg strength by measuring the time needed to rise from sitting five times without arms assistance.   - 15 seconds indicates reduced muscle strength.   **🔹 Step 3: Muscle Mass Measurement**  Typically done using imaging or bioelectrical methods:   - **Dual-energy X-ray absorptiometry (DXA):** Most common, measures appendicular skeletal muscle mass (ASM). - **Bioelectrical Impedance Analysis (BIA):** Non-invasive, quick alternative for muscle mass estimation. - **Computed Tomography (CT) or MRI:** Gold standard but less common due to cost and availability.   **🔹 Step 4: Physical Performance Evaluation**  Tests include:   - **Gait speed:**   - Timed 4-meter walk test   - Low performance: <0.8 m/s - **Short Physical Performance Battery (SPPB):**   - Balance, gait speed, and chair stand combined   - Scores ≤8 indicate poor performance. - **Timed Up-and-Go (TUG) test:**   - 20 seconds suggests significant functional impairment.   **📊 Summary of Diagnostic Criteria and Cut-offs:**   \| **Component** \| **Test Methods** \| **EWGSOP2 Cut-off points** \| \| --- \| --- \| --- \| \| **Muscle Strength** \| Handgrip strength \| Men: <27 kg, Women: <16 kg \| \|  \| Chair Stand Test (5 repetitions) \| >15 seconds \| \| **Muscle Mass** \| DXA, BIA, CT, MRI \| Low appendicular muscle mass (DXA/BIA) \| \| **Physical Performance** \| Gait Speed \| ≤0.8 m/s \| \|  \| Short Physical Performance Battery (SPPB) \| ≤8 points \| \|  \| Timed Up-and-Go (TUG) \| >20 seconds \|   **📝 Example Diagnostic Pathway:**   1. **Clinical suspicion** due to weakness, frequent falls, and reduced activity. 2. **Measure handgrip strength or chair-stand test**:    - If abnormal → probable sarcopenia; continue to muscle mass assessment. 3. **Measure muscle mass via DXA or BIA**:    - If muscle mass low → confirmed sarcopenia; proceed to physical performance testing. 4. **Measure physical performance (gait speed, SPPB, or TUG)**:    - If impaired → severe sarcopenia diagnosed.   **⚠️ Clinical Importance:**   - Accurate diagnosis using established guidelines allows targeted interventions:   - **Exercise training (especially resistance training)**   - **Nutrition improvement (protein, vitamin D)**   - **Medical interventions** to manage underlying conditions.   Proper diagnosis significantly improves patient outcomes and quality of life. |
| 14 | What should I do if I suspect I have sarcopenia? | (First search) If you suspect you have sarcopenia, it’s important to take proactive steps to address the condition and prevent further muscle loss. Here’s a practical guide on what to do:  **1. Consult a Healthcare Professional**   - **Primary Care Physician or Geriatrician:** Start by discussing your concerns with your doctor. They can evaluate your symptoms, perform initial tests, and refer you to specialists if needed. - **Diagnostic Evaluation:** Your doctor may assess muscle strength, muscle mass, and physical performance using tools like grip strength tests, DXA scans, or gait speed tests.   **2. Engage in Resistance Training**   - **Strength Training:** Incorporate resistance exercises (e.g., weightlifting, resistance bands) at least 2–3 times per week to build and maintain muscle mass. - **Focus on Major Muscle Groups:** Include exercises for the legs, arms, back, and core. - **Seek Guidance:** Consider working with a physical therapist or certified trainer to ensure proper technique and safety.   **3. Optimize Your Nutrition**   - **Increase Protein Intake:** Aim for 1.0–1.2 grams of protein per kilogram of body weight daily. Include high-quality protein sources like lean meats, fish, eggs, dairy, legumes, and plant-based proteins. - **Spread Protein Intake:** Distribute protein intake evenly across meals to maximize muscle protein synthesis. - **Vitamin D Supplementation:** If deficient, take vitamin D supplements as recommended by your doctor to support muscle health. - **Balanced Diet:** Ensure adequate intake of calories, vitamins, and minerals to support overall health.   **4. Stay Physically Active**   - **Aerobic Exercise:** Engage in activities like walking, swimming, or cycling to improve cardiovascular health and endurance. - **Balance and Flexibility Exercises:** Incorporate yoga or tai chi to enhance balance and reduce the risk of falls.   **5. Address Underlying Conditions**   - **Chronic Illnesses:** Manage conditions like diabetes, heart disease, or hormonal imbalances that may contribute to muscle loss. - **Medication Review:** Discuss with your doctor whether any medications you’re taking might affect muscle health.   **6. Monitor Progress**   - **Track Strength and Function:** Regularly assess improvements in muscle strength, mobility, and physical performance. - **Follow-Up Appointments:** Schedule regular check-ups with your healthcare provider to monitor your condition and adjust your plan as needed.   **7. Lifestyle Modifications**   - **Avoid Sedentary Behavior:** Reduce prolonged sitting and incorporate more movement into your daily routine. - **Sleep and Stress Management:** Prioritize quality sleep and manage stress to support overall health and recovery.   **8. Seek Support**   - **Nutritionist or Dietitian:** For personalized dietary advice. - **Physical Therapist:** For tailored exercise programs, especially if you have mobility limitations. - **Support Groups:** Connect with others facing similar challenges for motivation and advice.   **Early Intervention is Key**  The earlier you address sarcopenia, the better your chances of slowing or reversing muscle loss and maintaining independence. If you suspect you have sarcopenia, don’t delay—take action today to improve your muscle health and overall well-being.  (Second search) If you suspect you have **sarcopenia** (the age-related loss of muscle mass, strength, and function), it’s important to take proactive steps to confirm the diagnosis and address the condition early. Here’s what you should do:  1. Consult a Healthcare Provider   - **Primary Care Doctor**: Start by discussing your concerns with your primary care physician. - **Specialists**: You may be referred to a geriatrician, endocrinologist, or physical therapist for further evaluation.   2. Diagnostic Evaluation   - **Medical History**: Share your symptoms, such as muscle weakness, fatigue, or difficulty performing daily activities. - **Physical Exam**: Your doctor will check for signs of muscle wasting, poor balance, or mobility issues. - **Tests**:   - **Muscle Mass Measurement**: DEXA, BIA, or MRI to assess muscle mass.   - **Muscle Strength Assessment**: Handgrip strength or chair stand test.   - **Physical Performance Tests**: Gait speed, SPPB, or TUG test.   3. Address Underlying Causes   - **Chronic Conditions**: Manage diseases like diabetes, heart disease, or kidney disease. - **Medication Review**: Discuss medications that may contribute to muscle loss (e.g., corticosteroids). - **Nutrient Deficiencies**: Check for deficiencies in protein, vitamin D, or calcium.   4. Start an Exercise Program   - **Resistance Training**:   - Use weights, resistance bands, or bodyweight exercises (e.g., squats, lunges, push-ups).   - Aim for **2–3 sessions per week**. - **Aerobic Exercise**:   - Activities like walking, swimming, or cycling to improve cardiovascular health.   - Aim for **150 minutes per week** of moderate-intensity exercise. - **Balance Training**:   - Yoga or tai chi to improve stability and reduce fall risk. - **Physical Therapy**: Work with a therapist to develop a safe and effective exercise plan.   5. Optimize Nutrition   - **High-Protein Diet**:   - Aim for **1.0–1.2 grams of protein per kilogram of body weight per day** (higher for older adults).   - Include lean meats, fish, eggs, dairy, legumes, and plant-based proteins. - **Essential Nutrients**:   - Ensure adequate intake of **vitamin D**, **calcium**, and **omega-3 fatty acids**. - **Balanced Diet**:   - Focus on nutrient-dense foods like fruits, vegetables, whole grains, and healthy fats. - **Supplements**:   - Consider protein shakes, vitamin D, or calcium supplements if dietary intake is insufficient.   6. Make Lifestyle Changes   - **Stay Active**: Avoid prolonged sitting and incorporate movement into your daily routine. - **Quit Smoking**: Smoking accelerates muscle loss. - **Limit Alcohol**: Excessive alcohol consumption can impair muscle repair. - **Sleep Well**: Aim for **7–9 hours of quality sleep per night** to support muscle recovery.   7. Monitor Progress   - **Regular Check-Ups**: Track muscle mass, strength, and physical performance over time. - **Adjust Interventions**: Modify your exercise or nutrition plan based on progress and feedback.   8. Seek Support   - **Family and Friends**: Involve loved ones to help you stay motivated and active. - **Support Groups**: Connect with others experiencing sarcopenia for shared experiences and encouragement. - **Mental Health**: Seek counseling or therapy if you’re feeling anxious or depressed about your condition.   9. Prevent Falls   - **Home Safety**:   - Remove tripping hazards, install grab bars, and improve lighting. - **Assistive Devices**:   - Use canes, walkers, or other devices if needed for stability.   10. Stay Positive   - **Set Realistic Goals**: Focus on small, achievable improvements in strength and function. - **Celebrate Progress**: Acknowledge and reward your efforts and achievements.   Summary  If you suspect sarcopenia, consult a healthcare provider for a thorough evaluation. Start an **exercise program**, optimize your **nutrition**, and make **lifestyle changes** to slow muscle loss and improve strength and function. Early intervention is key to managing sarcopenia and maintaining your independence and quality of life. | If you suspect you have sarcopenia, here's a step-by-step guide on what you should do:   1. **Self-Assessment and Awareness:**    - **Recognize the Symptoms:** Be aware of the early signs and symptoms of sarcopenia, such as decreased strength, reduced stamina, slowed walking speed, difficulty with physical tasks, and unintentional weight loss.    - **Use Screening Tools:** Consider using a simple screening tool like the SARC-F questionnaire to assess your risk of sarcopenia. A score of ≥4 indicates possible sarcopenia and warrants further evaluation.    - **Monitor Your Physical Function:** Pay attention to any changes in your ability to perform daily activities, such as lifting objects, climbing stairs, or walking. 2. **Consult a Healthcare Professional:**    - **Schedule an Appointment:** Make an appointment with your primary care physician or a geriatric specialist.    - **Describe Your Symptoms:** Clearly explain your symptoms and concerns to your doctor. Be prepared to discuss your medical history, lifestyle, and any relevant family history. 3. **Medical Evaluation:**    - **Physical Exam:** Your doctor will perform a physical exam to assess your muscle strength, balance, and overall physical function.    - **Diagnostic Tests:** Your doctor may order tests to assess your muscle mass, strength, and physical performance. These tests may include:      - **Grip Strength Test:** To measure your handgrip strength using a dynamometer.      - **Gait Speed Test:** To measure your walking speed.      - **Chair Stand Test:** To assess your lower body strength.      - **DXA Scan:** To measure your muscle mass, fat mass, and bone density. 4. **Diagnosis and Management Plan:**    - **Receive a Diagnosis:** Based on the results of the tests and examination, your doctor will determine if you meet the diagnostic criteria for sarcopenia.    - **Develop a Management Plan:** If you are diagnosed with sarcopenia, your doctor will work with you to develop a personalized management plan. This plan may include:      - **Exercise Recommendations:**        - **Resistance Training:** To build muscle mass and strength.        - **Aerobic Exercise:** To improve cardiovascular health and overall fitness.      - **Nutritional Guidance:**        - **Protein Intake:** To ensure you are getting enough protein to support muscle growth and repair. Aim for 1.0-1.2 grams of protein per kilogram of body weight per day.        - **Vitamin D and Calcium:** To support bone health and muscle function.        - **Overall Balanced Diet:** To ensure you are getting all the nutrients you need.      - **Medications and Supplements:**        - Your doctor may recommend certain medications or supplements to help manage sarcopenia, such as vitamin D supplements or medications to treat underlying conditions. 5. **Lifestyle Modifications:**    - **Incorporate Exercise into Your Routine:** Make regular exercise a part of your daily routine. Aim for at least 150 minutes of moderate-intensity aerobic exercise per week, as well as resistance training exercises at least two days per week.    - **Optimize Your Diet:** Focus on eating a balanced diet that is rich in protein, fruits, vegetables, and whole grains.    - **Stay Active:** Find ways to stay active throughout the day, such as taking walks, gardening, or participating in hobbies that you enjoy.    - **Quit Smoking:** If you smoke, quitting can improve your overall health and reduce your risk of sarcopenia.    - **Limit Alcohol Consumption:** Excessive alcohol consumption can contribute to muscle loss. 6. **Follow-Up and Monitoring:**    - **Regular Check-Ups:** Schedule regular follow-up appointments with your doctor to monitor your progress and adjust your management plan as needed.    - **Track Your Progress:** Keep track of your muscle strength, physical function, and overall well-being.    - **Be Patient and Persistent:** Managing sarcopenia is an ongoing process. Be patient with yourself and stay committed to your management plan.   By taking these steps, you can proactively address your concerns about sarcopenia and work with your healthcare provider to develop a plan to maintain or improve your muscle health and overall well-being.  Related searches:   - [sarcopenia diagnosis and treatment](https://www.google.com/search?q=sarcopenia+diagnosis+and+treatment&client=app-vertex-grounding-quora-poe)   [what to do if you suspect sarcopenia](https://www.google.com/search?q=what+to+do+if+you+suspect+sarcopenia&client=app-vertex-grounding-quora-poe) | If you suspect that you have **sarcopenia**, it's important to address it proactively. Here's a structured approach on what you should do:  **✅ 1. Consult a Healthcare Professional**  **Who to consult?**   - **Primary Care Physician (Family Doctor)** - **Geriatrician** - **Endocrinologist** - **Physiatrist (Rehabilitation Specialist)** - **Dietitian or Nutritionist** - **Physical Therapist**   They can perform diagnostic tests, assess severity, and rule out other underlying conditions.  **✅ 2. Undergo Appropriate Assessment and Testing**  **Typical tests include:**   - **Muscle Strength Assessment** (handgrip dynamometry, chair-stand tests) - **Muscle Mass Measurement** (DXA, Bioelectrical Impedance Analysis) - **Physical Performance Evaluation** (Gait speed, balance, mobility tests)   **✅ 3. Start an Exercise Program (Resistance & Aerobic Training)**   - **Resistance Training (Strength Training)**   - Most effective method to improve muscle mass and strength.   - Aim for at least **2-3 sessions per week**.   - Include exercises like squats, lunges, leg press, and upper-body strengthening. - **Aerobic Exercise**   - Walking, cycling, swimming—ideally 150 minutes weekly.   - Improves cardiovascular health, mobility, and endurance. - **Balance and Flexibility Exercises**   - Yoga, tai chi, and balance exercises help reduce fall risk.   **Consider professional guidance (physical therapist or trainer)** for safety and effectiveness.  **✅ 4. Optimize Nutrition and Diet**   - **Increase Protein Intake**:   - Aim for approximately **1.0–1.5 grams protein/kg body weight daily**.   - Include protein-rich foods (e.g., lean meats, fish, eggs, legumes, dairy). - **Vitamin D and Calcium**:   - Important for muscle function and bone health.   - Consider supplementation if deficient. - **Balanced Diet**:   - Ensure sufficient calories and nutrients to support muscle maintenance and overall health.   - Consult a dietitian if necessary.   **✅ 5. Review and Manage Medications**   - Review your medications with your doctor, especially if you're on:   - **Steroids, proton pump inhibitors (PPIs), statins, chemotherapy drugs, or hormonal treatments** - Adjustments may reduce muscle loss risk.   **✅ 6. Address Underlying Conditions**   - Manage chronic diseases (e.g., diabetes, COPD, heart disease). - Control inflammation and hormonal imbalances if present.   **✅ 7. Lifestyle Modifications**   - **Reduce Sedentary Behavior**:   - Move more frequently throughout the day.   - Aim to limit extended periods of sitting or inactivity. - **Stay Socially and Mentally Active**:   - Social interaction and mental engagement improve overall well-being and encourage activity.   **📌 Action Plan Summary:**   \| **Action** \| **Specific Recommendations** \| \| --- \| --- \| \| **Professional Advice** \| Visit physician or specialist promptly. \| \| **Assessment** \| Muscle strength, mass, and function tests. \| \| **Exercise** \| Resistance, aerobic, balance exercises weekly. \| \| **Nutrition** \| Increased protein, vitamin D, balanced diet. \| \| **Medication Review** \| Adjust or reduce muscle-affecting medications. \| \| **Underlying Conditions** \| Manage chronic diseases effectively. \| \| **Lifestyle Changes** \| Increase daily movement; reduce sedentary habits. \|   **⚠️ When to Seek Immediate Medical Attention:**   - Severe muscle weakness, frequent falls, or rapid functional decline require prompt medical evaluation.   Taking these proactive steps early significantly improves outcomes, reduces complications, and enhances overall quality of life. |
| **Topic: Prevention and treatment** | | | | |
| 15 | Can sarcopenia be prevented? | Sarcopenia, the age-related loss of muscle mass, strength, and function, can be mitigated and potentially prevented through a combination of lifestyle interventions, dietary strategies, and medical management. While some degree of muscle loss is a natural part of aging, proactive measures can significantly slow its progression and maintain muscle health. Here are key strategies to prevent or delay sarcopenia:  **1. Regular Exercise**   - **Resistance Training:** Strength training (e.g., weightlifting, resistance bands) is the most effective way to build and maintain muscle mass. It stimulates muscle protein synthesis and improves strength. - **Aerobic Exercise:** Activities like walking, cycling, or swimming improve cardiovascular health and support overall muscle function. - **Balance and Flexibility Exercises:** Yoga, tai chi, or stretching routines can enhance mobility and reduce the risk of falls.   **2. Optimal Nutrition**   - **Protein Intake:** Consuming adequate high-quality protein (e.g., lean meats, fish, eggs, dairy, legumes) supports muscle maintenance and repair. Older adults may need more protein (1.0–1.2 g/kg of body weight per day) to counteract age-related muscle loss. - **Essential Amino Acids:** Leucine, in particular, is critical for muscle protein synthesis and can be found in foods like eggs, soy, and dairy. - **Vitamin D:** Adequate vitamin D levels are essential for muscle function. Sun exposure, fortified foods, or supplements can help maintain optimal levels. - **Omega-3 Fatty Acids:** Found in fatty fish, flaxseeds, and walnuts, omega-3s may reduce inflammation and support muscle health. - **Balanced Diet:** A diet rich in fruits, vegetables, whole grains, and healthy fats provides the nutrients needed for overall health and muscle maintenance.   **3. Lifestyle Modifications**   - **Avoid Sedentary Behavior:** Prolonged inactivity accelerates muscle loss. Incorporate regular movement into daily routines. - **Healthy Weight Management:** Both obesity and underweight can contribute to sarcopenia. Maintaining a healthy weight supports muscle health. - **Smoking and Alcohol Cessation:** Smoking and excessive alcohol consumption can impair muscle function and accelerate muscle loss.   **4. Medical and Hormonal Management**   - **Address Chronic Conditions:** Manage conditions like diabetes, cardiovascular disease, or hormonal imbalances that can contribute to muscle loss. - **Hormone Replacement Therapy (HRT):** In some cases, HRT may be considered for postmenopausal women or men with low testosterone levels, but this should be discussed with a healthcare provider. - **Medication Review:** Some medications (e.g., corticosteroids) can contribute to muscle loss. Review medications with a doctor to minimize side effects.   **5. Early Prevention**   - **Start Early:** Building muscle mass and strength in younger years creates a "reserve" that can help offset age-related losses. - **Regular Monitoring:** Assess muscle mass, strength, and function periodically, especially in older adults, to detect sarcopenia early.   **6. Social and Mental Health**   - **Stay Active and Engaged:** Social engagement and mental stimulation can improve motivation to stay physically active. - **Manage Stress:** Chronic stress can contribute to muscle loss. Practices like mindfulness, meditation, or therapy can help.   **Summary**  While sarcopenia cannot be entirely prevented due to its association with aging, its progression can be significantly slowed or mitigated through:   - Regular resistance and aerobic exercise, - A protein-rich, nutrient-dense diet, - Healthy lifestyle habits, - Medical management of underlying conditions.   Starting these strategies early and maintaining them throughout life is key to preserving muscle mass, strength, and function as you age. | Yes, sarcopenia can often be prevented or at least significantly delayed and mitigated through proactive lifestyle choices and interventions. Here's a breakdown of strategies that can help:   - **Regular Exercise:**   - **Resistance Training (Strength Training):** This is the most effective way to prevent and combat sarcopenia. Resistance training stimulates muscle protein synthesis, helping to build and maintain muscle mass and strength. Examples include lifting weights, using resistance bands, and doing bodyweight exercises like squats and push-ups. Aim for at least two to three sessions per week, targeting all major muscle groups.   - **Aerobic Exercise:** While not as effective as resistance training for building muscle mass, aerobic exercise (e.g., walking, jogging, swimming, cycling) improves cardiovascular health, endurance, and overall fitness, which can indirectly support muscle health and function. Aim for at least 150 minutes of moderate-intensity or 75 minutes of vigorous-intensity aerobic exercise per week.   - **Balance and Flexibility Exercises:** Incorporating balance and flexibility exercises (e.g., yoga, tai chi) can improve stability, coordination, and range of motion, reducing the risk of falls and injuries. - **Adequate Protein Intake:**   - **Consume Sufficient Protein:** Protein is essential for muscle protein synthesis. Aim for a daily protein intake of 1.0-1.2 grams per kilogram of body weight, or even higher (1.2-1.5 g/kg) for older adults or those with increased protein needs.   - **Distribute Protein Intake:** Distribute your protein intake evenly throughout the day, rather than consuming most of it in one meal. This can help maximize muscle protein synthesis.   - **Choose High-Quality Protein Sources:** Include a variety of high-quality protein sources in your diet, such as lean meats, poultry, fish, eggs, dairy products, legumes, and soy products. - **Sufficient Caloric Intake:**   - **Avoid Undereating:** Ensure you are consuming enough calories to meet your energy needs. Chronic undereating can lead to muscle loss. - **Vitamin D and Calcium:**   - **Maintain Adequate Vitamin D Levels:** Vitamin D plays a crucial role in muscle function and bone health. Get your vitamin D levels checked and take supplements if needed to maintain adequate levels (typically above 30 ng/mL).   - **Ensure Adequate Calcium Intake:** Calcium is important for bone health and muscle function. Include calcium-rich foods in your diet, such as dairy products, leafy green vegetables, and fortified foods. - **Healthy Lifestyle Habits:**   - **Quit Smoking:** Smoking can impair muscle protein synthesis and increase the risk of sarcopenia.   - **Limit Alcohol Consumption:** Excessive alcohol consumption can contribute to muscle loss and other health problems.   - **Manage Chronic Conditions:** Effectively manage any chronic conditions you may have, such as diabetes, heart disease, or kidney disease, as these can contribute to sarcopenia. - **Regular Medical Check-ups:**   - **Screening for Sarcopenia:** Talk to your doctor about screening for sarcopenia, especially if you are over the age of 65 or have risk factors for the condition.   - **Monitoring Muscle Mass and Function:** Your doctor can monitor your muscle mass, strength, and physical function over time and recommend interventions if needed. - **Stay Socially Active:**   - **Combat Social Isolation:** Social isolation and loneliness can contribute to physical inactivity and poor nutrition, increasing the risk of sarcopenia. Stay connected with friends and family and participate in social activities. - **Hormone Replacement Therapy (HRT):**   - **Consider HRT (Under Medical Supervision):** In some cases, hormone replacement therapy (HRT) may be considered to help prevent or treat sarcopenia, particularly in individuals with hormonal imbalances. However, HRT should only be used under the guidance and supervision of a healthcare professional, as it can have potential risks and side effects. - **Medications:**   - **Certain medications:** Some medications, such as ACE inhibitors, have been shown to have a positive impact on muscle mass.   By adopting these strategies, you can significantly reduce your risk of developing sarcopenia and maintain your muscle health and function as you age. Early intervention is key, so start making these lifestyle changes as early as possible.  Related searches:   - [sarcopenia prevention](https://www.google.com/search?q=sarcopenia+prevention&client=app-vertex-grounding-quora-poe)   [how to prevent muscle loss with age](https://www.google.com/search?q=how+to+prevent+muscle+loss+with+age&client=app-vertex-grounding-quora-poe) | Yes, **sarcopenia** can be prevented or significantly delayed through targeted interventions, particularly if implemented early. Prevention strategies mainly focus on exercise, nutrition, lifestyle adjustments, and managing underlying conditions.  **✅ Effective Strategies to Prevent Sarcopenia:**  **🏋️‍♀️ 1. Regular Exercise (Especially Resistance Training)**   - **Resistance (strength) training:**   - Most effective method for preventing muscle loss.   - Recommended **2–3 times per week**.   - Exercises: squats, lunges, leg presses, resistance bands, weightlifting. - **Aerobic Exercise:**   - Improves cardiovascular health and maintains muscle endurance.   - Recommended **150 minutes/week** (e.g., walking, swimming, cycling). - **Balance and Flexibility Exercises:**   - Tai chi, yoga, and stretching to improve coordination and reduce fall risk.   **🍽️ 2. Adequate Nutrition**   - **High-quality protein intake:**   - Recommended: **1.0–1.5 g protein/kg body weight/day**.   - Sources: lean meats, dairy, fish, eggs, legumes, and plant proteins. - **Vitamin D and Calcium:**   - Essential for muscle strength and bone health.   - Supplement if dietary intake or sun exposure is insufficient. - **Balanced Diet:**   - Ensure sufficient calories and nutrients (omega-3 fatty acids, antioxidants, vitamins, minerals) for muscle health.   **🚶‍♂️ 3. Lifestyle Modifications**   - **Minimize Sedentary Behavior:**   - Frequent breaks from prolonged sitting.   - Daily physical activity routines. - **Maintain a Healthy Weight:**   - Avoid obesity or rapid weight loss to maintain muscle mass. - **Stay Socially Active:**   - Social engagement helps maintain activity levels and overall health.   **💊 4. Review and Adjust Medications**   - Regularly review medications, particularly those associated with muscle loss:   - Corticosteroids (prednisone)   - Proton Pump Inhibitors (PPIs)   - Chemotherapy drugs   - Androgen deprivation therapies (ADT)   **⚕️ 5. Management of Underlying Conditions**   - Proper management of chronic illnesses (diabetes, COPD, kidney disease, heart failure, cancer). - Control inflammation through diet, lifestyle, and medication as needed.   **📌 Summary of Preventive Strategies:**   \| **Strategy** \| **Recommendation** \| **Benefit** \| \| --- \| --- \| --- \| \| **Resistance Exercise** \| 2–3 sessions/week \| Maintain/increase muscle mass, strength \| \| **Aerobic Exercise** \| ≥150 min/week \| Maintain muscle endurance, cardiovascular health \| \| **Nutrition (Protein)** \| 1.0–1.5 g/kg/day \| Supports muscle growth and repair \| \| **Vitamin D/Calcium** \| Adequate daily intake \| Supports muscle function, bone health \| \| **Lifestyle Adjustments** \| Reduce sedentary time, maintain healthy weight \| Prevent muscle loss, obesity-related inflammation \| \| **Medication Review** \| Regular medical reviews \| Minimize medication-related muscle loss \| \| **Chronic Disease Management** \| Regular medical follow-up, effective disease control \| Reduce muscle loss due to chronic disease \|   **⚠️ Importance of Early Prevention:**   - Prevention strategies are most effective when implemented early—ideally starting from middle age (30s–50s). - However, significant improvements are still achievable at older ages.   By proactively adopting these strategies, sarcopenia can be effectively prevented or its progression greatly slowed, leading to better health, mobility, independence, and overall quality of life. |
| 16 | What is the best treatment for sarcopenia? | The best treatment for **sarcopenia** (age-related loss of muscle mass, strength, and function) involves a combination of **exercise**, **nutrition**, **lifestyle modifications**, and, in some cases, **medical interventions**. The goal is to preserve or rebuild muscle mass, improve strength, and enhance overall physical function. Here’s a comprehensive approach to treating sarcopenia:  **1. Exercise Interventions**   - **Resistance Training:**   - The cornerstone of sarcopenia treatment. Strength training (e.g., weightlifting, resistance bands) stimulates muscle protein synthesis and increases muscle mass and strength.   - Recommended: 2–3 sessions per week, targeting major muscle groups. - **Aerobic Exercise:**   - Activities like walking, cycling, or swimming improve cardiovascular health and support muscle function.   - Recommended: 150 minutes of moderate-intensity aerobic activity per week. - **Balance and Flexibility Training:**   - Exercises like yoga, tai chi, or stretching improve mobility and reduce the risk of falls.   - Recommended: Incorporate into weekly routines.   **2. Nutritional Support**   - **High-Quality Protein:**   - Adequate protein intake is essential for muscle repair and maintenance. Older adults may need 1.0–1.2 g of protein per kg of body weight daily, or even higher (up to 1.5 g/kg) for those with sarcopenia.   - Sources: Lean meats, fish, eggs, dairy, legumes, soy, and protein supplements if needed. - **Essential Amino Acids (Leucine):**   - Leucine, found in foods like eggs, dairy, and soy, is particularly important for stimulating muscle protein synthesis. - **Vitamin D:**   - Low vitamin D levels are linked to muscle weakness. Supplementation may improve muscle strength and function.   - Recommended: 800–1000 IU/day, or as advised by a healthcare provider. - **Omega-3 Fatty Acids:**   - Found in fatty fish, flaxseeds, and walnuts, omega-3s may reduce inflammation and support muscle health. - **Caloric Sufficiency:**   - Ensure adequate caloric intake to prevent muscle loss, especially in underweight or malnourished individuals.   **3. Lifestyle Modifications**   - **Avoid Sedentary Behavior:**   - Prolonged inactivity accelerates muscle loss. Incorporate regular movement into daily routines. - **Healthy Weight Management:**   - Maintain a healthy weight to support muscle health. - **Smoking and Alcohol Cessation:**   - Smoking and excessive alcohol consumption can impair muscle function and accelerate muscle loss.   **4. Medical and Hormonal Interventions**   - **Address Underlying Conditions:**   - Manage chronic diseases (e.g., diabetes, heart disease) or hormonal imbalances (e.g., low testosterone, thyroid disorders) that contribute to muscle loss. - **Hormone Replacement Therapy (HRT):**   - In some cases, HRT may be considered for postmenopausal women or men with low testosterone levels, but this should be discussed with a healthcare provider. - **Medication Review:**   - Some medications (e.g., corticosteroids) can contribute to muscle loss. Review medications with a doctor to minimize side effects.   **5. Emerging Therapies**   - **Myostatin Inhibitors:**   - Experimental drugs that block myostatin, a protein that limits muscle growth, are being studied for sarcopenia treatment. - **Selective Androgen Receptor Modulators (SARMs):**   - These compounds are being researched for their potential to increase muscle mass without the side effects of traditional anabolic steroids. - **Nutritional Supplements:**   - Creatine, beta-hydroxy-beta-methylbutyrate (HMB), and branched-chain amino acids (BCAAs) are being explored for their potential to support muscle health.   **6. Multidisciplinary Approach**   - **Physical Therapy:**   - A physical therapist can design a personalized exercise program to improve strength, balance, and mobility. - **Dietitian Support:**   - A registered dietitian can help optimize protein intake and overall nutrition. - **Regular Monitoring:**   - Assess muscle mass, strength, and function periodically to track progress and adjust treatment plans.   **Summary**  The best treatment for sarcopenia involves:   - **Regular resistance and aerobic exercise,** - **A protein-rich, nutrient-dense diet,** - **Lifestyle changes (e.g., avoiding inactivity, managing weight),** - **Medical management of underlying conditions.**   A personalized, multidisciplinary approach is key to effectively managing sarcopenia and improving quality of life. Early intervention and consistent adherence to these strategies yield the best results. | The best treatment for sarcopenia is a multifaceted approach that combines exercise, nutrition, and, in some cases, medication or supplementation. Here's a breakdown of the key components:  **1. Exercise:**   - **Resistance Training (Strength Training):** This is the cornerstone of sarcopenia treatment. Resistance training stimulates muscle protein synthesis, helping to build and maintain muscle mass and strength.   - **Types of Exercises:** Include exercises that target all major muscle groups (legs, arms, chest, back, shoulders, and core). Examples include:     - **Weightlifting:** Using free weights (dumbbells, barbells) or weight machines.     - **Resistance Bands:** Using elastic bands to provide resistance.     - **Bodyweight Exercises:** Using your own body weight for resistance (e.g., squats, lunges, push-ups, planks).   - **Intensity and Frequency:** Aim for at least two to three sessions per week, with each session lasting 30-60 minutes. Use a weight or resistance that challenges you but allows you to maintain good form. Gradually increase the weight or resistance as you get stronger. - **Aerobic Exercise:** While not as effective as resistance training for building muscle mass, aerobic exercise improves cardiovascular health, endurance, and overall fitness, which can indirectly support muscle health and function.   - **Types of Exercises:** Walking, jogging, swimming, cycling, dancing.   - **Intensity and Frequency:** Aim for at least 150 minutes of moderate-intensity or 75 minutes of vigorous-intensity aerobic exercise per week. - **Balance and Flexibility Exercises:** Incorporating balance and flexibility exercises (e.g., yoga, tai chi) can improve stability, coordination, and range of motion, reducing the risk of falls and injuries.   **2. Nutrition:**   - **Adequate Protein Intake:** Protein is essential for muscle protein synthesis.   - **Recommended Intake:** Aim for a daily protein intake of 1.0-1.2 grams per kilogram of body weight, or even higher (1.2-1.5 g/kg) for older adults or those with increased protein needs.   - **Distribution:** Distribute your protein intake evenly throughout the day, rather than consuming most of it in one meal.   - **Sources:** Choose high-quality protein sources, such as lean meats, poultry, fish, eggs, dairy products, legumes, and soy products. - **Sufficient Caloric Intake:** Ensure you are consuming enough calories to meet your energy needs. Chronic undereating can lead to muscle loss. - **Vitamin D and Calcium:**   - **Vitamin D:** Vitamin D plays a crucial role in muscle function and bone health. Get your vitamin D levels checked and take supplements if needed to maintain adequate levels (typically above 30 ng/mL).   - **Calcium:** Calcium is important for bone health and muscle function. Include calcium-rich foods in your diet, such as dairy products, leafy green vegetables, and fortified foods. - **Creatine Supplementation:**   - **Consider Creatine:** Creatine is a naturally occurring compound that can enhance muscle strength and power. Creatine supplementation may be beneficial for individuals with sarcopenia, especially when combined with resistance training. Consult with your doctor or a registered dietitian before taking creatine supplements. - **Omega-3 Fatty Acids:**   - **Include Omega-3s:** Omega-3 fatty acids, found in fatty fish, flaxseed, and walnuts, have anti-inflammatory properties and may help improve muscle protein synthesis.   **3. Medications and Supplements:**   - **Vitamin D Supplements:** If you have low vitamin D levels, your doctor may recommend taking vitamin D supplements. - **Testosterone Replacement Therapy (TRT):** In some cases, testosterone replacement therapy may be considered for men with low testosterone levels and sarcopenia. However, TRT should only be used under the guidance and supervision of a healthcare professional, as it can have potential risks and side effects. - **Growth Hormone (GH):** Growth hormone can increase muscle mass, but it also has potential side effects and is not generally recommended for the treatment of sarcopenia. - **Other Medications:** Some medications, such as ACE inhibitors, have been shown to have a positive impact on muscle mass.   **4. Lifestyle Modifications:**   - **Quit Smoking:** Smoking can impair muscle protein synthesis and increase the risk of sarcopenia. - **Limit Alcohol Consumption:** Excessive alcohol consumption can contribute to muscle loss and other health problems. - **Manage Chronic Conditions:** Effectively manage any chronic conditions you may have, such as diabetes, heart disease, or kidney disease, as these can contribute to sarcopenia. - **Stay Socially Active:** Social isolation and loneliness can contribute to physical inactivity and poor nutrition, increasing the risk of sarcopenia.   **5. Rehabilitation and Physical Therapy:**   - **Physical Therapy:** A physical therapist can help you develop a personalized exercise program and provide guidance and support to help you improve your muscle strength, balance, and physical function. - **Occupational Therapy:** An occupational therapist can help you adapt your environment and activities to make it easier to perform daily tasks.   **6. Regular Monitoring and Follow-Up:**   - **Regular Check-Ups:** Schedule regular follow-up appointments with your doctor to monitor your progress and adjust your treatment plan as needed. - **Track Your Progress:** Keep track of your muscle strength, physical function, and overall well-being.   The best treatment plan for sarcopenia is one that is tailored to your individual needs and goals. Work closely with your healthcare team to develop a plan that is safe, effective, and sustainable for you.  Related searches:   - [sarcopenia treatment guidelines](https://www.google.com/search?q=sarcopenia+treatment+guidelines&client=app-vertex-grounding-quora-poe)   [best exercises for sarcopenia](https://www.google.com/search?q=best+exercises+for+sarcopenia&client=app-vertex-grounding-quora-poe) | The best treatment approach for **sarcopenia** involves a combination of **exercise**, **nutrition**, and **lifestyle modifications**, often supported by medical management. Currently, **resistance training** coupled with appropriate nutrition is considered the most effective treatment.  **✅ Recommended Treatment Approaches for Sarcopenia:**  **🏋️‍♂️ 1. Resistance Exercise (Strength Training) *(Most Effective)***   - **Benefits:**   - Increases muscle mass, strength, and function.   - Improves balance, reduces fall risk, and enhances mobility. - **Recommendation:**   - Perform resistance exercises at least **2–3 times per week**.   - Exercises: weightlifting, resistance bands, bodyweight exercises (squats, lunges, push-ups, leg press).   **🍽️ 2. Nutritional Interventions**   - **Protein Supplementation:**   - Increase dietary protein intake (**1.0–1.5 g/kg body weight/day**).   - Include lean meat, fish, dairy, eggs, legumes, and high-quality protein supplements if needed. - **Vitamin D and Calcium:**   - Essential for muscle and bone health.   - Supplement if deficient (usually Vitamin D: 800–2000 IU/day). - **Balanced and Nutrient-Rich Diet:**   - Adequate caloric intake with emphasis on essential nutrients, antioxidants, omega-3 fatty acids, vitamins, and minerals.   **🚶‍♀️ 3. Aerobic Exercise and Physical Activity**   - **Benefits:**   - Enhances cardiovascular health, reduces inflammation, and improves muscle endurance.   - Improves insulin sensitivity and overall health. - **Recommendation:**   - Aim for at least **150 minutes/week** of moderate aerobic activity (brisk walking, cycling, swimming).   **⚕️ 4. Medical Management (Pharmacological Interventions)**   - Currently, there is **no widely approved drug specifically for sarcopenia**, but ongoing research includes:   - **Hormone Replacement Therapy (HRT):** Testosterone in men, selective estrogen receptor modulators (SERMs), growth hormone (limited and under clinical guidance).   - **Myostatin inhibitors:** Experimental treatments under research.   - **Anti-inflammatory agents:** Used in chronic inflammatory conditions that worsen sarcopenia.   - **Vitamin D and anabolic agents:** Can be used selectively under medical supervision.   **💊 5. Medication Review and Adjustment**   - Adjust medications associated with muscle loss (e.g., corticosteroids, chemotherapy, androgen deprivation therapies). - Optimize medications to manage chronic diseases effectively.   **🩺 6. Multidisciplinary Rehabilitation Programs**   - Comprehensive care involving:   - Physical therapists   - Dietitians or nutritionists   - Geriatricians, endocrinologists, or physiatrists (rehabilitation physicians)   **📌 Summary Table of Best Treatment Options:**   \| **Treatment Strategy** \| **Recommendations** \| **Primary Benefits** \| \| --- \| --- \| --- \| \| **Resistance Training** \| ≥2–3 times/week \| Increased muscle mass, strength, function \| \| **Nutrition** \| Protein: 1.0–1.5 g/kg/day, Vitamin D \| Supports muscle synthesis, strength, health \| \| **Aerobic Exercise** \| ≥150 min/week \| Improved endurance, metabolic health \| \| **Medication Review** \| Regular medical review, adjustments \| Reduced medication-induced muscle loss \| \| **Medical Management** \| Hormonal therapy (selectively), supplements \| Address underlying causes \| \| **Multidisciplinary Care** \| Physical therapy, nutritional counseling, medical oversight \| Comprehensive management of sarcopenia \|   **⚠️ Most Effective Evidence-based Approach:**   - **Resistance exercise combined with protein-rich nutrition** is currently the gold-standard, evidence-based approach and provides the most consistent, measurable benefit.   **🔑 Conclusion:**  The best treatment strategy is **multi-modal**, combining regular resistance exercise, optimal nutrition, and appropriate medical care. Implementing these strategies can significantly improve muscle strength, mass, functionality, and overall quality of life in individuals with sarcopenia. |
| 17 | Are there any medications to treat sarcopenia? | Currently, there are **no FDA-approved medications specifically for treating sarcopenia**. However, several medications and experimental therapies are being investigated for their potential to improve muscle mass, strength, and function in individuals with sarcopenia. Here’s an overview of the available and emerging options:  **1. Hormonal Therapies**   - **Testosterone Replacement Therapy (TRT):**   - May benefit men with clinically low testosterone levels (hypogonadism) by increasing muscle mass and strength.   - **Limitations:** Potential side effects (e.g., cardiovascular risks, prostate issues) and lack of evidence for use in men with normal testosterone levels. - **Selective Androgen Receptor Modulators (SARMs):**   - Experimental drugs that mimic the effects of testosterone on muscle growth without the same side effects.   - **Examples:** Enobosarm, LGD-4033.   - **Status:** Still under investigation and not FDA-approved for sarcopenia. - **Growth Hormone (GH) and IGF-1:**   - GH and insulin-like growth factor 1 (IGF-1) play a role in muscle maintenance, but their use is limited by side effects (e.g., fluid retention, joint pain).   - **Status:** Not routinely recommended for sarcopenia treatment.   **2. Myostatin Inhibitors**   - **Myostatin** is a protein that limits muscle growth. Inhibiting myostatin could potentially increase muscle mass. - **Examples:** Bimagrumab, Stamulumab. - **Status:** Experimental; clinical trials have shown mixed results, and these drugs are not yet approved.   **3. Vitamin D Supplementation**   - **Vitamin D deficiency** is associated with muscle weakness and increased risk of sarcopenia. - **Treatment:** Supplementation with vitamin D (e.g., 800–1000 IU/day) may improve muscle strength and function, especially in deficient individuals. - **Status:** Widely used as part of a broader sarcopenia management plan.   **4. Nutritional Supplements**   - **Creatine:**   - May enhance muscle strength and performance, particularly when combined with resistance training. - **Beta-Hydroxy-Beta-Methylbutyrate (HMB):**   - A metabolite of leucine that may help reduce muscle breakdown and support muscle mass. - **Branched-Chain Amino Acids (BCAAs):**   - Leucine, isoleucine, and valine may stimulate muscle protein synthesis. - **Status:** These supplements are not FDA-approved for sarcopenia but are commonly used as adjuncts to exercise and diet.   **5. Other Investigational Therapies**   - **Angiotensin-Converting Enzyme (ACE) Inhibitors:**   - Some studies suggest that ACE inhibitors (e.g., perindopril) may improve physical function in older adults, possibly by reducing inflammation and improving blood flow to muscles. - **Anti-Inflammatory Agents:**   - Chronic inflammation contributes to muscle loss. Anti-inflammatory drugs (e.g., NSAIDs) are being explored but are not yet recommended for sarcopenia. - **Senolytics:**   - Drugs that target senescent (aging) cells, which may contribute to muscle loss, are in early stages of research.   **6. Medications for Underlying Conditions**   - Managing chronic diseases (e.g., diabetes, heart disease) or hormonal imbalances (e.g., thyroid disorders) that contribute to muscle loss is an important part of sarcopenia treatment.   **Summary**  While there are no FDA-approved medications specifically for sarcopenia, several approaches are being explored:   - **Hormonal therapies (e.g., testosterone, SARMs),** - **Myostatin inhibitors,** - **Vitamin D supplementation,** - **Nutritional supplements (e.g., creatine, HMB).**   Currently, the most effective strategies remain **exercise** (especially resistance training) and **nutrition** (adequate protein and calorie intake). Emerging therapies hold promise but require further research and regulatory approval. Always consult a healthcare provider before starting any new medication or supplement. | While exercise and nutrition are the primary and most effective treatments for sarcopenia, several medications and supplements have been investigated for their potential to help manage or improve the condition. Here's an overview:  **1. FDA-Approved Medications:**   - Currently, there are **no medications specifically approved by the FDA** to treat sarcopenia directly.   **2. Investigational Medications:**   - **Selective Androgen Receptor Modulators (SARMs):** SARMs are a class of drugs that selectively bind to androgen receptors in muscle and bone tissue, promoting muscle growth and bone density without the side effects associated with traditional anabolic steroids. Several SARMs have been studied for their potential to treat sarcopenia, but none are currently FDA-approved for this purpose. - **Myostatin Inhibitors:** Myostatin is a protein that inhibits muscle growth. Myostatin inhibitors block the action of myostatin, allowing muscles to grow larger. Several myostatin inhibitors are in development for the treatment of sarcopenia and other muscle-wasting conditions. - **Ghrelin and Ghrelin Receptor Agonists:** Ghrelin is a hormone that stimulates appetite and growth hormone secretion. Ghrelin and ghrelin receptor agonists have been shown to increase muscle mass and strength in some studies.   **3. Supplements:**   - **Vitamin D:** Vitamin D deficiency is common in older adults and is associated with muscle weakness and an increased risk of falls. Vitamin D supplementation can improve muscle strength and function in individuals with vitamin D deficiency. - **Creatine:** Creatine is a naturally occurring compound that can enhance muscle strength and power. Creatine supplementation may be beneficial for individuals with sarcopenia, especially when combined with resistance training. - **Beta-hydroxy-beta-methylbutyrate (HMB):** HMB is a metabolite of the amino acid leucine that may help to preserve muscle mass and strength. HMB supplementation may be beneficial for individuals with sarcopenia, especially when combined with resistance training. - **Omega-3 Fatty Acids:** Omega-3 fatty acids, found in fatty fish, flaxseed, and walnuts, have anti-inflammatory properties and may help improve muscle protein synthesis. - **Essential Amino Acids (EAAs):** Supplementation with essential amino acids, particularly leucine, may stimulate muscle protein synthesis and help to preserve muscle mass.   **4. Hormone Replacement Therapy:**   - **Testosterone Replacement Therapy (TRT):** In some cases, testosterone replacement therapy may be considered for men with low testosterone levels and sarcopenia. TRT can increase muscle mass and strength, but it also has potential risks and side effects, such as prostate enlargement, acne, and mood changes. TRT should only be used under the guidance and supervision of a healthcare professional. - **Growth Hormone (GH):** Growth hormone can increase muscle mass, but it also has potential side effects, such as joint pain, fluid retention, and an increased risk of diabetes. Growth hormone is not generally recommended for the treatment of sarcopenia.   **Important Considerations:**   - **FDA Approval:** It's important to note that many of the medications and supplements mentioned above are not FDA-approved for the treatment of sarcopenia. - **Consult with a Healthcare Professional:** Before taking any medications or supplements for sarcopenia, it's essential to consult with your doctor or a qualified healthcare professional. They can help you determine if the treatment is appropriate for you and monitor you for any potential side effects. - **Lifestyle Modifications:** Medications and supplements should be used in conjunction with lifestyle modifications, such as exercise and proper nutrition, to achieve the best possible results.   In summary, while there are no FDA-approved medications specifically for sarcopenia, several medications and supplements are being investigated or used off-label for their potential benefits. The most effective approach to treating sarcopenia typically involves a combination of exercise, proper nutrition, and, in some cases, medication or supplementation, under the guidance of a healthcare professional.  Related searches:   - [medications for sarcopenia](https://www.google.com/search?q=medications+for+sarcopenia&client=app-vertex-grounding-quora-poe)   [supplements for sarcopenia](https://www.google.com/search?q=supplements+for+sarcopenia&client=app-vertex-grounding-quora-poe) | Currently, **no medication is specifically approved** by regulatory agencies (e.g., FDA, EMA) exclusively for the treatment of sarcopenia. However, several medications and supplements are used **off-label** or are under active investigation for their potential benefits.  **✅ Current Medication Options (Off-Label Use):**  Although not explicitly approved for sarcopenia, these medications may sometimes be prescribed or explored under medical supervision:  **1. Hormonal Therapies**   - **Testosterone Replacement Therapy (TRT)**   - **Use:** Beneficial in older men with low testosterone (hypogonadism).   - **Benefits:** Improves muscle mass, strength, and physical function.   - **Limitations:** Risks and side effects must be considered (e.g., prostate health, cardiovascular risk). - **Selective Estrogen Receptor Modulators (SERMs)**   - **Examples:** Raloxifene   - **Use:** May indirectly support muscle health via improving bone strength and hormone balance. - **Growth Hormone (GH)**   - Rarely used due to side effects and limited benefits; considered mostly experimental.   **2. Vitamin D Supplementation**   - Widely recommended in deficient individuals:   - Improves muscle strength, reduces fall risk.   - Typical dose: **800–2000 IU daily**.   **🚧 Medications Under Investigation (Experimental):**  Several emerging therapies are being studied but remain experimental:  **1. Myostatin Inhibitors**   - **Mechanism:** Blocks myostatin (a protein that limits muscle growth), potentially increasing muscle mass and strength. - Currently under clinical trials (not yet approved).   **2. Selective Androgen Receptor Modulators (SARMs)**   - **Mechanism:** Enhance muscle growth similarly to testosterone, but with potentially fewer side effects. - Currently under clinical trials and not approved for routine use.   **3. Anti-inflammatory Agents**   - **Examples:** NSAIDs, omega-3 fatty acids - **Mechanism:** Reduces chronic inflammation associated with muscle loss. - May indirectly help slow progression.   **💊 Medications to Avoid or Adjust (Related to Sarcopenia Risk):**   - Corticosteroids (prednisone) - Proton pump inhibitors (PPIs, e.g., omeprazole) - Chemotherapy drugs - Androgen deprivation therapy (ADT)   Adjusting or minimizing these medications can significantly reduce sarcopenia progression.  **📝 Current Best Practices (Non-medication approaches):**  Due to limited medication efficacy, **first-line treatments** still include:   - **Resistance (strength) exercise** - **Protein-rich diet** - **Vitamin D supplementation** - **Multidisciplinary interventions (Physical therapy, nutritionist guidance)**   **📌 Summary Table: Medication Options and Evidence:**   \| **Medication / Treatment** \| **Current Evidence** \| **Approval Status** \| \| --- \| --- \| --- \| \| **Testosterone Replacement** \| Effective in selected patients (men) \| Off-label use \| \| **Vitamin D** \| Effective if deficient \| Standard practice \| \| **Growth Hormone** \| Limited evidence, significant risks \| Rarely used, off-label \| \| **Myostatin inhibitors, SARMs** \| Promising, but experimental \| Under clinical trials \| \| **Anti-inflammatory drugs** \| May help indirectly \| Under investigation \|   **⚠️ Clinical Recommendation:**   - Currently, the best-supported treatments for sarcopenia remain **non-pharmacological**. - Medications should be considered only after thorough clinical evaluation and in select cases under specialist supervision.   **🔑 Conclusion:**  No medication is specifically approved yet, but some hormonal and nutritional supplements are beneficial. Experimental therapies are under active research. The most effective current treatment involves resistance exercise, nutrition optimization, and management of underlying conditions. |
| 18 | What types of exercise or nutrition are best for me to prevent or treat sarcopenia? | To **prevent or treat sarcopenia**, a combination of specific **exercise** and **nutrition** strategies is essential. These approaches help maintain or increase muscle mass, strength, and function. Here’s a detailed guide tailored to your needs:  **Exercise Recommendations**  Exercise is the cornerstone of sarcopenia prevention and treatment. Focus on the following types:  **1. Resistance Training (Strength Training)**   - **Why?** Stimulates muscle protein synthesis, builds muscle mass, and improves strength. - **Examples:**   - Weightlifting (e.g., dumbbells, barbells, kettlebells),   - Resistance band exercises,   - Bodyweight exercises (e.g., squats, lunges, push-ups). - **Frequency:** 2–3 times per week, targeting all major muscle groups. - **Tips:**   - Start with light weights and gradually increase intensity.   - Focus on proper form to avoid injury.   - Work with a trainer or physical therapist if needed.   **2. Aerobic Exercise (Cardio)**   - **Why?** Improves cardiovascular health, endurance, and overall muscle function. - **Examples:**   - Walking, jogging, cycling, swimming, dancing. - **Frequency:** 150 minutes of moderate-intensity aerobic activity per week (e.g., 30 minutes, 5 days a week). - **Tips:**   - Choose activities you enjoy to stay consistent.   - Incorporate interval training (alternating high and low intensity) for added benefits.   **3. Balance and Flexibility Training**   - **Why?** Enhances mobility, reduces the risk of falls, and supports overall physical function. - **Examples:**   - Yoga, tai chi, stretching routines. - **Frequency:** 2–3 times per week, or as part of a daily routine. - **Tips:**   - Focus on exercises that improve core strength and stability.   - Use props (e.g., yoga blocks, straps) for support if needed.   **Nutrition Recommendations**  Proper nutrition is critical for muscle maintenance and repair. Focus on the following:  **1. Adequate Protein Intake**   - **Why?** Protein provides the building blocks (amino acids) for muscle repair and growth. - **Daily Goal:** 1.0–1.2 g of protein per kg of body weight (or up to 1.5 g/kg for those with sarcopenia). - **Sources:**   - Lean meats (chicken, turkey, beef),   - Fish (salmon, tuna, cod),   - Eggs,   - Dairy (milk, yogurt, cheese),   - Plant-based options (tofu, tempeh, lentils, beans, quinoa). - **Tips:**   - Distribute protein intake evenly across meals (e.g., 25–30 g per meal).   - Include a protein-rich snack (e.g., Greek yogurt, nuts, or a protein shake).   **2. Essential Amino Acids (Leucine)**   - **Why?** Leucine, a branched-chain amino acid, is particularly important for stimulating muscle protein synthesis. - **Sources:** Eggs, dairy, soy, chicken, fish, beef. - **Tips:** Include leucine-rich foods in your diet, especially after exercise.   **3. Vitamin D**   - **Why?** Vitamin D deficiency is linked to muscle weakness and increased risk of sarcopenia. - **Daily Goal:** 800–1000 IU/day (or as advised by a healthcare provider). - **Sources:**   - Sunlight exposure,   - Fortified foods (milk, orange juice, cereals),   - Fatty fish (salmon, mackerel),   - Supplements (if deficient). - **Tips:** Have your vitamin D levels checked and supplement if necessary.   **4. Omega-3 Fatty Acids**   - **Why?** Omega-3s reduce inflammation and may support muscle health. - **Sources:**   - Fatty fish (salmon, mackerel, sardines),   - Flaxseeds, chia seeds, walnuts,   - Fish oil supplements. - **Tips:** Aim for 2–3 servings of fatty fish per week or consider a supplement.   **5. Balanced Diet**   - **Why?** A nutrient-rich diet supports overall health and muscle function. - **Focus:**   - Fruits and vegetables (for antioxidants and micronutrients),   - Whole grains (for energy and fiber),   - Healthy fats (e.g., olive oil, avocados, nuts). - **Tips:** Avoid processed foods and excessive sugar.   **Lifestyle Tips**   1. **Stay Active:** Avoid prolonged sitting. Incorporate movement into your daily routine (e.g., walking, stretching). 2. **Hydration:** Drink plenty of water to support muscle function and recovery. 3. **Sleep:** Aim for 7–9 hours of quality sleep per night to allow muscles to repair and recover. 4. **Manage Stress:** Chronic stress can contribute to muscle loss. Practice relaxation techniques (e.g., meditation, deep breathing).   **Sample Daily Plan**   - **Breakfast:** Greek yogurt with berries and a sprinkle of nuts. - **Snack:** Hard-boiled eggs or a protein shake. - **Lunch:** Grilled chicken salad with quinoa and olive oil dressing. - **Snack:** Apple slices with almond butter. - **Dinner:** Baked salmon with steamed vegetables and brown rice. - **Exercise:** 30 minutes of resistance training (e.g., weightlifting) + 30 minutes of walking.   **When to Consult a Professional**   - If you’re new to exercise, consider working with a physical therapist or certified trainer. - A registered dietitian can help create a personalized nutrition plan. - Consult your doctor if you have underlying medical conditions or are considering supplements.   By combining **resistance training**, **aerobic exercise**, and a **protein-rich, nutrient-dense diet**, you can effectively prevent or treat sarcopenia and maintain muscle health as you age. Consistency is key! | To best prevent or treat sarcopenia, a combination of specific exercises and nutritional strategies is most effective. Here's a detailed breakdown tailored to your needs:  **Exercise Recommendations:**   - **Resistance Training (Strength Training):** This is the most critical type of exercise for preventing and treating sarcopenia.   - **How it Works:** Resistance training stimulates muscle protein synthesis, which helps build and maintain muscle mass and strength.   - **Exercise Examples:**     - **Weightlifting:** Using free weights (dumbbells, barbells) or weight machines. Examples include squats, deadlifts, bench press, overhead press, rows, bicep curls, and triceps extensions.     - **Resistance Bands:** Using elastic bands to provide resistance. These are a good option if you're new to exercise or have limited mobility.     - **Bodyweight Exercises:** Using your own body weight for resistance. Examples include squats, lunges, push-ups, planks, and calf raises.   - **Intensity:** Use a weight or resistance that challenges you but allows you to maintain good form. You should feel fatigued by the end of each set.   - **Sets and Reps:** Aim for 2-3 sets of 8-12 repetitions for each exercise.   - **Frequency:** Perform resistance training exercises at least two to three times per week, with rest days in between to allow your muscles to recover.   - **Progression:** Gradually increase the weight, resistance, or number of repetitions as you get stronger. - **Aerobic Exercise:** While not as effective as resistance training for building muscle mass, aerobic exercise is still important for overall health and fitness.   - **How it Works:** Aerobic exercise improves cardiovascular health, endurance, and overall fitness, which can indirectly support muscle health and function.   - **Exercise Examples:** Walking, jogging, swimming, cycling, dancing.   - **Intensity:** Aim for moderate-intensity aerobic exercise, where you can talk but not sing.   - **Duration:** Aim for at least 150 minutes of moderate-intensity or 75 minutes of vigorous-intensity aerobic exercise per week.   - **Frequency:** Spread your aerobic exercise throughout the week. - **Balance and Flexibility Exercises:** Incorporating balance and flexibility exercises can improve stability, coordination, and range of motion, reducing the risk of falls and injuries.   - **Exercise Examples:** Yoga, tai chi, Pilates, stretching exercises.   - **Frequency:** Perform balance and flexibility exercises at least two to three times per week.   **Nutritional Recommendations:**   - **Adequate Protein Intake:** Protein is essential for muscle protein synthesis.   - **How it Works:** Protein provides the building blocks (amino acids) needed to repair and rebuild muscle tissue.   - **Recommended Intake:** Aim for a daily protein intake of 1.0-1.2 grams per kilogram of body weight, or even higher (1.2-1.5 g/kg) for older adults or those with increased protein needs.   - **Distribution:** Distribute your protein intake evenly throughout the day, rather than consuming most of it in one meal. This can help maximize muscle protein synthesis.   - **Sources:** Choose high-quality protein sources, such as lean meats, poultry, fish, eggs, dairy products, legumes, and soy products. - **Sufficient Caloric Intake:** Ensure you are consuming enough calories to meet your energy needs. Chronic undereating can lead to muscle loss. - **Vitamin D and Calcium:**   - **Vitamin D:** Vitamin D plays a crucial role in muscle function and bone health. Get your vitamin D levels checked and take supplements if needed to maintain adequate levels (typically above 30 ng/mL).   - **Calcium:** Calcium is important for bone health and muscle function. Include calcium-rich foods in your diet, such as dairy products, leafy green vegetables, and fortified foods. - **Creatine Supplementation:** Creatine is a naturally occurring compound that can enhance muscle strength and power. Creatine supplementation may be beneficial, especially when combined with resistance training. - **Beta-hydroxy-beta-methylbutyrate (HMB):** HMB is a metabolite of the amino acid leucine that may help to preserve muscle mass and strength. HMB supplementation may be beneficial, especially when combined with resistance training. - **Omega-3 Fatty Acids:** Omega-3 fatty acids, found in fatty fish, flaxseed, and walnuts, have anti-inflammatory properties and may help improve muscle protein synthesis. - **Leucine:** Leucine is an essential amino acid that plays a key role in stimulating muscle protein synthesis. Consider supplementing with leucine or consuming foods that are rich in leucine, such as dairy products, meat, and eggs. - **Hydration:** Drink plenty of water throughout the day to stay hydrated.   **Additional Tips:**   - **Progressive Overload:** To continue seeing results, gradually increase the intensity, duration, or frequency of your workouts over time. - **Proper Form:** Focus on using proper form when performing exercises to prevent injuries. - **Listen to Your Body:** Pay attention to your body and rest when you need to. - **Stay Consistent:** Consistency is key to seeing results. Make exercise and proper nutrition a part of your daily routine.   **Personalized Approach:**   - **Consult with a Healthcare Professional:** It's always best to consult with your doctor, a registered dietitian, or a certified personal trainer to develop a personalized exercise and nutrition plan that is tailored to your individual needs and goals. - **Consider Your Current Fitness Level:** If you're new to exercise, start slowly and gradually increase the intensity and duration of your workouts. - **Address Any Underlying Health Conditions:** If you have any underlying health conditions, such as arthritis, heart disease, or diabetes, be sure to discuss them with your doctor before starting a new exercise program. - **Listen to Your Body:** Pay attention to your body and rest when you need to. Don't push yourself too hard, especially when you're first starting out. - **Make it Sustainable:** Choose exercises and foods that you enjoy so that you're more likely to stick with your plan long-term.   By following these recommendations, you can effectively prevent or treat sarcopenia and maintain your muscle health and function as you age.  Related searches:   - [best exercise for sarcopenia](https://www.google.com/search?q=best+exercise+for+sarcopenia&client=app-vertex-grounding-quora-poe)   [nutrition for sarcopenia](https://www.google.com/search?q=nutrition+for+sarcopenia&client=app-vertex-grounding-quora-poe) | To effectively **prevent or treat sarcopenia**, the best strategy is combining **resistance exercise** with appropriate **nutritional interventions**. Here’s a clear, personalized guide for optimal outcomes:  **🏋️‍♀️ Best Exercise Recommendations:**  **1. Resistance (Strength) Training *(Highest Priority)***   - **Frequency:** 2–3 times per week - **Intensity:** Moderate to high (challenging but comfortable), typically 8–12 repetitions per set - **Examples:**   - **Lower body:** Squats, lunges, leg press, calf raises   - **Upper body:** Push-ups, shoulder presses, rows, bicep curls, chest press   - **Core strength:** Planks, abdominal exercises, bridges   - **Equipment:** Free weights, resistance bands, weight machines, bodyweight exercises   **2. Aerobic Exercise *(Secondary Priority)***   - **Frequency:** At least 150 minutes/week (30 min/day, 5 days/week) - **Intensity:** Moderate (e.g., brisk walking, cycling, swimming, elliptical machine) - **Benefits:** Improves cardiovascular health, endurance, reduces inflammation   **3. Balance and Flexibility Exercises *(Supportive Priority)***   - **Frequency:** 2–3 times per week - **Examples:** Yoga, tai chi, stretching routines - **Benefits:** Improved coordination, reduced fall risk, enhanced mobility   **🍽️ Best Nutritional Recommendations:**  **1. Protein Intake (Critical)**   - **Daily target:** 1.0–1.5 grams protein per kilogram of body weight per day *(Example: 60 kg individual = 60–90 g protein/day)* - **High-quality sources:**   - Animal proteins: Lean meats (chicken, turkey), eggs, fish, dairy (milk, yogurt, cottage cheese)   - Plant proteins: Beans, lentils, tofu, quinoa, nuts, seeds, soy products   **2. Vitamin D and Calcium**   - Essential for muscle and bone health - Recommended daily Vitamin D intake: **800–2000 IU/day** (higher if deficient) - Calcium intake: **1000–1200 mg/day** - Sources: Dairy products, fortified foods, supplements if needed   **3. Omega-3 Fatty Acids**   - Anti-inflammatory benefits and muscle health improvement - Sources: Fatty fish (salmon, tuna, sardines), flaxseeds, chia seeds, walnuts - Supplementation (fish oil) if dietary intake is insufficient   **4. Antioxidants and Nutrient-Dense Foods**   - Include fruits, vegetables, whole grains, nuts, seeds, and legumes daily - Reduces inflammation and supports overall muscle health   **✅ Personalized Recommendations:**  Here’s an **optimal weekly plan** to prevent or treat sarcopenia:   \| **Component** \| **Frequency** \| **Specific Recommendations** \| \| --- \| --- \| --- \| \| Resistance Training \| **2–3 sessions/week** \| Full-body resistance exercises \| \| Aerobic Exercise \| **≥150 min/week** \| Walking, cycling, swimming \| \| Balance/Flexibility \| **2–3 sessions/week** \| Yoga, stretching, tai chi \| \| Protein Intake \| **Daily (1.0–1.5 g/kg/day)** \| Mix of animal & plant-based protein \| \| Vitamin D/Calcium \| **Daily (800–2000 IU, 1000–1200 mg)** \| Through diet or supplementation \| \| Omega-3 Intake \| **2–3 servings/week** \| Fish or supplements \| \| Antioxidants \| **Daily intake** \| Fruits, vegetables, whole grains \|   **⚠️ Practical Tips:**   - **Start slowly:** Gradually increase exercise intensity and duration to prevent injury. - **Consistency:** Regular exercise (even small amounts daily) is key to improvement. - **Dietitian consultation:** Helpful for personalized nutritional planning. - **Professional support:** Consider guidance from physical therapists or certified trainers.   **🩺 Clinical Note:**  This combined **exercise and nutritional approach** is the **most effective and evidence-based method** to prevent, delay, or treat sarcopenia, improving strength, function, and quality of life. |
| **Topic: Prognosis** | |  |  |  |
| 19 | Can sarcopenia lead to fall, disability, or fractures? | Yes, **sarcopenia** (age-related loss of muscle mass, strength, and function) is strongly associated with an increased risk of **falls**, **disability**, and **fractures**. Here’s how sarcopenia contributes to these outcomes and why it’s crucial to address it proactively:  **1. Increased Risk of Falls**   - **Muscle Weakness:** Sarcopenia leads to reduced muscle strength, particularly in the legs, which impairs balance and stability. - **Poor Mobility:** Loss of muscle mass and function can make it harder to perform daily activities like walking, climbing stairs, or standing up from a chair. - **Decreased Reaction Time:** Weakened muscles may slow down the body’s ability to react to sudden changes in balance, increasing the likelihood of falls.   **2. Higher Risk of Fractures**   - **Fall-Related Fractures:** Falls are a leading cause of fractures, especially in older adults. Sarcopenia increases the risk of falls, which can result in fractures of the hip, wrist, or spine. - **Osteoporosis Connection:** Sarcopenia often coexists with osteoporosis (low bone density), further increasing the risk of fractures even from minor falls. - **Fragility Fractures:** Reduced muscle mass and strength can lead to fragility fractures, where bones break under minimal stress (e.g., from a slight bump or fall from standing height).   **3. Disability and Loss of Independence**   - **Reduced Physical Function:** Sarcopenia makes it harder to perform activities of daily living (ADLs), such as dressing, bathing, or cooking, leading to a loss of independence. - **Functional Decline:** Over time, muscle weakness can progress to mobility issues, requiring assistive devices (e.g., walkers, canes) or assistance from caregivers. - **Frailty:** Sarcopenia is a key component of frailty, a syndrome characterized by weakness, fatigue, and increased vulnerability to adverse health outcomes.   **4. Cascade of Health Problems**   - **Hospitalization:** Falls and fractures often lead to hospitalizations, which can further accelerate muscle loss due to immobility and bed rest. - **Complications:** Prolonged immobility from fractures or disability can increase the risk of complications such as pressure ulcers, infections, and deep vein thrombosis (DVT). - **Mental Health Impact:** Loss of independence and physical function can lead to depression, anxiety, and a reduced quality of life.   **How to Reduce the Risk**  To mitigate the risks associated with sarcopenia, focus on the following strategies:  **1. Exercise**   - **Resistance Training:** Builds muscle mass and strength. - **Aerobic Exercise:** Improves cardiovascular health and endurance. - **Balance and Flexibility Training:** Enhances stability and reduces fall risk.   **2. Nutrition**   - **Adequate Protein Intake:** Supports muscle repair and maintenance. - **Vitamin D and Calcium:** Strengthen bones and reduce fracture risk. - **Balanced Diet:** Provides essential nutrients for overall health.   **3. Lifestyle Modifications**   - **Avoid Sedentary Behavior:** Stay active throughout the day. - **Fall Prevention:** Remove tripping hazards at home, use assistive devices if needed, and ensure proper lighting. - **Regular Health Checkups:** Monitor muscle mass, bone density, and overall health.   **4. Medical Management**   - **Address Underlying Conditions:** Manage chronic diseases (e.g., diabetes, heart disease) that contribute to muscle loss. - **Medication Review:** Avoid medications that may increase fall risk (e.g., sedatives, blood pressure drugs).   **Summary**  Sarcopenia significantly increases the risk of **falls**, **fractures**, and **disability**, which can lead to a cascade of health problems and loss of independence. However, these risks can be reduced through:   - **Regular exercise** (especially resistance and balance training), - **Proper nutrition** (high-quality protein, vitamin D, and calcium), - **Lifestyle changes** (fall prevention, staying active), - **Medical management** of underlying conditions.   Early intervention and consistent adherence to these strategies are key to maintaining muscle health and preventing the adverse outcomes associated with sarcopenia. | Yes, sarcopenia can significantly increase the risk of falls, disability, and fractures. Here's how:   - **Falls:**   - **Muscle Weakness:** Sarcopenia is characterized by a decline in muscle mass and strength. Weak muscles, particularly in the legs and core, make it harder to maintain balance and stability, increasing the likelihood of falls.   - **Impaired Balance:** Muscle weakness can also impair balance and coordination, making it more difficult to recover from a loss of balance.   - **Slowed Reaction Time:** Sarcopenia can slow down reaction time, making it harder to react quickly to prevent a fall. - **Disability:**   - **Reduced Physical Function:** Sarcopenia can lead to a decline in physical function, making it difficult to perform daily activities such as walking, climbing stairs, lifting objects, and getting out of a chair.   - **Loss of Independence:** As physical function declines, individuals with sarcopenia may become increasingly dependent on others for assistance with daily tasks, leading to a loss of independence.   - **Increased Risk of Hospitalization:** Sarcopenia is associated with an increased risk of hospitalization and institutionalization. - **Fractures:**   - **Increased Risk of Falls:** As mentioned above, sarcopenia increases the risk of falls, which can lead to fractures.   - **Reduced Bone Density:** Sarcopenia is often associated with reduced bone density (osteoporosis or osteopenia), which makes bones more fragile and susceptible to fractures.   - **Impaired Muscle Strength:** Strong muscles help to protect bones from injury during a fall. Sarcopenia reduces muscle strength, making bones more vulnerable to fractures. - **Increased Mortality:**   - **Overall increased risk:** Sarcopenia is associated with an increased risk of mortality.   In summary, sarcopenia is a serious condition that can have a significant impact on your health and quality of life. By taking steps to prevent or treat sarcopenia, you can reduce your risk of falls, disability, fractures, and other adverse health outcomes.  Related searches:   - [sarcopenia falls fractures disability](https://www.google.com/search?q=sarcopenia+falls+fractures+disability&client=app-vertex-grounding-quora-poe)   [sarcopenia complications](https://www.google.com/search?q=sarcopenia+complications&client=app-vertex-grounding-quora-poe) | Yes, **sarcopenia** significantly increases the risk of **falls**, **disability**, and **fractures**, particularly among older adults. Here's how these outcomes are closely interconnected:  **✅ How Sarcopenia Leads to Falls, Disability, and Fractures:**  **🔹 1. Increased Risk of Falls**   - **Reduced muscle strength**: Impaired leg strength and coordination significantly decrease balance and stability. - **Poor balance and coordination**: Difficulty maintaining posture increases the likelihood of stumbling or falling. - **Slowed reaction time**: Difficulty reacting quickly to prevent or reduce the severity of falls.   **🔹 2. Increased Disability and Loss of Independence**   - **Reduced functional mobility**: Difficulty performing essential daily tasks, such as climbing stairs, standing up from chairs, and walking short distances. - **Dependence on caregivers or assistive devices**: Inability to independently perform daily activities, increasing reliance on others. - **Loss of quality of life**: Progressive disability can significantly reduce social engagement, psychological well-being, and overall quality of life.   **🔹 3. Increased Risk of Fractures**   - **Falls combined with osteoporosis (common coexisting condition)**:   - High risk of fractures due to fragile bones coupled with muscle weakness. - **Hip fractures**:   - Particularly severe, often resulting in hospitalization, long-term disability, or even increased mortality. - **Other fractures** (wrist, vertebrae, shoulder):   - Increased prevalence in older adults with sarcopenia due to compromised stability and protective muscle mass.   **⚠️ Clinical Significance:**   \| **Outcome** \| **Sarcopenia's Contribution** \| **Clinical Impact** \| \| --- \| --- \| --- \| \| **Falls** \| Muscle weakness, impaired balance, instability \| Increased injuries, hospitalizations \| \| **Disability** \| Reduced functional ability, mobility \| Dependence, decreased independence \| \| **Fractures** \| Increased fall risk + osteoporosis \| Hospitalizations, chronic pain, increased mortality \|   **🚩 Evidence Summary:**   - Research consistently demonstrates a strong association between sarcopenia and higher rates of falls, fractures (especially hip fractures), hospitalization, and disability. - Individuals with sarcopenia are significantly more likely to experience loss of independence and poor quality of life.   **📌 Prevention and Intervention Recommendations:**  To reduce these risks effectively:   - **Resistance training** to increase muscle mass, strength, and stability. - **Balance and coordination exercises** (e.g., Tai chi, yoga, specific balance training). - **Adequate protein intake, calcium, and vitamin D** to support muscle and bone health. - **Management of osteoporosis**, if present, to further reduce fracture risk.   **🔑 Conclusion:**  Sarcopenia strongly contributes to the risks of falls, fractures, and disability. Early diagnosis, preventative interventions, and proper management are essential to minimizing these severe health impacts and maintaining independence and quality of life. |
| 20 | How does sarcopenia impact my daily life? | **Sarcopenia**, the age-related loss of muscle mass, strength, and function, can significantly impact your daily life, especially if left unmanaged. Here’s how it may affect you and what you can do to mitigate its effects: **1. Reduced Mobility**  - **Difficulty Walking**: Slower walking speed or trouble walking long distances. - **Dependence on Assistive Devices**: Needing a cane, walker, or wheelchair to move around. - **Limited Range of Motion**: Difficulty bending, reaching, or lifting objects.  **2. Loss of Independence**  - **Difficulty with Daily Activities**:   - Struggling with tasks like dressing, bathing, cooking, or cleaning. - **Need for Assistance**:   - Relying on family members or caregivers for help with daily tasks. - **Inability to Live Alone**:   - Moving to assisted living or a care facility due to reduced independence.  **3. Increased Fatigue**  - **Low Energy Levels**: Feeling tired even after minimal physical activity. - **Reduced Stamina**: Needing frequent rest breaks during daily tasks.  **4. Higher Fall Risk**  - **Weak Muscles and Poor Balance**: Increased likelihood of falls, which can lead to fractures or injuries. - **Fear of Falling**: Limiting physical activity due to fear, which can worsen muscle loss.  **5. Emotional and Psychological Impact**  - **Depression and Anxiety**: Feeling sad, hopeless, or anxious about loss of independence or physical abilities. - **Social Isolation**: Withdrawing from social activities due to physical limitations or embarrassment. - **Reduced Confidence**: Feeling less capable or confident in your abilities.  **6. Chronic Pain**  - **Joint and Muscle Pain**: Weak muscles can place more strain on joints, leading to discomfort. - **Posture Issues**: Hunched posture or back pain due to weakened core muscles.  **7. Nutritional Challenges**  - **Difficulty Eating**: Weakness or fatigue may make it harder to prepare meals or eat independently. - **Malnutrition**: Poor appetite or difficulty eating can lead to nutrient deficiencies.  **8. Increased Healthcare Needs**  - **Frequent Medical Visits**: Managing complications like fractures, infections, or chronic conditions. - **Rehabilitation**: Physical therapy or occupational therapy to regain function.  **9. Reduced Quality of Life**  - **Loss of Enjoyment**: Difficulty participating in hobbies or activities you once enjoyed. - **Strained Relationships**: Increased reliance on family or caregivers can strain relationships.  **How to Mitigate the Impact of Sarcopenia**  1. **Exercise Regularly**:    - **Resistance Training**: Build muscle with weights, resistance bands, or bodyweight exercises.    - **Aerobic Exercise**: Improve cardiovascular health with walking, swimming, or cycling.    - **Balance Training**: Reduce fall risk with yoga or tai chi. 2. **Optimize Nutrition**:    - **High-Protein Diet**: Include lean meats, fish, eggs, dairy, legumes, and plant-based proteins.    - **Essential Nutrients**: Ensure adequate intake of vitamin D, calcium, and omega-3 fatty acids.    - **Supplements**: Consider protein shakes or vitamin D supplements if dietary intake is insufficient. 3. **Make Lifestyle Changes**:    - **Stay Active**: Avoid prolonged sitting and incorporate movement into your daily routine.    - **Quit Smoking**: Smoking accelerates muscle loss.    - **Limit Alcohol**: Excessive alcohol consumption can impair muscle repair.    - **Sleep Well**: Aim for 7–9 hours of quality sleep per night to support muscle recovery. 4. **Prevent Falls**:    - **Home Modifications**: Install grab bars, remove tripping hazards, and improve lighting.    - **Assistive Devices**: Use canes, walkers, or other devices if needed for stability. 5. **Seek Support**:    - **Physical Therapy**: Work with a therapist to improve strength and mobility.    - **Mental Health**: Seek counseling or therapy if you’re feeling anxious or depressed.    - **Support Groups**: Connect with others experiencing sarcopenia for shared experiences and encouragement. 6. **Monitor Progress**:    - **Regular Check-Ups**: Track muscle mass, strength, and physical performance over time.    - **Adjust Interventions**: Modify your exercise or nutrition plan based on progress and feedback.  **Summary** Sarcopenia can significantly impact your daily life by reducing mobility, independence, and quality of life. However, proactive measures like **exercise**, **proper nutrition**, **lifestyle changes**, and **fall prevention** can help manage the condition and improve your overall well-being. If you’re concerned about sarcopenia, consult a healthcare provider for personalized advice and interventions. | Sarcopenia can significantly impact your daily life in various ways due to the loss of muscle mass and strength. Here's a breakdown of the potential effects:  **1. Physical Limitations and Reduced Mobility:**   - **Difficulty with Everyday Tasks:** You may find it harder to perform common activities such as:   - Walking, especially for longer distances or on uneven surfaces   - Climbing stairs   - Lifting or carrying groceries or other objects   - Getting up from a chair or bed   - Bending over to pick things up - **Increased Fatigue:** Muscle weakness can lead to feeling tired and drained more easily, even after light activity. - **Slower Movement:** You may notice that you move more slowly and deliberately, affecting your ability to keep up with others or complete tasks quickly. - **Balance Problems:** Loss of muscle strength, especially in the legs and core, can impair balance, increasing the risk of falls.   **2. Decreased Independence:**   - **Need for Assistance:** As daily tasks become more challenging, you might require help from family members, friends, or caregivers with activities you used to do independently. - **Reduced Social Engagement:** Physical limitations can make it harder to participate in social activities, leading to isolation and loneliness. - **Difficulty with Self-Care:** In severe cases, sarcopenia can make it difficult to perform basic self-care tasks such as bathing, dressing, and grooming.   **3. Increased Risk of Falls and Injuries:**   - **Higher Fall Rate:** Muscle weakness and impaired balance significantly increase the risk of falls, which can lead to serious injuries. - **Fractures:** Sarcopenia is often associated with reduced bone density, making bones more susceptible to fractures in the event of a fall. - **Longer Recovery Time:** If you experience an injury, the recovery process may be slower and more challenging due to reduced muscle mass and strength.   **4. Reduced Quality of Life:**   - **Loss of Confidence:** Physical limitations and dependence on others can lead to a loss of self-esteem and confidence. - **Depression and Anxiety:** Sarcopenia can contribute to feelings of sadness, hopelessness, and worry. - **Reduced Overall Well-being:** The combined effects of physical limitations, decreased independence, and increased risk of falls can significantly diminish your overall quality of life.   **5. Increased Healthcare Costs:**   - **More Frequent Doctor Visits:** Sarcopenia may require more frequent visits to healthcare providers for monitoring and treatment. - **Hospitalizations:** The increased risk of falls and fractures can lead to hospitalizations and associated medical expenses. - **Long-Term Care:** In severe cases, sarcopenia may necessitate long-term care in a nursing home or assisted living facility.   **6. Impact on Chronic Conditions:**   - **Worsening of Existing Conditions:** Sarcopenia can exacerbate existing chronic conditions such as arthritis, heart disease, and diabetes. - **Increased Risk of Complications:** Sarcopenia can increase the risk of complications from other medical conditions. - **Increased Mortality:** Sarcopenia can increase the risk of mortality.   It's important to note that the severity of these impacts can vary depending on the individual and the extent of their sarcopenia. However, by taking steps to prevent or treat sarcopenia, you can minimize these negative effects and maintain a higher quality of life.  Related searches:   - [sarcopenia quality of life](https://www.google.com/search?q=sarcopenia+quality+of+life&client=app-vertex-grounding-quora-poe)   [impact of sarcopenia on daily living](https://www.google.com/search?q=impact+of+sarcopenia+on+daily+living&client=app-vertex-grounding-quora-poe) | Sarcopenia, characterized by a gradual loss of muscle mass, strength, and function, can significantly impact your daily life in various ways:  **1. Reduced Strength and Endurance**   - Difficulty lifting objects, climbing stairs, or doing household chores. - Increased fatigue and feeling easily tired.   **2. Decreased Mobility**   - Slowed walking speed and reduced balance, making it harder to maintain independence. - Increased risk of falls and related injuries.   **3. Challenges in Performing Daily Tasks**   - Struggles with simple activities like standing from chairs, dressing, or cooking. - Reliance on assistance or mobility aids (canes, walkers).   **4. Lower Quality of Life**   - Reduced participation in social or leisure activities, leading to isolation. - Feelings of dependency or reduced self-confidence.   **5. Increased Health Risks**   - Higher risk of osteoporosis and fractures due to reduced muscle support. - Increased vulnerability to chronic conditions like diabetes and cardiovascular diseases.   **6. Changes in Body Composition**   - Increased body fat percentage, leading to metabolic issues. - Weight gain or obesity due to reduced physical activity.   **How to Minimize the Impact:**   - Regular **strength training and resistance exercises** to build muscle. - Adequate **protein and nutrient intake** to maintain muscle mass. - Early screening and diagnosis to manage the condition proactively.   If you have concerns about sarcopenia, consult a healthcare provider or physiotherapist to help develop a tailored exercise and nutrition plan. |

Supplementary Table 3: Raw Score and Rating of each LLM-Chatbot

|  |  | **Deepseek** | | | **Gemini** | | | **ChatGPT** | | |
| --- | --- | --- | --- | --- | --- | --- | --- | --- | --- | --- |
| **Domain** |  | **Score Sum (Acc) Max: 12** | **Majority Consensus (Acc)** | **Average Score (Comp) Max: 5** | **Score Sum (Acc) Max: 12** | **Majority Consensus (Acc)** | **Average Score (Comp) Max: 5** | **Score Sum (Acc) Max: 12** | **Majority Consensus (Acc)** | **Average Score (Comp) Max: 5** |
| **Pathogenesis** | 1.00 | 8 | Good | 3.67 | 9 | Good | 3.33 | 11 | Excellent | 3.67 |
|  | 2.00 | 10 | Good | 4.00 | 9 | Good | 3.33 | 9 | Good | 3.33 |
|  | 3.00 | 10 | Good | 4.00 | 10 | Good | 4.00 | 10 | Good | 3.67 |
|  | 4.00 | 11 | Excellent | 4.00 | 10 | Good | 4.00 | 10 | Good | 4.33 |
|  | 5.00 | 9 | Good | 4.00 | 9 | Good | 3.67 | 9 | Good | 4.00 |
| **Risk factors** | 1.00 | 11 | Excellent | 4.00 | 10 | Good | 4.00 | 8 | Moderate | 3.67 |
|  | 2.00 | 11 | Excellent | 4.33 | 10 | Excellent | 4.33 | 10 | Good | 4.33 |
|  | 3.00 | 11 | Excellent | 4.33 | 10 | Good | 4.00 | 10 | Excellent | 4.00 |
|  | 4.00 | 10 | Good | 3.67 | 9 | Good | 3.67 | 8 | Good | 3.33 |
|  | 5.00 | 10 | Good | 3.67 | 10 | Good | 3.67 | 9 | Good | 3.67 |
| **Clinical presentation** | 1.00 | 9 | Good | 3.67 | 11 | Excellent | 4.00 | 11 | Excellent | 4.00 |
|  | 2.00 | 9 | Good | 4.00 | 10 | Excellent | 4.67 | 10 | Good | 4.33 |
| **Diagnosis** | 1.00 | 9 | Good | 4.00 | 10 | Good | 3.67 | 10 | Good | 3.67 |
|  | 2.00 | 10 | Excellent | 4.00 | 11 | Excellent | 4.00 | 11 | Excellent | 4.33 |
| **Prevention and treatment** | 1.00 | 11 | Excellent | 4.00 | 10 | Excellent | 4.33 | 10 | Good | 4.00 |
|  | 2.00 | 11 | Excellent | 4.33 | 10 | Excellent | 4.33 | 10 | Excellent | 4.33 |
|  | 3.00 | 10 | Excellent | 4.33 | 9 | Good | 4.00 | 11 | Excellent | 4.33 |
|  | 4.00 | 9 | Good | 4.00 | 10 | Good | 4.00 | 10 | Good | 3.67 |
| **Prognosis** | 1.00 | 11 | Excellent | 4.00 | 11 | Excellent | 4.33 | 11 | Excellent | 4.00 |
|  | 2.00 | 11 | Excellent | 4.33 | 9 | Good | 4.00 | 11 | Excellent | 4.00 |
